# Public health impact and cost-effectiveness of gonorrhoea vaccination: an integrated transmission-dynamic health-economic modelling analysis

**DOI:** 10.1016/S1473-3099(21)00744-1

**Published:** 2022-07

**Authors:** Lilith K Whittles, Xavier Didelot, Peter J White

**Affiliations:** a Department of Infectious Disease Epidemiology, School of Public Health, Imperial College London, London, UK; b MRC Centre for Global Infectious Disease Analysis, School of Public Health, Imperial College London, London, UK; c NIHR Health Protection Research Unit in Modelling and Health Economics, School of Public Health, Imperial College London, London, UK; d School of Life Sciences, University of Warwick, Coventry, UK; e Department of Statistics, University of Warwick, Coventry, UK; f NIHR Health Protection Research Unit in Genomics and Enabling Data, University of Warwick, Coventry, UK; g Modelling and Economics Unit, National Infection Service, Public Health England, London, UK

## Abstract

**Background:**

Gonorrhoea is a rapidly growing public health threat, with rising incidence and increasing drug resistance. Evidence that the MeNZB and four-component serogroup B meningococcal (4CMenB) vaccines, designed against *Neisseria meningitidis*, can also offer protection against gonorrhoea has created interest in using 4CMenB for this purpose and for developing gonorrhoea-specific vaccines. However, cost-effectiveness, and how the efficacy and duration of protection affect a gonorrhoea vaccine's value, have not been assessed.

**Methods:**

We developed an integrated transmission-dynamic health-economic model, calibrated using Bayesian methods to surveillance data (from the Genitourinary Medicine Clinic Activity Dataset and the Gonococcal Resistance to Antimicrobials Surveillance Programme) on men who have sex with men (MSM) in England. We considered vaccination of MSM from the perspective of sexual health clinics, with and without vaccination offered to all adolescents in schools (vaccination before entry [VbE]), comparing three realistic approaches to targeting: vaccination on attendance (VoA) for testing; vaccination on diagnosis (VoD) with gonorrhoea; or vaccination according to risk (VaR), offered to patients diagnosed with gonorrhoea plus individuals who test negative but report having more than five sexual partners per year. For the primary analysis, vaccine impact was assessed relative to no vaccination in a conservative baseline scenario wherein time-varying behavioural parameters (sexual risk behaviour and screening rates) stabilise. To calculate the value of vaccination per dose administered, the value of vaccination was calculated by summing the averted costs of testing and treatment, and the monetary value of quality-adjusted life-year (QALY) gains with a QALY valued at £20 000. Costs were in 2018–19 GB£, and both costs and QALYs were discounted at 3·5% per year. We analysed the effects of varying vaccine uptake (0·5, 1, or 2 times HPV vaccine uptake by MSM in sexual health clinics in England), vaccine efficacy (1–100%) and duration of protection (1–20 years), and the time-horizon considered (10 years and 20 years). In addition, we calculated incremental cost-effectiveness ratios for the use of 4CMenB using assumed vaccine prices.

**Findings:**

VbE has little impact on gonorrhoea diagnoses, with only 1·7% of MSM vaccinated per year. VoA has the largest impact but requires more vaccine doses than any other strategy, whereas VoD has a moderate impact but requires many fewer doses than VoA. VaR has almost the same impact as VoA but with fewer doses administered than VoA. VaR is the most cost-effective strategy for vaccines of moderate efficacy or duration of protection (or both), although VoD is more cost-effective for very protective and long-lasting vaccines. Even under conservative assumptions (efficacy equivalent to that of MeNZB and protection lasting for 18 months after two-dose primary vaccination and 36 months after single-dose booster vaccination), 4CMenB administered under VaR would likely be cost-saving at its current National Health Service price, averting an estimated mean 110 200 cases (95% credible interval 36 500–223 600), gaining a mean 100·3 QALYs (31·0–215·8), and saving a mean £7·9 million (0·0–20·5) over 10 years. A hypothetical gonorrhoea vaccine's value is increased more by improving its efficacy than its duration of protection—eg, 30% protection lasting 2 years has a median value of £48 (22–85) per dose over 10 years; doubling efficacy increases the value to £102 (53–144) whereas doubling the duration of protection increases it to £72 (34–120).

**Interpretation:**

We recommend that vaccination of MSM against gonorrhoea according to risk in sexual health clinics in England with the 4CMenB vaccine be considered. Development of gonorrhoea-specific vaccines should prioritise maximising efficacy over duration of protection.

**Funding:**

Medical Research Council (UK), National Institute for Health Research (UK).

## Introduction

Gonorrhoea diagnoses have been rising internationally,^1^ and have increased in England for a decade, reaching the highest number ever recorded in 2019.^2^ There is mounting concern about antimicrobial resistance limiting treatment options, and WHO has classified *Neisseria gonorrhoeae* (gonococcus) as a priority pathogen.^3^, ^4^ Although there are currently no gonorrhoea-specific vaccines available, MeNZB, a vaccine designed against *Neisseria meningitidis* (meningococcus) serogroup B bacteria, was estimated in a retrospective case-control study to offer protection of 31% (95% CI 21–39) against gonorrhoea.^5^ Bioinformatic analysis has suggested that the related four-component serogroup B meningococcal (4CMenB) vaccine could be more protective against gonorrhoea than MeNZB.^6^ Trials of 4CMenB against gonorrhoea are underway and candidate gonorrhoea-specific vaccines are in development.^1^, ^7^ Modelling has shown that even a partially protective vaccine could substantially reduce gonorrhoea incidence,^8^, ^9^, ^10^ and Ladhani and colleagues^11^ have advocated “serious consideration for wider use [of 4CMenB] ... at least until more targeted gonococcal-specific vaccines become available”, but important questions remain as to whether a vaccination programme would be cost-effective.^1^, ^12^

**Research in context**

**Evidence before this study**

To identify papers modelling the impact or cost-effectiveness of gonorrhoea vaccination, we searched PubMed in English on Sept 13, 2021, using the terms “gonorrh*” AND “vaccin*” AND “model*” AND (“math*” OR “transm*” OR “health econ*” OR “cost effective*” OR “cost utility”) with no date restrictions. We found four relevant papers, of which three reported transmission-dynamic modelling without health-economic analysis, and one reported health-economic analysis which did not use a transmission-dynamic model. Studies to date have looked at a relatively small number of example scenarios regarding vaccine properties, targeting strategies, and uptake.

**Added value of this study**

To our knowledge this is the first health-economic analysis of gonorrhoea vaccination that accounts for its impact on future rates of infection. Our model shows that offering vaccination against gonorrhoea to all men who have sex with men (MSM) in England attending sexual health clinics (vaccination on attendance [VoA]) has the fastest and largest impact but requires a large amount of vaccine doses and therefore is the least cost-effective strategy we considered. Offering vaccination just to those diagnosed with gonorrhoea (vaccination on diagnosis [VoD]) is much more cost-effective but has a smaller impact. We tested a novel strategy called vaccination according to risk (VaR), with risk indicated by having gonorrhoea infection or reporting a high number of sexual partners even if uninfected. Generally, VaR has almost the same impact as VoA, but with many fewer vaccine doses administered. VaR is the most cost-effective strategy for vaccines of moderate efficacy or duration of protection (or both), although VoD is more cost-effective for very protective and long-lasting vaccines. Vaccination of adolescents in schools with a vaccine protective against gonorrhoea (which would be offered to all adolescents, irrespective of sexual orientation, to protect against meningitis or gonorrhoea or both) has only a small effect on the impact and health-economic value of vaccinating MSM in sexual health clinics, because its age-restricted eligibility means it contributes little to building up coverage (only 1·7% of the MSM population vaccinated annually). The value of vaccination is relatively insensitive to uptake and future epidemic trajectories, and moderately sensitive to increasing the time-horizon from 10 years to 20 years. The value of a gonorrhoea vaccine is increased more by improving efficacy than duration of protection.

**Implications of all the available evidence**

4CMenB would likely be cost-saving in use against gonorrhoea in MSM in England at its current National Health Service price for use against infant meningitis, even under the conservative assumptions that its protection against gonorrhoea is only the same as MeNZB (31% [95% CI 21–39]), and that it protects adults for only the same duration as 4CMenB protects infants against *Neisseria meningitidis* serogroup B (ie, for 18 months after primary vaccination and 36 months after booster vaccination). Under these conditions, VaR would avert an estimated mean 110 200 (95% credible interval 36 500–223 600) cases, gain 100·3 quality-adjusted life-years (QALYs; 31·0–215·8), and save £7·9 million (0·0–20·5) over 10 years, while VoD would avert 43 900 (21 600–74 700) cases, gain 40·1 QALYs (18·4–75·1), and save £2·2 million (–0·7 to 6·3). However, VaR requires enquiring about sexual behaviour, which might be operationally challenging due to the sensitivity of the subject matter, and so a pilot study of its feasibility is recommended.

To assess the public health impact and cost-effectiveness of vaccination against gonorrhoea in men who have sex with men (MSM) in England, we used an integrated transmission-dynamic health-economic model calibrated using a Bayesian evidence-synthesis framework. MSM have a high burden of gonorrhoea internationally^1^, ^12^ and in England have the highest per-capita rate of infection, accounting for almost half of all cases.^2^ To inform decisions about whether and how to implement a vaccination programme, and the specification of preferred product characteristics, we compared alternative realistic approaches to targeting vaccination in sexual health clinics according to risk profile (with and without adolescent vaccination in schools) and examined how vaccine programme impact and cost-effectiveness depend on combinations of vaccine uptake, efficacy, and duration of protection; future epidemic trajectories; and the time-horizon considered. Finally, we assessed the impact and cost-effectiveness of 4CMenB as a gonorrhoea vaccine, using a range of parameter values to encompass the expected estimates of efficacy from trials that are underway.

## Methods

### Model structure and calibration

**Figure 1:**
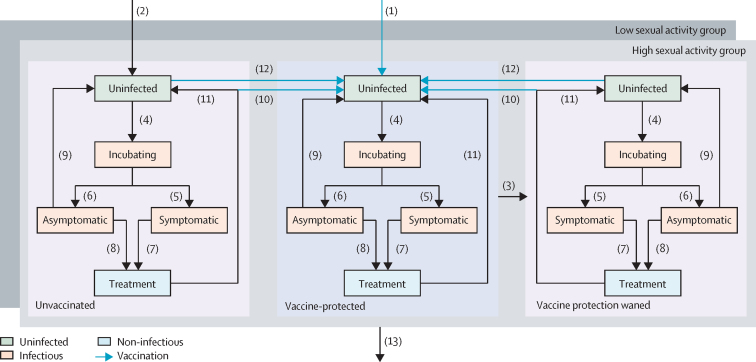
Model-structure diagram of the epidemiology of gonorrhoea and vaccination The population is divided into compartments representing different states of infection, with changes of state occurring through various processes. Individuals entering the sexually active population are uninfected, with those who receive adolescent vaccination (under the vaccination-before-entry strategy) entering the vaccine-protected stratum (1) and the remainder entering the unvaccinated stratum (2). When vaccine protection wanes, individuals are no longer protected and move from the relevant compartment in the vaccine-protected stratum to the corresponding compartment in the vaccine protection waned stratum (3). Individuals who become infected (4) pass through an incubating state, before either developing symptoms (5) and entering the symptomatic infection state, or remaining asymptomatic (6) and entering the asymptomatic infection state. Symptomatic individuals seek treatment (7) and enter the treatment state. Asymptomatic infections can be identified through screening followed by treatment (8), with individuals entering the treatment state, or there can be natural recovery (9), returning individuals to the uninfected state. All treated infections are cured (10, 11), with individuals who are vaccinated (under the vaccination-on-diagnosis or vaccination-on-attendance strategies) entering the vaccine-protected stratum (10) and those who are not vaccinated remaining in their current stratum (11)—infection does not confer natural immunity and recovered individuals are as susceptible as those never infected. Uninfected individuals who are vaccinated when screened (under the vaccination-on-attendance strategy) enter the uninfected state of the vaccine-protected stratum (12). Individuals leave the sexually active population through ageing from any state (13). Note that there are separate sets of compartments for groups of individuals with low sexual activity (darker grey layer) and high sexual activity (lighter grey layer), which have identical arrangements of compartments and vaccination-status strata; for clarity only flows in and out of the upper layer (the high sexual activity group) are shown.

We developed a deterministic transmission-dynamic compartmental model of gonorrhoea, building on our previous work,^10^, ^13^ to simulate future trajectories of the epidemic in MSM in England under different vaccination programmes (figure 1). The model incorporates care-seeking prompted by symptoms and identification of asymptomatic infection through screening, as well as natural recovery of untreated infection. Heterogeneity in sexual behaviour is represented by groups of low and high sexual activity, with the latter having higher rates of sexual partner change and attendance at sexual health clinics for screening.

Epidemiological parameters were estimated using a Bayesian evidence synthesis framework, incorporating prior information from the literature and calibrating to three time-series from 2010 to 2019: annual numbers of gonorrhoea tests and diagnoses from the Genitourinary Medicine Clinic Activity Dataset,^2^ and the proportion of diagnosed infections that were symptomatic from the Gonococcal Resistance to Antimicrobials Surveillance Programme.^14^ We used Markov Chain Monte Carlo methods to obtain a sample from the joint posterior distribution of the model parameters given the observed data, retaining 1000 parameter sets to ensure uncertainty in parameter estimates was propagated throughout the simulation analyses.

The model captures temporal trends of increasing sexual risk behaviour (such as those described by MacGregor and colleagues)^15^ and rates of screening.^2^ We considered two alternative future scenarios: a lower-bound (used in the Article) in which the inferred trends in the time-varying behavioural parameters stabilise, and an upper-bound (presented in the appendix) in which the trends continue until the end of the modelled period (ie, 2042). These two baseline scenarios do not include vaccination.

### Vaccination scenarios

We simulated the introduction of vaccination against gonorrhoea in 2022, using vaccines varying in efficacy (protection against acquiring infection 1–100%) and duration of protection (1–20 years), with four different targeting strategies (table 1). We assessed the impact of vaccinating adolescents in schools before they become sexually active (vaccination before entry [VbE]), and vaccination of MSM in sexual health clinics, comparing vaccination on diagnosis (VoD) with gonorrhoea versus vaccination on attendance (VoA), including for individuals not diagnosed with gonorrhoea. We also considered vaccination according to risk (VaR), with future risk being indicated by current infection with gonorrhoea or by patient-reported high numbers of sexual partners (more than five per year); operationally, this approach is a hybrid of VoD for the low-activity group and VoA for the high-activity group.

**Table 1:** Summary of vaccine-targeting strategies considered

	**Eligibility**	**Number of people eligible per year**	**Average risk of eligible people**	**Explanation**
Vaccination before entry (VbE)	Adolescents before they become sexually active, with only those in the relevant age-cohort each year being eligible; vaccination would be offered to all adolescents, irrespective of sexual orientation, and would be done to protect against meningitis or gonorrhoea, or both	Small, remaining constant	Same as the population	No targeting by future risk; this strategy with a vaccine that is protective against gonorrhoea would have some beneficial effect on gonorrhoea in MSM (analysed in this study) as well as some beneficial effect on gonorrhoea in other population groups (outside the scope of our analysis)
Vaccination on diagnosis (VoD)	MSM diagnosed with gonorrhoea in sexual health clinics, both through seeking care for symptomatic infection and through attending for screening (testing in the absence of symptoms)	Small, declining slightly as cases are averted	Much higher than the population average	Individuals with higher-risk behaviour are more likely to become infected and to then have the infection diagnosed, either due to symptomatic care-seeking or through screening
Vaccination according to risk (VaR)	MSM diagnosed with gonorrhoea in sexual health clinics, plus those MSM attending sexual health clinics for screening who report high-risk sexual behaviour	Moderate, declining to a small number as cases are averted	Highest of all	Includes all individuals eligible under VoD, plus those with high-risk behaviour who are not infected at the time of attendance
Vaccination on attendance (VoA)	MSM attending sexual health clinics, both those seeking care for symptomatic infection and those attending for screening, with vaccination offered irrespective of gonorrhoea infection status	Large, declining to a moderate number as cases are averted	Higher than the population average	Individuals with higher-risk behaviour are more likely to attend clinics for screening than lower-risk individuals, and are more likely to become infected and attend clinics to seek care for symptomatic infection

MSM=men who have sex with men.

Primary vaccination was assumed to require two doses, with re-vaccination after waning requiring a single booster dose to restore protection. We assumed 86·7% uptake of VbE, as in the adolescent MenACWY vaccination programme.^16^ We assumed the same uptake in sexual health clinics as for HPV vaccination of MSM (ie, 33·0% [95% CI 32·7–33·3]),^17^ which we also halved (16·5% [16·3–16·7], low uptake) and doubled (66·0% [65·4–66·6], high uptake) in scenario analyses.

### Health economic analysis

We conducted a health economic analysis taking the perspective of sexual health clinics in the UK National Health Service (NHS) and using a 3·5% annual discount rate for both costs (2018–19 GB£) and units of health (quality-adjusted life-years [QALYs]).^18^ We calculated the monetary benefit of vaccination by summing the averted costs of testing and treatment^19^ and the monetary value of averted QALY losses,^20^ with a QALY valued at £20 000 (or £30 000 in sensitivity analysis), as is standard in the UK, over 10-year and 20-year time-horizons. Each sexual health clinic vaccination strategy was compared against the following: (1) the lower-bound baseline (assuming behavioural trends stabilise) without vaccination, which was used for all results in the main paper; (2) the upper-bound baseline (assuming behavioural trends continue) without vaccination; (3) as in (1) but with VbE; and (4) as in (2) but with VbE. The value of vaccination per dose administered in sexual health clinics (which incur no cost for VbE in schools) was calculated by dividing the monetary benefit of vaccination by the discounted number of vaccine doses administered in sexual health clinics; if the cost of vaccination was less than or equal to this value, then vaccination was considered to be cost-effective.

### Impact and cost-effectiveness of 4CMenB

We assessed the value per dose of using 4CMenB against gonorrhoea in MSM in England, and we calculated incremental cost-effectiveness ratios (incremental net costs divided by incremental net QALYs gained) using assumed vaccine prices. 4CMenB is currently used by the NHS to protect infants against serogroup B meningococcal disease and, although the price paid is confidential, it has been estimated that the vaccine would be cost-effective for this use at £8 (inflation-adjusted) per dose (excluding administration cost).^21^ The efficacy of 4CMenB against gonorrhoea has not been established, but it is expected to be more protective than MeNZB,^6^ which has been estimated at 31% (95% CI 21–39).^5^ We considered scenarios where 4CMenB is 1, 1·5, 2, or 2·5 times as protective as MeNZB against gonorrhoea infection. Whereas for hypothetical vaccines we used precise values for efficacy, which we varied deterministically, for MeNZB we incorporated uncertainty in the estimated efficacy by sampling values probabilistically.

The Joint Committee on Vaccination and Immunisation (JCVI) estimates that 4CMenB protects infants against serogroup B meningococcal disease for 18 months after two-dose primary vaccination and 36 months after single-dose booster vaccination.^22^ We therefore adapted our model to allow for different durations of protection after primary vaccination and re-vaccination. We considered scenarios in which protection against gonorrhoea lasts 1, 1·5, or 2 times the JCVI estimate. Additionally, as protection lasting 4 years and even 7·5 years has been suggested for adolescents and young adults,^23^ we also considered these durations (for both primary vaccination and re-vaccination).

Full details of the model and its parameterisation are provided in the appendix (pp 2–21). All results incorporate uncertainty in epidemiological parameters, and health-economic parameters where relevant. Reported results are medians (except for incremental costs and QALYs, for which means are reported) and 95% credible intervals (CrIs).

### Role of the funding source

The sponsors of the study had no role in study design, data collection, data analysis, data interpretation, or writing of the report.

## Results

Our calibrated model successfully captures the temporal trends of increasing tests, increasing diagnoses, and the declining proportion of diagnoses that are symptomatic (appendix p 8).

**Figure 2:**
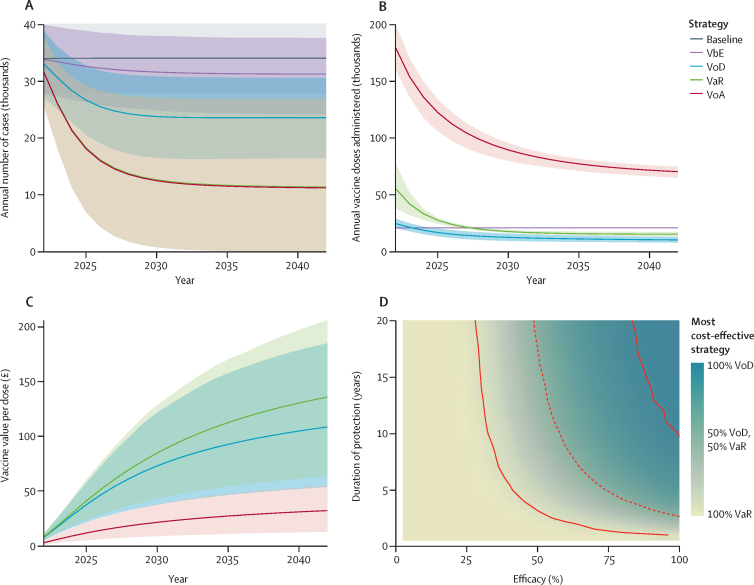
Simulations of gonorrhoea transmission over time in MSM in England, under different vaccination strategies Simulations are based on use of a vaccine providing 40% protection for 4 years, with vaccine uptake at the level of HPV vaccine uptake by MSM in sexual health clinics in England (ie, 33·0% [95% CI 32·7–33·3]). (A) Annual gonorrhoea diagnoses (note that the lines for the VaR and VoA strategies almost overlap). (B) Annual vaccine doses administered. (C) Cumulative value of vaccination per dose administered in sexual health clinics; note that there is no value calculated for the VbE strategy because we conducted the analysis taking the perspective of sexual health clinics and VbE is not provided by sexual health clinics. For panels A–C, lines represent medians and shaded regions represent 95% credible intervals. Note that panels A and B show undiscounted numbers while panel C shows discounted £ values. (D) Probability that each strategy is the most cost-effective over 20 years for vaccines ranging in efficacy (1–100%) and duration of protection (1–20 years); in all cases, either VoD or VaR is the most cost-effective strategy, and the dashed contour line shows where the two strategies have equal probability of being the most cost-effective, while the solid contour lines show where either the VoD strategy (upper right) or the VaR strategy (lower left) has a 95% probability of being the most cost-effective. MSM=men who have sex with men. VbE=vaccination before entry. VoD=vaccination on diagnosis. VaR=vaccination according to risk. VoA=vaccination on attendance.

Figure 2A–C shows the temporal effects of different vaccine-targeting strategies compared with the lower-bound baseline (which assumes behavioural trends stabilise) using a hypothetical vaccine providing 40% protection for 4 years, with ranges of uncertainty arising due to uncertainty in epidemiological parameters. For all strategies, the value of vaccination increases over time (figure 2C) due to the accumulation of cases averted. The VbE strategy has only a modest impact, with 18 700 cases (95% CrI 9700–30 300) averted over 10 years (figure 2A) because the age-restricted eligibility means that relatively few vaccine doses are administered despite the high uptake (figure 2B), with only 10 400 (1·7%) of 600 000 MSM vaccinated annually. The annual number of vaccine doses administered is constant for VbE, reflecting rates of entry into the population, but decreases under the other strategies as coverage accumulates (reducing the number of eligible, unvaccinated people) and as the number of gonorrhoea cases occurring declines because of averted transmission (reducing clinic attendances and diagnoses at which vaccination is offered; figure 2B, table 2). The strategy with the broadest eligibility criteria, VoA, has the largest impact (182 200 cases [95% CrI 77 300–296 500] averted over 10 years; figure 2A, table 2), but at the cost of using at least 5 times more vaccine doses than any other strategy (figure 2B, table 2). Restricting eligibility to only those diagnosed with gonorrhoea (VoD) reduces the impact of vaccination (85 800 cases [49 300–131 000] averted over 10 years; figure 2A, table 2), but requires around 85% fewer vaccine doses than VoA (figure 2B, table 2) and is more cost-effective than VoA (figure 2C, table 2) because it averts more cases per dose. VoD is a pragmatic strategy for targeting vaccination towards those at high risk, with gonorrhoea diagnosis being an indicator of risk of future infection.^2^ Extending eligibility to patients without gonorrhoea but reporting high numbers of partners (the VaR strategy) increases impact to a level very similar to VoA, averting 181 000 cases (75 600–295 700) over 10 years (figure 2A, table 2), but requires many fewer vaccine doses (figure 2B, table 2), and is the most cost-effective strategy for this vaccine (figure 2C, table 2).

**Table 2:** Estimated impact of gonorrhoea vaccination strategies among men who have sex with men when using a vaccine providing 40% protection for 4 years

	**Number of vaccine doses administered, thousands**			**Number of gonorrhoea cases averted over 10 years, thousands**	Value*per dose, £
	Year 1	Year 10	Years 1–10 total		
Vaccination on diagnosis	24·6 (20·3–28·8)	11·7 (8·8–14·8)	172·9 (136·4–208·6)	85·8 (49·3–131·0)	80 (41–138)
Vaccination according to risk	54·4 (38·3–78·0)	16·4 (15·2–18·0)	291·4 (249·6–333·7)	181·0 (75·6–295·7)	99 (48–147)
Vaccination on attendance	180·2 (160·6–200·3)	83·6 (75·6–88·3)	1264·4 (1119·7–1370·2)	182·2 (77·3–296·5)	23 (9–42)

Results are median (95% credible interval). Estimates presented are relative to the lower-bound baseline (in which there is no vaccination and trends in time-varying behavioural parameters stabilise). QALY=quality-adjusted life-year.

* The value of vaccination per dose was calculated by summing the averted costs of testing and treatment, and the monetary value of averted QALY losses with a QALY valued at £20 000, then dividing this monetary benefit of vaccination by the number of vaccine doses administered in sexual health clinics, considering a 10-year period with discounting at 3·5% per year.

We systematically repeated the analysis for vaccines of varying efficacy (1–100%) and duration of protection (1–20 years). For vaccines with lower than 25% efficacy, VaR is the most cost-effective strategy, with more than 95% probability; however, as efficacy and duration of protection increase, the probability that VoD is the most cost-effective strategy increases (figure 2D).

The alternative upper-bound baseline, which assumes behavioural trends continue, has a greater number of gonorrhoea cases and therefore a greater number averted by vaccination compared with the lower-bound baseline, but it also has a greater number of vaccine doses administered (except under VbE), so the cost-effectiveness of vaccination in sexual health clinics is increased only modestly (around 20%), and our conclusions remain qualitatively the same (appendix pp 25–26). Because VbE has only a marginal effect, including when combined with vaccination in sexual health clinics (appendix pp 24, 26), we did not consider it further.

**Figure 3:**
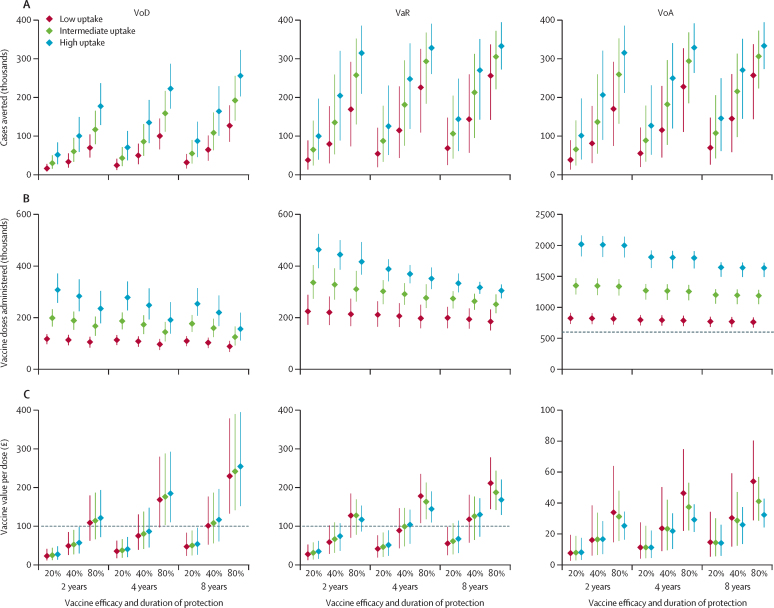
Effects of vaccine uptake, efficacy, and duration of protection on the impact and cost-effectiveness of three vaccination strategies against gonorrhoea in men who have sex with men in England over 10 years (A) Total cases averted by vaccination. (B) Total number of vaccine doses administered. (C) Value of vaccination per dose administered. Columns of panels correspond to the three targeting strategies assessed (VoD, VaR, and VoA). Three levels of efficacy (20%, 40%, or 80%) and three durations of protection (2, 4, or 8 years) are represented along the x-axis. Different coloured bars correspond to three different levels of uptake: the low-uptake scenario (16·5% [95% CI 16·3–16·7]), which is half the level of HPV vaccine uptake by MSM in sexual health clinics in England (ie, 33·0% [32·7–33·3], labelled intermediate on the graph), and one-quarter the level of the high-uptake scenario (66·0% [65·4–66·6]). Points show medians and bars show 95% credible intervals. Note that panels A and B show undiscounted numbers while panel C shows discounted £ values. Also note that in panel B, the dashed line in the VoA graph shows the upper limit of the y-axis scales of the VoD and VaR graphs, and in panel C, the dashed lines in the VoD and VaR graphs show the upper limit of the y-axis scale of the VoA graph. VoD=vaccination on diagnosis. VaR=vaccination according to risk. VoA=vaccination on attendance.

We examined nine hypothetical vaccines varying in efficacy (20%, 40%, or 80%) and duration of protection (2, 4, or 8 years) for each targeting strategy, with three levels of uptake (figure 3). The higher the efficacy and duration of protection the greater the impact of vaccination (figure 3A), and the fewer vaccine doses administered (figure 3B)—due to the reduction in symptomatic infection prompting attendance at sexual health clinics—and therefore the greater the cost-effectiveness of vaccination (figure 3C). The number of cases averted ranges from 30 100 (95% CrI 15 600–50 100; for a vaccine with 20% efficacy and a 2-year duration of protection) to 192 200 (140 500–255 600; for 80% efficacy with an 8-year duration of protection) for VoD, from 64 900 (23 900–140 200) to 305 300 (221 100–372 300) for VaR, and from 65 800 (24 400–140 300) to 306 200 (223 200–372 500) for VoA, with uptake of 33·0% (95% CI 32·7–33·3; figure 3A). The number of vaccine doses administered ranges from 0·20 million (0·17–0·23) to 0·12 million (0·09–0·17) for VoD, from 0·34 million (0·27–0·40) to 0·25 million (0·22–0·29) for VaR, and from 1·35 million (1·21–1·47) to 1·19 million (1·05–1·28) for VoA (figure 3B). The value per dose ranges from £25 (12–44) to £242 (142–390) for VoD, from £31 (14–58) to £187 (142–243) for VaR, and from £8 (3–19) to £41 (29–57) for VoA) (figure 3C).

Increasing uptake of vaccination increases the vaccination programme's impact (figure 3A) and cost (figure 3B). Increasing uptake from low to high of a vaccine with 40% efficacy lasting 4 years increases the numbers of cases averted from 49 800 (27 600–80 300) to 135 100 (81 800–193 400) for VoD, from 114 900 (43 700–228 400) to 247 800 (119 000–340 000) for VaR, and from 115 600 (45 000–229 300) to 249 700 (120 000–340 400) for VoA, with numbers of doses administered increasing from 0·11 million (0·09–0·13) to 0·25 million (0·19–0·31) for VoD, from 0·21 million (0·16–0·26) to 0·37 million (0·33–0·40) for VaR, and from 0·79 million (0·70–0·87) to 1·80 million (1·63–1·91) for VoA. For VoD and VaR, the value per dose increases with uptake except for the latter with high-efficacy vaccines. For VoA, the value per dose decreases with increasing uptake. However, for a given vaccine and targeting strategy, halving or doubling uptake makes a relatively small difference to the value per dose (figure 3C).

Increasing the time-horizon from 10 years to 20 years or using the alternative upper-bound baseline does not alter our conclusions (appendix pp 28–31). The main determinants of vaccination programme cost-effectiveness are the vaccine efficacy and duration of protection and the targeting strategy, so we used only the estimated 33·0% (95% CI 32·7–33·3) in further analyses.

**Figure 4:**
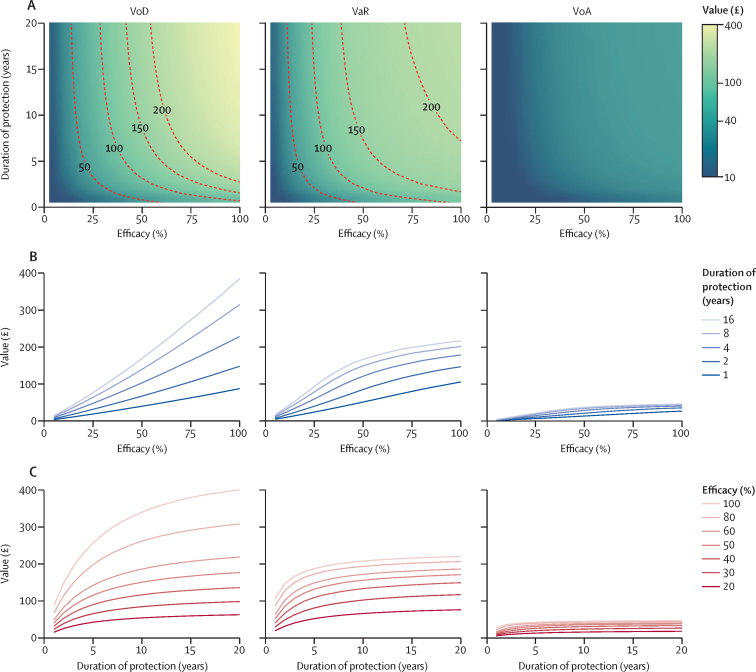
Value of vaccination against gonorrhoea under three targeting strategies among men who have sex with men in England over 10 years (A) Heatmaps of median value per dose administered of vaccines providing 1–100% protection against infection for 1–20 years, with contour lines showing values of £50, £100, £150, and £200. (B) Impact of duration of protection on the median value per dose administered of vaccines with a range of efficacies. (C) Impact of efficacy on the median value per dose administered of vaccines with a range of durations of protection. Columns of panels correspond to the three targeting strategies assessed (VoD, VaR, and VoA). The lines plotted in panels B and C are transects through the heatmaps in panel A, with value represented on the vertical axis rather than by colour. Vaccine uptake is 33·0% (95% CI 32·7–33·3). VoD=vaccination on diagnosis. VaR=vaccination according to risk. VoA=vaccination on attendance.

A highly efficacious vaccine with long-lasting protection could be worth more than £220 per dose under VoD or VaR, although even 100% protection lasting 20 years would be worth only £46 (95% CrI 34–62) per dose under VoA (figure 4A). The value of vaccination increases linearly with efficacy under VoD, but shows diminishing returns at efficacies more than 50% under VaR and VoA (figure 4B). Improving the duration of protection from 1 year increases value steeply at first, but quickly shows diminishing returns (figure 4C). A similar pattern is seen over 20 years (appendix p 34). Improving vaccine efficacy is likely to increase value more than improving the duration of protection by an equivalent factor. For example, under VaR, for a vaccine providing 30% protection for 2 years (with a value of £48 [22–85]), doubling its efficacy would increase its value to £102 (53–144), whereas doubling its duration of protection would increase its value to £72 (34–120).

**Figure 5:**
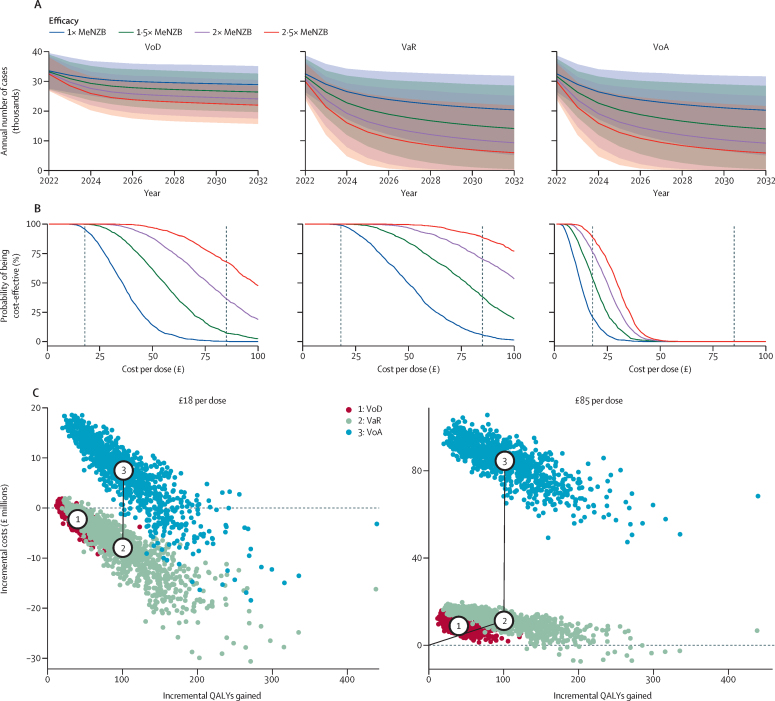
Impact of 4CMenB vaccination over 10 years, how the cost per dose affects the probability of vaccination being cost-effective, and cost-effectiveness efficiency frontiers comparing targeting strategies (A) Cases of gonorrhoea in men who have sex with men in England following introduction of vaccination in 2022 under different targeting strategies (VoD, VaR, and VoA) using the 4CMenB vaccine, assuming the vaccine is 1, 1·5, 2, or 2·5 times as protective as the MeNZB vaccine, and has durations of protection after primary vaccination and re-vaccination as estimated by the Joint Committee on Vaccination and Immunisation for protection of infants against serogroup B meningococcal disease (ie, 18 months after two-dose primary vaccination and 36 months after re-vaccination). Lines represent medians and shaded regions represent 95% credible intervals. (B) Probability that vaccination is cost-effective (ie, its value exceeds its cost) with the same vaccine efficacies and durations of protection as in panel A. Vertical dashed lines show two alternative costs per dose of 4CMenB administered: £18, corresponding to the estimated National Health Service price of £8 per dose plus the £10 administration cost; or £85, corresponding to the current UK list price of £75 plus the £10 administration cost. (C) Cost-effectiveness efficiency frontiers comparing the three targeting strategies if 4CMenB is as protective as MeNZB, the durations of protection are the same as in panels A and B, and the cost per dose administered is £18 or £85. The black line shows the frontier, the numbered circles show the mean incremental costs and QALYs for each strategy, and the individual points show the uncertainty represented by the 1000 sets of sampled epidemiological and health-economic parameters. In all panels vaccine uptake is 33·0% (95% CI 32·7–33·3). Note that panel A shows undiscounted numbers while panel C shows discounted values. QALY=quality-adjusted life-year. VoD=vaccination on diagnosis. VaR=vaccination according to risk. VoA=vaccination on attendance.

4CMenB could rapidly reduce cases of gonorrhoea in MSM in England under VaR or VoA (figure 5A). If 4CMenB protects for 18 months after primary vaccination and 36 months after repeat vaccination, and is as protective as MeNZB, then under the VaR strategy (the most cost-effective strategy) it would need to cost less than £50 per dose (including the £10 cost of administration by clinics) to have more than 50% probability of being cost-effective over a 10-year time-horizon (figure 5B). However, if the vaccine is 1·5, 2, or 2·5 times as protective as MeNZB then its estimated value is £77 (95% CrI 33–128), £102 (49–150), or £123 (65–168) per dose, respectively. Under the VoD strategy, the estimated value of 4CMenB is £36 (17–65), £56 (26–99), £76 (37–135), £98 (48–168) per dose for protection 1, 1·5, 2, or 2·5 times that of MeNZB, respectively. Under the VoA strategy, the corresponding values are £12 (4–28), £19 (7–37), £25 (10–43), and £30 (13–46). Comparing targeting strategies incrementally, VaR is the most cost-effective if the cost per vaccine dose administered is £18. At this cost, if 4CMenB protects for 18 months after primary vaccination and 36 months after repeat vaccination, and is as protective as MeNZB, then VaR averts an estimated median 104 000 cases (mean 110 200 [95% CrI 36 500–223 600]), gains an estimated mean 100·3 QALYs (31·0–215·8), and saves an estimated mean £7·9 million (0·0–20·5) over 10 years compared with no vaccination, while VoD averts an estimated median 42 800 cases (mean 43 900 [95% CrI 21 600–74 700]), gains an estimated mean 40·1 QALYs (18·4–75·1), and saves an estimated mean £2·2 million (–0·7 to 6·3) versus no vaccination; hence, VaR dominates VoD (ie, VaR is cheaper and gains more QALYs; figure 5C; appendix p 52). If vaccination costs £85 per dose administered, then no strategy is cost-effective with a willingness-to-pay of £20 000 per QALY or £30 000 per QALY (figure 5C; appendix p 52).

Assuming greater efficacy or duration of protection, increasing the time-horizon from 10 years to 20 years, using the alternative upper-bound baseline, or valuing a QALY at £30 000 all increase the estimated value of 4CMenB (appendix pp 38–41) and the cost-effectiveness of vaccination (appendix pp 52–55). The appendix contains tables of the estimated value of vaccination under the three targeting strategies for vaccines with a range of efficacies and durations of protection, considering 10-year versus 20-year time-horizons, and using lower-bound and upper-bound baselines (appendix pp 42–45); costs corresponding to a 90% probability of being cost-effective with a QALY valued at £20 000 versus £30 000 are also presented (appendix pp 46–49).

The estimated value of vaccination is largely robust to uncertainty in the health-economic parameters (appendix p 56). It is most sensitive to the unit cost of treatment, which affects the value by around 20%, whereas other parameters have effects below 10%.

## Discussion

Our modelling analysis has several key findings. First, despite the likely high uptake of an adolescent vaccination programme, VbE has only a modest impact on gonorrhoea incidence in MSM because of a slow accrual of coverage, and it is therefore ineffective by itself and makes little difference to the impact or cost-effectiveness of vaccination offered to MSM in sexual health clinics (ie, VoD, VaR, or VoA). Second, VoA achieves the fastest and largest impact but at high cost, whereas VoD is highly cost-effective but has much less impact. Third, pursuing a hybrid strategy with targeting informed by self-reported behaviour (VaR) could achieve a similar impact to VoA at a fraction of the cost. Although VoD is more cost-effective for very protective and long-lasting vaccines, VaR is the most cost-effective strategy for vaccines likely to be available in the near future. However, VaR requires enquiring about sexual behaviour, which might be operationally challenging due to the sensitivity of the subject matter; currently, HPV vaccination is offered in sexual health clinics to all MSM without patients being asked about sexual behaviour. Fourth, higher uptake increases impact for all strategies, but increases costs by a similar proportion, and hence makes relatively little difference to cost-effectiveness. Fifth, increasing the time-horizon from 10 years to 20 years increases cost-effectiveness by around 40%, but with new vaccine candidates in development, decision makers might prefer to consider the shorter time-horizon. Sixth, the cost-effectiveness of vaccination is greater if behavioural trends continue rather than stabilise, although the magnitude is modest (around 20% greater over 10 years, and around 35% over 20 years) considering the large increase in incidence. Seventh, improving efficacy increases a vaccine's value more than improving duration of protection.

As a new vaccine will take years to develop, the immediate question for policy makers is whether 4CMenB should be used against gonorrhoea. Its efficacy is expected to exceed that of MeNZB,^6^ but will not be known definitively until trials report in the next few years.^7^, ^24^ A recent observational study estimated an effectiveness of 40% (95% CI 23 to 53; around 1·3 times that of MeNZB),^25^ while the MenGo trial^7^ is powered to detect efficacy of 66·7% (around 2·15 times that of MeNZB). We found that if 4CMenB is as protective against gonorrhoea as MeNZB (31% [95% CI 21 to 39])^5^ for the duration that JCVI estimates 4CMenB protects infants against serogroup B meningococcal disease (ie, 18 months after primary vaccination and 36 months after booster vaccination),^22^ then using 4CMenB to vaccinate MSM in sexual health clinics under the VaR strategy would save £7·9 million (0·0 to 20·5) and lead to 100·3 QALYs (31·0 to 215·8) being gained over 10 years at the estimated NHS price of £8 per dose (plus £10 administration cost), using the lower-bound baseline in which behavioural trends stabilise. However, if VaR is not feasible, then VoD would save £2·2 million (–0·7 to 6·3) and result in 40·1 QALYs (18·4 to 75·1) being gained.

A strength of our analysis is that we calibrated our transmission-dynamic model to more data sources than were used in previous studies,^10^, ^13^ and we used Bayesian methods to account for uncertainty in epidemiological parameters, which was captured using 1000 parameter sets sampled from the posterior distribution in all simulations. The model incorporates heterogeneity and changes in sexual behaviour (such as those described by MacGregor and colleagues),^15^ and reproduces observed trends in testing, diagnoses, and the proportion of diagnoses that are symptomatic. As the future trajectory of the epidemic is uncertain, we considered alternative baselines to bracket the actual trajectory: either assuming that current rising trends cease (which is probably an underestimation) or assuming that trends continue for two decades beyond the data (which is probably a substantial exaggeration). Our findings are robust to this uncertainty: although our main analysis is conservative because we use the lower-bound baseline, the much larger epidemic in the upper-bound baseline results in only a modest increase in cost-effectiveness.

Our estimation of the value of gonorrhoea vaccination is conservative for several other reasons. First, because of a lack of data at the time of the study, we assumed that the initial vaccine dose would offer no protection; however, Abara and colleagues have since reported that a single dose offers 26% (95% CI 12–37) protection,^25^ which would increase the value of vaccination. Second, as we discussed previously,^10^ we assumed that imperfect protection was leaky, with all vaccinees remaining at risk of infection (albeit reduced); if an imperfect vaccine actually provided complete protection to some vaccinees and no protection to others then the benefits of vaccination would be slightly greater.^26^ Third, the full burden of gonorrhoea in terms of disease sequelae (including epididymitis and disseminated gonorrhoea infection) and their cost to the health service have not been quantified because of a lack of reliable quantitative information for England, so we have understated the full benefits of averting infection.

Most importantly, the benefits of reducing the potential future burden of antimicrobial resistance are unknown and could be substantial. First, antibiotic stewardship will require resistance-guided therapy, using more-costly tests, and so reducing the incidence of infection will reduce testing costs. Second, treating drug-resistant infection requires more-costly drugs and increased staff time for patient management (with some cases being very expensive),^27^ while prolonged symptoms and potentially greater treatment side-effects will increase QALY losses. Third, reducing infection prevalence reduces the opportunity for emergence of drug resistance through mutation and recombination, and reducing antibiotic use reduces selective pressure for resistance.^28^ These reductions would increase the value of VaR and VoA the most because they have the greatest impact on prevalence. For a gonorrhoea-specific vaccine, larger-scale use under VoA might enable a lower vaccine price to be negotiated.

Assessing the full value of vaccination against gonorrhoea requires further work to quantify the full burden of infection as well as the expected future burden of antimicrobial resistance and how much of this can be averted.^1^, ^26^ We considered MSM, who have the highest per-capita rate of infection and highest level of antimicrobial resistance in England;^29^ analysis is also required for heterosexual populations (including subgroups such as sex workers),^1^ which should include improving estimates of the burden of infection and sequelae in women and men, and the effectiveness and cost-effectiveness of realistic vaccine-targeting strategies.^1^, ^12^, ^30^ Future development of gonorrhoea-specific vaccines should prioritise increasing efficacy over duration of protection. Meanwhile, at its current NHS price for use against infant meningitis, 4CMenB would likely be cost-saving in use against gonorrhoea in MSM in England.

**This online publication has been corrected. The corrected version first appeared at thelancet.com/infection on April 29, 2022**

## Data sharing

All data used in this study are publicly available from the cited sources.

## Declaration of interests

PJW has received payment from Pfizer for teaching of mathematical modelling of infectious disease transmission and vaccination. All other authors declare no competing interests.
