# Correction to *Lancet Infect Dis* 2022; published online April 12. https://doi.org/10.1016/S1473-3099(21)00744-1

**DOI:** 10.1016/S1473-3099(22)00280-8

**Published:** 2022-06

*Whittles LK, Didelot X, White PJ. Public health impact and cost-effectiveness of gonorrhoea vaccination: an integrated transmission-dynamic health-economic modelling analysis.* Lancet Infect Dis *2022; published online April 12. https://doi.org/10.1016/S1473-3099(21)00744-1*—In the Summary of this Article, the first sentence of the Interpretation has been amended to “We recommend that vaccination of MSM against gonorrhoea according to risk in sexual health clinics in England with the 4CMenB vaccine be considered.” This correction has been made to the online version as of April 29, 2022, and will be made to the printed version.

