# Supplementary material for: Public health impact and cost-effectiveness of gonorrhoea vaccination: an integrated transmission-dynamic health-economic modelling analysis
Source: Lancet Infect Dis. 2022 Jul;22(7):1030–41. doi: 10.1016/S1473-3099(21)00744-1 (PMC9217755; doi:10.1016/S1473-3099(21)00744-1)
Supplement: Supplementary appendix [file mmc1.pdf]

THE LANCET
Infectious Diseases

Supplementary appendix

This appendix formed part of the original submission and has been peer reviewed. We post it as supplied by the authors.

Supplement to: Whittles LK, Didelot X, White PJ. Public health impact and cost-effectiveness of gonorrhoea vaccination: an integrated transmission-dynamic health-economic modelling analysis. *Lancet Infect Dis* 2022; published online April 12. https://doi.org/10.1016/S1473-3099(21)00744-1.

Appendix to

Public health impact and cost-effectiveness of gonorrhoea vaccination: an integrated transmission-dynamic health-economic modelling analysis

Contents

1 Model details: methods and calibration **2**
1.1 Model of gonorrhoea transmission . . . 2
1.1.1 Compartmental model equations . . . 4
1.2 Model calibration . . . 4
1.2.1 Data sources . . . 4
1.2.2 Observation process . . . 4
1.2.3 Fixed model parameters . . . 5
1.2.4 Prior distributions of fitted model parameters . . . 6
1.2.5 Calibration process . . . 6
1.2.6 Results of calibration . . . 7
1.3 Model of vaccination . . . 10
1.3.1 Compartmental model equations . . . 12
1.3.2 Simulations . . . 13
1.3.3 Vaccine uptake and calculation of total doses . . . 13
1.3.4 Comparison baselines . . . 15
1.3.5 Health-economic analysis . . . 15
1.4 Model of differential waning of protection after primary vaccination vs repeat vaccination 17
1.4.1 Compartmental model equations . . . 19
1.4.2 Simulations . . . 21
1.4.3 Health-economic analysis . . . 21

2 Supplementary results **22**
2.1 Effect of vaccine targeting strategy . . . 22
2.2 Effects of vaccine efficacy, duration of protection, targeting strategy, and uptake . . . 27
2.3 Effects of vaccine efficacy and duration of protection . . . 32
2.4 Analysis of 4CMenB . . . 37
2.4.1 Value per dose . . . 37
2.4.2 Comparison of vaccine targeting strategies . . . 50
2.4.3 Sensitivity to health economic parameters . . . 56

1 Model details: methods and calibration

1.1 Model of gonorrhoea transmission

We developed a deterministic transmission-dynamic compartmental model of gonorrhoea, which divides the England MSM population by infection status, presence of symptoms, and into two groups based on level of sexual activity (Figure S1). In the model, MSM enter the population on sexual debut (α per year), with a proportion (q_L) joining the low sexual activity group, and the remainder ($q_H = 1 - q_L$) joining the high-activity group. Individuals entering the model population are uninfected (U). Acquisition of infection occurs at rate $\lambda_j(t)$ through sexual contact with a contagious individual ($C = I + A + S$). Newly-infected individuals pass through an incubation period (I) at rate σ, after which a proportion ψ develop symptoms (S) while the rest remain asymptomatic (A). Symptomatic individuals seek treatment at rate μ. Asymptomatic individuals may be diagnosed and treated through sexual health screening, at rate $\eta_j(t)$, or else recover naturally at rate ν. Individuals diagnosed with gonorrhoea are treated and recover at rate ρ, after which they are again susceptible to infection (U). Infection does not confer natural immunity and recovered individuals are equally as susceptible as those never infected.

The force of infection, i.e. the rate of acquisition of infection for an uninfected individual, in sexual activity group j, $\lambda_j(t)$, depends on the rate of partner change per year in each sexual activity group (c_j); the level of assortativity in sexual mixing between groups (ϵ, where $\epsilon = 0$ denotes proportionate mixing an $\epsilon = 1$ denotes fully assortative contact [1]); the prevalence of infectious individuals in each group ($C_j(t)/N_j(t)$); and the rate of transmission, which we allow to change linearly over time from the initial rate (β) at $t_0 =$ 01-01-2010, with an annual increase of ϕ_β. The force of infection is calculated as follows:

$$\lambda_j(t) = c_j\beta(1 + \phi_\beta(t - t_0))\left(\epsilon\frac{C_j(t)}{N_j(t)} + (1 - \epsilon)\left(\sum_{i\in\{L,H\}} \pi_i(t)\frac{C_i(t)}{N_i(t)}\right)\right) \tag{1}$$

Where $\pi_j(t) = \frac{c_j N_j(t)}{\sum_{i\in\{L,H\}} c_i N_i(t)}$ is the proportion of all partnerships in the population that involve a member of group j.

The rate of screening (i.e. testing in the absence of symptoms, which applies to asymptomatic and uninfected individuals), $\eta_j(t)$, depends on the sexual activity group. The rate of screening in both sexual activity groups changes linearly over time, increasing by ϕ_η each year from the initial rate at t_0:

$$\eta_H(t) = \eta_H(t_0)(1 + \phi_\eta(t - t_0)) \tag{2}$$

$$\eta_L(t) = \omega\eta_H(t) \tag{3}$$

Where $0 < \omega < 1$, to reflect the fact that individuals in the low-activity group seek screening less often than those in the high-activity group.

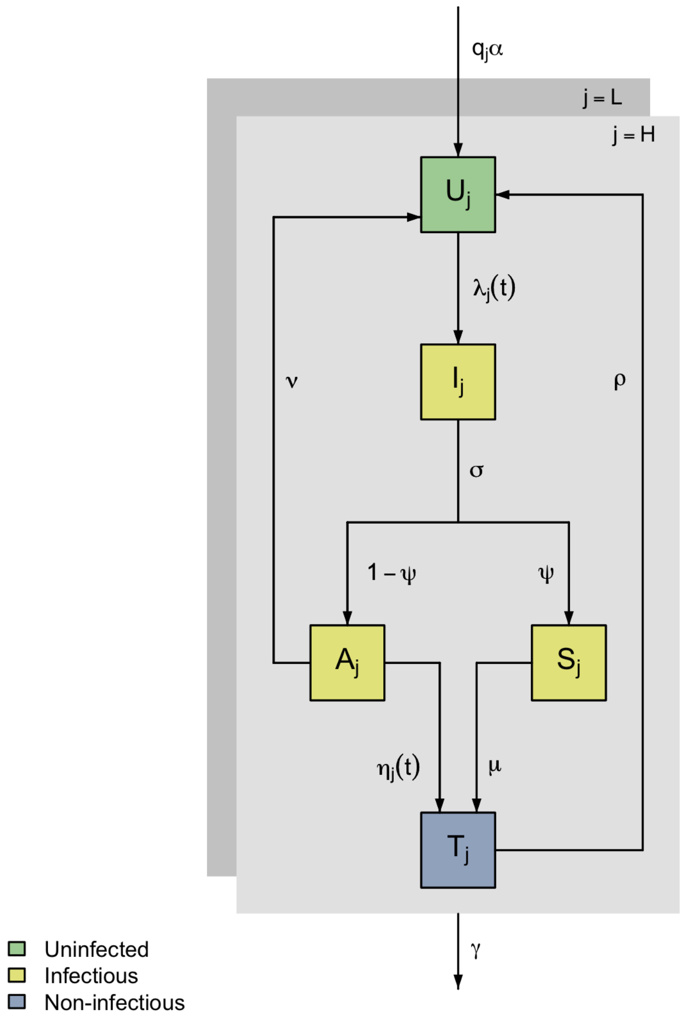

Figure S1: Model-structure diagram for the epidemiology of gonorrhoea. The population is divided into compartments representing different states of infection. Individuals entering the sexually-active population are uninfected (U_j). Individuals who become infected pass through an incubating state (I_j), before either developing symptoms (S_j), or remaining asymptomatic (A_j). Symptomatic individuals seek treatment and enter the treatment state (T_j). Asymptomatic infections can be identified through screening, with individuals entering the treatment state (T_j), or there can be natural recovery, returning individuals to the uninfected state (U_j). All treated infections are cured. Individuals leave the sexually-active population through ageing from any state. Note that there are separate sets of compartments for those in the low and high sexual activity groups ($j \in \{L, H\}$), represented by the darker- and lighter-grey layers of the diagram, respectively, which have an identical arrangement of compartments; for clarity only flows in and out of the upper layer (the high-activity group) are shown.

1.1.1 Compartmental model equations

We describe the model depicted in Figure S1 in differential equations. Each compartment is stratified by sexual activity group $j \in \{L, H\}$. Full definitions of model parameters are set out in Table S1 and Table S2.

$$\frac{dU_j(t)}{dt} = q_j\alpha - (\lambda_j(t) + \gamma)U_j(t) + \nu A_j(t) + \rho T_j(t) \tag{4}$$

$$\frac{dI_j(t)}{dt} = \lambda_j(t)U_j(t) - (\sigma + \gamma)I_j(t) \tag{5}$$

$$\frac{dA_j(t)}{dt} = (1 - \psi)\sigma I_j(t) - (\nu + \eta_j(t) + \gamma)A_j(t) \tag{6}$$

$$\frac{dS_j(t)}{dt} = \psi\sigma I_j(t) - (\mu + \gamma)S_j(t) \tag{7}$$

$$\frac{dT_j(t)}{dt} = \eta_j(t)A_j(t) + \mu S_j(t) - (\rho + \gamma)T_j(t) \tag{8}$$

1.2 Model calibration

1.2.1 Data sources

The genitourinary medicine clinic activity dataset (GUMCAD) reports annual gonorrhoea tests and diagnoses by gender and sexual orientation from all STI clinics in England[2], where the vast majority of gonorrhoea is diagnosed [3]. The Gonococcal Resistance to Antimicrobials Surveillance Programme (GRASP) is a sentinel surveillance system which reports the proportion of diagnosed infections that were symptomatic [4].

We used a Bayesian evidence synthesis framework to calibrate the model to three surveillance time-series, in the period 2010-2019:

1. The number of gonorrhoea tests in MSM recorded by GUMCAD ($Z_T(t)$);
2. The number of gonorrhoea diagnoses in MSM recorded by GUMCAD ($Z_D(t)$);
3. The proportion of gonorrhoea diagnoses recorded by GRASP that were symptomatic ($Z_S(t)/Z_R(t)$).

1.2.2 Observation process

We compared the observed annual number of gonorrhoea tests and diagnoses in the GUMCAD sample, $Z_T(t), Z_D(t)$, with those predicted by our model, $Y_T(t), Y_D(t)$, using Negative-Binomial likelihoods to allow for over-dispersion in the observation process relative to a Poisson distribution. If $X \sim \text{NegBinom}(m, \kappa)$, with mean m and shape parameter κ, then:

$$f_X(x|m,\kappa) = \frac{\Gamma(\kappa + x)}{x!\Gamma(\kappa)}\left(\frac{\kappa}{\kappa + m}\right)^{\kappa}\left(\frac{m}{\kappa + m}\right)^{x}$$

where $\Gamma(\cdot)$ is the Gamma function. Under the Negative-Binomial distribution $\text{Var}[X] = m + \frac{m^2}{\kappa}$.

The likelihoods of $Z_T(t)$ and $Z_D(t)$ were:

$$Z_T(t) \sim \text{NegBinom}(Y_T(t), \kappa_T)$$
$$Z_D(t) \sim \text{NegBinom}(Y_D(t), \kappa_D)$$

where:

$$Y_T(t) = \sum_{j \in \{L,H\}} \left(\int_t^{t+1} \eta_j(\tau)\big(U_j(\tau) + A_j(\tau)\big) + \mu S_j(\tau) d\tau \right) \tag{9}$$

$$Y_D(t) = \sum_{j \in \{L,H\}} \left(\int_t^{t+1} \rho T_j(\tau) d\tau \right) \tag{10}$$

We used the same shape parameter, $\kappa_D = \kappa_T$, for both GUMCAD data streams, which we fitted within the Bayesian framework.

We compared the observed annual number of symptomatic diagnoses in the GRASP sample $Z_S(t)$ with that predicted by our model using a Beta-Binomial likelihood. If $X \sim \text{BetaBinom}(n, p, \kappa)$, with size n, probability p and over-dispersion κ, then:

$$f_X(x|n, p, \kappa) = \binom{n}{x} \frac{B(x + a, n - x + b)}{B(a, b)}$$

where $B(\cdot, \cdot)$ is the Beta function and $a = p\left(\frac{1-\kappa}{\kappa}\right), b = \left(1 - p\right)\left(\frac{1-\kappa}{\kappa}\right)$. Under the Beta-Binomial distribution, the mean and variance of X are $\text{E}[X] = np$ and $\text{Var}[X] = np\left(1-p\right)\left(1+(n-1)\kappa\right)$ respectively.

The model-predicted probability that a diagnosis in year t was symptomatic is $\frac{Y_S(t)}{Y_S(t)+Y_A(t)}$, where $Y_S(t)$ and $Y_A(t)$ are the numbers of symptomatic and asymptomatic diagnoses in year t, respectively, calculated as follows:

$$Y_S(t) = \sum_{j \in \{L,H\}} \left(\int_t^{t+1} \mu S_j(\tau) d\tau \right) \tag{11}$$

$$Y_A(t) = \sum_{j \in \{L,H\}} \left(\int_t^{t+1} \eta_j(\tau) A_j(\tau) d\tau \right) \tag{12}$$

We modelled the number of symptomatic diagnoses in GRASP, accounting for the size of the GRASP sample, $Z_R(t)$, and fitted the shape parameter for the GRASP dataset, κ_S, within the Bayesian framework:

$$Z_S(t) \sim \text{BetaBinom}\left(Z_R(t), \frac{Y_S(t)}{Y_S(t) + Y_A(t)}, \kappa_S \right)$$

The overall likelihood of the data given the modelled trajectories produced by parameter set Θ was calculated as the product of the likelihoods of the three data streams in each year $t = 2010, \ldots, 2019$:

$$L(Z|\Theta) = \prod_{t=2010}^{2019} f_{Z_D}(z_D(t)|\Theta) f_{Z_T}(z_T(t)|\Theta) f_{Z_S}(z_S(t)|z_R(t), \Theta) \tag{13}$$

1.2.3 Fixed model parameters

We calibrated population demographics as described in [5, 6], adopting a population size of $N = 600,000$ MSM. We consider the sexually-active population aged 15-65, giving a population exit rate of $\gamma = 1/50$ per year (Table S1). Previous analysis of demographic data suggested that the MSM population size is likely to remain stable over the time-horizon considered [6]. We therefore maintained a constant population size by allowing for $\alpha = 12,000$ new population entrants each year. The proportion of MSM in each activity group (q_j), and the respective rates of changing partners (c_j) were calibrated to Natsal-3 data, based on a threshold of $\leq 5/> 5$ partners per year [6, 7]. We assume that individuals remain in their activity group throughout the modelled period, as we do not have data to inform on any movement between groups.

Table S1: Fixed model parameters: notation, definitions, source of estimates.

	Definition	Value	Source
$N(t_0)$	Initial population size of England MSM	600,000	[6]
α	Annual population entrants (at age 15)	12,000	[8]
$\frac{1}{\gamma}$	Years spent in the sexually-active population	50	Ages 15-65
q_L	Proportion of the population in group L	0·85	[7]
q_H	Proportion of the population in group H	0·15	$1 - q_L$
c_L	Annual rate of partner change in group L	0·6	[6]
c_H	Annual rate of partner change in group H	15·6	[6]

1.2.4 Prior distributions of fitted model parameters

We set priors for the model parameters that reflected existing knowledge and uncertainty about their likely range of values, as set out in Table S2. We adopted Uniform priors with a large range for parameters whose values are highly uncertain and therefore best determined from the data, namely: the level of assortativity in sexual mixing between activity groups (ϵ), the initial prevalence of asymptomatic gonorrhoea in the low and high activity groups $\left(\frac{A_L(t_0)}{N_L}, \frac{A_H(t_0)}{N_H}\right)$, the rate of per-partnership transmission (β), the annual increase in transmission risk (ϕ_β), the annual increase in the asymptomatic screening rate (ϕ_η), the probability of developing symptoms after infection (ψ), and the shape parameters relating the observation distributions (κ_D, κ_S).

The relative screening rate for MSM in group L compared to group H (ω) was assigned a LogNormal prior, calibrated to data from Natsal-3 [9]. In the study, 23 MSM who reported 0-4 partners in the last year had attended an STI clinic for any reason, while 107·9 had not (non-integer values are due to the study's sample weighting). The equivalent figures for MSM reporting five or more partners were 10·2 vs 17·1. We denoted the probability of attending an STI clinic in the last $p_j^S; j \in \{L, H\}$, and assigned corresponding Beta priors:

$$p_L^S \sim \text{Beta}(23{\cdot}0, 107{\cdot}9)$$
$$p_H^S \sim \text{Beta}(10{\cdot}2, 17{\cdot}1)$$

If the time to attending a sexual health clinic is distributed exponentially, then the ratio of the rate at which MSM in group L attend vs those in group H is:

$$\omega = \frac{\ln(1 - p_L^S)}{\ln(1 - p_H^S)}$$

Since the distribution of ω is not analytically tractable, we drew 1 million independent samples from the distributions of p_L^S, p_H^S, and fitted a LogNormal distribution to the calculated sample for ω, giving a prior distribution of $\text{logN}(-0{\cdot}87, 0{\cdot}39)$ (Table S2).

We assigned informative priors to the remaining model parameters ($\sigma, \mu, \rho, \nu, \eta_H(t_0)$) based on literature review as described in [6] (Table S2).

1.2.5 Calibration process

We used Markov chain Monte Carlo (MCMC) methods to obtain a sample from the posterior distribution of the model parameters given the observed data, via a Metropolis-Hastings sampling algorithm. At each MCMC iteration, the sampler proposes to update the joint posterior distribution of the parameters. The proposal kernel is multivariate Gaussian centred on current parameter values, with covariance structure manually tuned to facilitate efficient Markov chain mixing. For parameters with finite support, we specified reflecting boundaries for the proposal kernel to ensure proposed values remained mathematically and epidemiologically plausible, and to ensure that the proposal kernels remained symmetrical.

We ran eight independent chains of the MCMC sampler for 50,000 iterations, with the first 1,000 iterations discarded as burn-in. We assessed convergence by ensuring that the multivariate Gelman-Rubin (GR) diagnostic was $\leq$1·1 for all inferred parameters [10], and the effective sample size (ESS) for the combined

chains was sufficiently large. We retained a sample of 1,000 posterior parameter sets and corresponding epidemic trajectories between 2010 and 2020 for use in the simulation study.

All computation was performed in R version 4·0·2. The differential equation model was implemented using odin version 1·1·7 [11], the MCMC fitting algorithm was adapted from previously developed fitting tools [12], assessment of chain convergence was conducted using coda version 0·19·3 [9].

1.2.6 Results of calibration

Traceplots (Figure S2) show that the posterior parameter space was explored thoroughly and that the chains were consistent with each other. The calibrated model reproduces the data well, with the observations falling within the range of the posterior predictive intervals, except for the low number of diagnoses in 2016 (Figure S3).

The calibrated model successfully captures the temporal trends of increasing tests, increasing diagnoses and the declining proportion of diagnoses that are symptomatic (Figure S3). Between 2010 and 2019 the annual number of gonorrhoea tests in MSM in England increased from 68,600 to 268,000, while the proportion of diagnosed infections that are symptomatic declined from 66·1% to 49·3%, which shows that increased screening (i.e. testing in the absence of symptoms) has contributed to the increase in diagnoses. However, diagnoses increased by more than can be explained by the increase in screening alone. The estimated annual number of symptomatic infections diagnosed increased from 3,300 to 16,700, indicating a simultaneous rise in the underlying incidence of infection, reflecting reported increases in risk behaviour (e.g.[13]). Furthermore, the positivity of those screened has increased from 7·2% to 12·6%, indicating increased prevalence of asymptomatic infection.

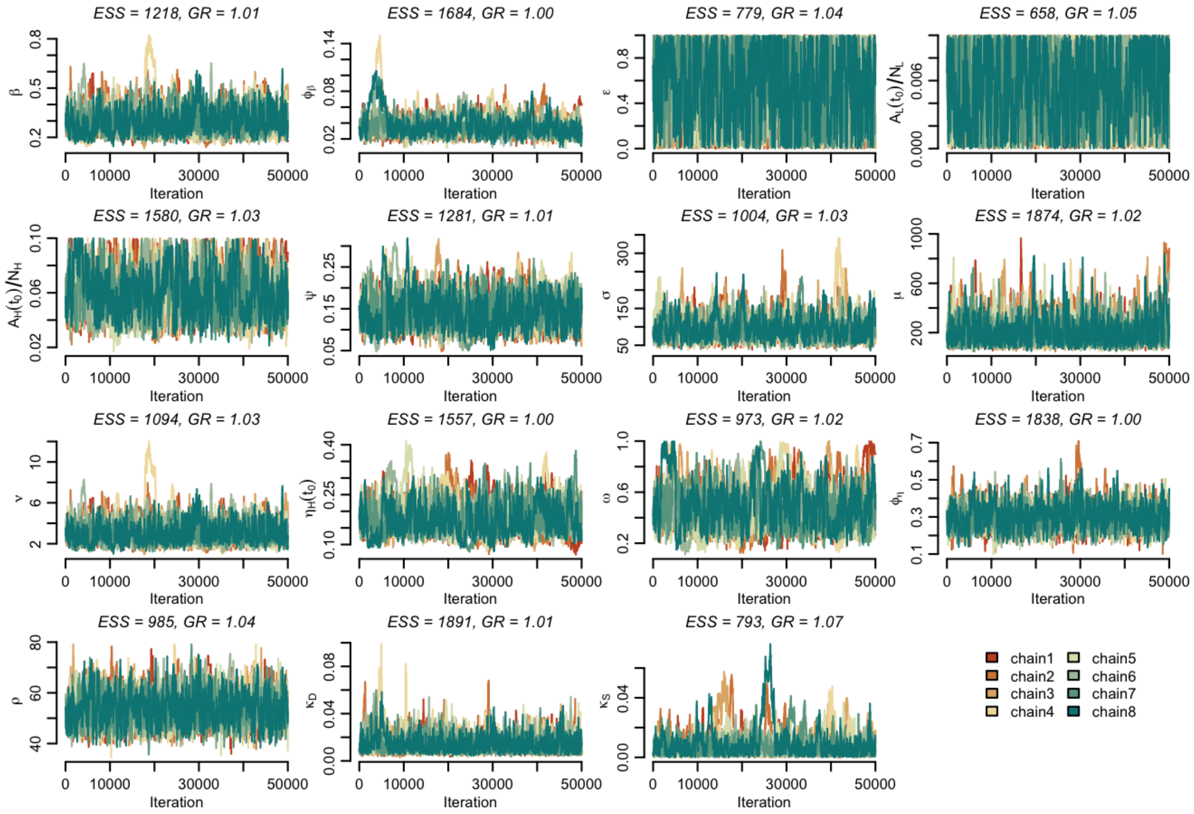

Figure S2: Traceplots of posterior parameter estimates sampled by the MCMC. Coloured lines show each of the eight chains of 50,000 iterations. The effective sample size (ESS) and Gelman-Rubin (GR) diagnostic are displayed above each parameter.

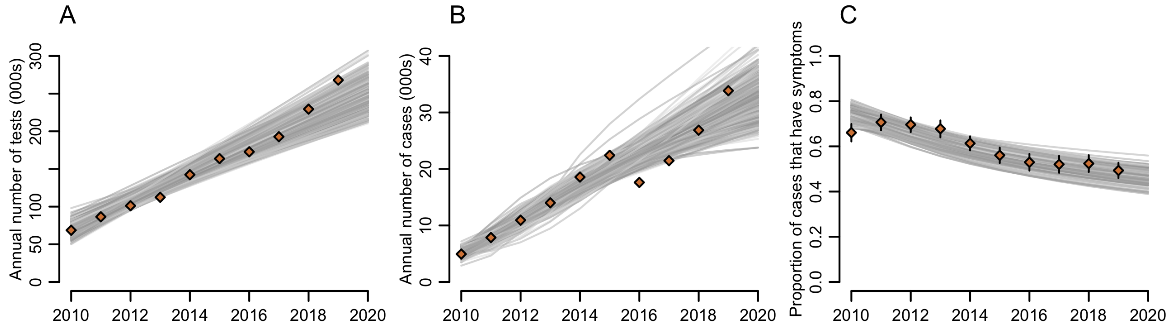

Figure S3: Comparison of simulated epidemic trajectories (based on a sample of 1,000 parameter sets from the joint posterior) and observed data in England MSM. (A) Annual number of gonorrhoea tests (GUMCAD), (B) Annual number of gonorrhoea diagnoses (GUMCAD), (C) Proportion of diagnoses that were symptomatic (GRASP: bars show the 95%CI of a beta distribution based on the GRASP sample size).

Table S2: Fitted parameters: notation, definitions, prior distributions and posterior estimates. Transition rate parameters ($\theta \in \{\sigma, \mu, \nu, \rho\}$) are presented on an annual basis, giving a mean time to transition of $365/\theta$ days.

θ	Definition	Prior distribution	Prior estimate	Posterior estimate
β	Probability of transmission per-partnership	$\mathrm{U}[0, 1]$	0·50 (0·025, 0·975)	0·301 (0·189, 0·504)
ϕ_β	Annual increase in transmission risk behaviour	$\mathrm{U}[0, 1]$	0·50 (0·025, 0·975)	0·036 (0·020, 0·065)
ϵ	Level of assortativity in sexual mixing	$\mathrm{U}[0, 1]$	0·50 (0·025, 0·975)	0·570 (0·040, 0·986)
$\frac{A_L(t_0)}{N_L}$	Initial prevalence of asymptomatic infection in group L	$\mathrm{U}[0, 0{\cdot}01]$	0·005 (0·0003, 0·0098)	0·0057 (0·0005, 0·0099)
$\frac{A_H(t_0)}{N_H}$	Initial prevalence of asymptomatic infection in group H	$\mathrm{U}[0, 0{\cdot}10]$	0·05 (0·0025, 0·0975)	0·0586 (0·0343, 0·0931)
ψ	Probability that incident infection is symptomatic	$\mathrm{U}[0, 1]$	0·50 (0·025, 0·975)	0·150 (0·0737, 0·238)
σ	Rate of leaving incubation period ($I \to S/A$)	$\Gamma(14{\cdot}9, 0{\cdot}0009)$	77·7 (49·5, 139)	99·9 (56·1, 176)
μ	Rate of seeking treatment due to symptoms ($S \to T$)	$\Gamma(7{\cdot}3, 0{\cdot}001)$	135 (73·2, 329)	218 (92·9, 521)
ν	Rate of natural recovery ($A \to U$)	$\Gamma(12{\cdot}3, 0{\cdot}035)$	2·32 (1·43, 4·45)	3·08 (1·60, 6·03)
$\eta_H(t_0)$	Initial rate of asymptomatic screening in group H	$\mathrm{U}[0, 4]$	2 (0·1, 3·9)	0·184 (0·107, 0·300)
ω	Ratio of screening rate in group L vs H	$\mathrm{logN}(-0{\cdot}87, 0{\cdot}39)$	0·48 (0·196, 0·888)	0·475 (0·218, 0·876)
ϕ_η	Annual increase in screening rate	$\mathrm{U}[0, 1]$	0·50 (0·025, 0·975)	0·302 (0·193, 0·440)
ρ	Rate of recovery after treatment ($T \to U$)	$\Gamma(94{\cdot}1, 0{\cdot}0002)$	52·1 (43, 64·5)	54·0 (43·6, 66·4)
κ_D	Shape parameter of GUMCAD data	$\mathrm{U}[0, 1]$	0·50 (0·025, 0·975)	0·0146 (0·0061, 0·0320)
κ_S	Shape parameter of GRASP data	$\mathrm{U}[0, 1]$	0·50 (0·025, 0·975)	0·0081 (0·0007, 0·0312)

1.3 Model of vaccination

We extended the fitted transmission-dynamic model to include vaccination with an imperfect vaccine, allowing for differing levels of vaccine uptake, vaccine efficacy against infection (e) and waning of protection (after mean duration D_V) (Figure S4). We assume partially-efficacious vaccines offer 'leaky', or 'degree-type', protection [14], reducing the probability of infection upon sexual contact with an infectious partner, so it is still possible, but less likely, for vaccine-protected individuals to become infected. We assume that vaccination status does not affect an individual's progression through stages of infection or their likelihood of infecting a partner. The infectious population in group j therefore incorporates individuals in all three vaccination-status strata (X = unvaccinated, V = vaccine-protected, W = waned), so that:

$$C_j(t) = \sum_{i \in \{X,V,W\}} \left(I_j^i(t) + A_j^i(t) + S_j^i(t)\right) \tag{14}$$

After vaccine protection has waned, an individual's susceptibility to infection returns to the same level as before they were vaccinated. Individuals whose vaccine protection has waned can be re-vaccinated, with a single booster dose restoring protection.

The force of infection (before accounting for any vaccine protection) is then defined similarly to Equation 1;

$$\lambda_j(t) = c_j \beta(1 + \phi_\beta(t - t_0)) \left(\epsilon \frac{C_j(t)}{N_j(t)} + (1 - \epsilon) \left(\sum_{i \in \{L,H\}} \pi_i(t) \frac{C_i(t)}{N_i(t)} \right) \right) \tag{15}$$

In our model, vaccination may occur at three points: before entry into the sexually-active population (p^{VbE}), on gonorrhoea diagnosis (p^{VoD}), or when testing negative (p_j^{VoS}) if vaccination is offered to those attending for screening. We consider three potential approaches to targeting vaccination (which could be used singly or in combination):

- Vaccination-before-entry into the population (VbE) – where adolescents are vaccinated in schools before they become sexually active;
- Vaccination-on-diagnosis (VoD): vaccination is offered to MSM diagnosed with gonorrhoea;
- Vaccination-on-attendance (VoA = VoD + VoS): vaccination is offered to all MSM tested for gonorrhoea (regardless of whether they are infected or not);
- Vaccination-according-to-risk (VaR), with VoD offered to patients in the low-activity group and VoA offered to patients in the high-activity group. This is equivalent to VoD offered to all patients and VoS offered to those in the high-activity group.

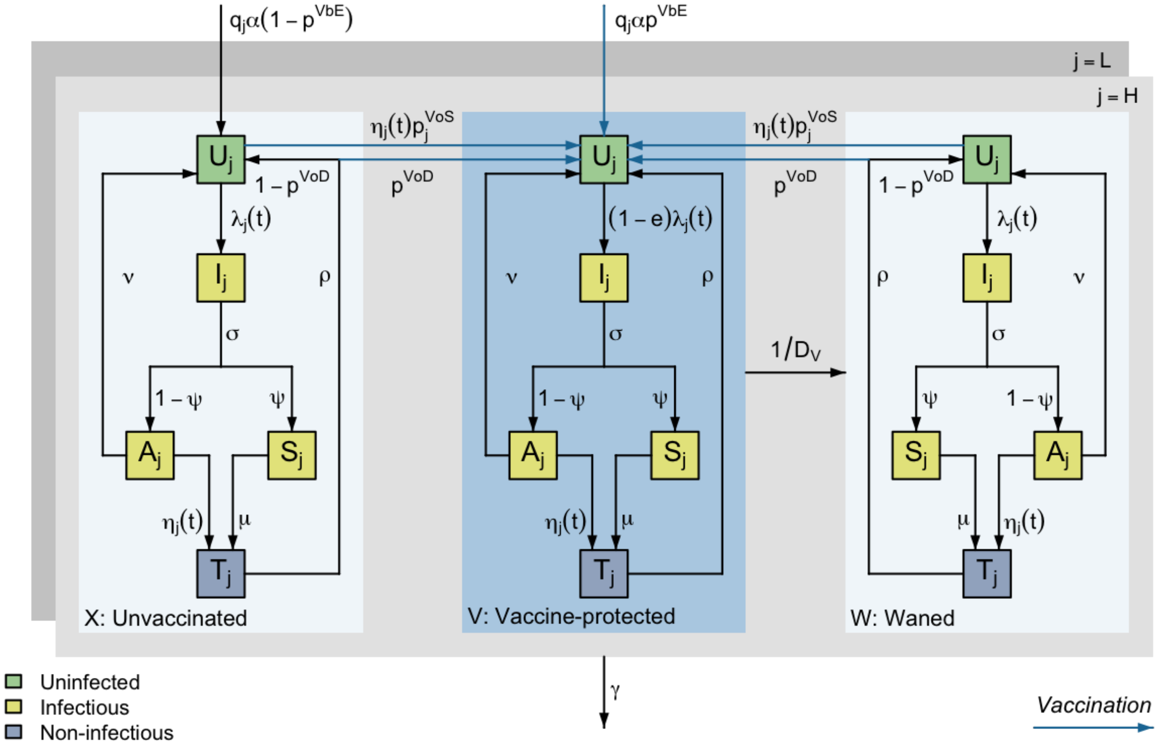

Figure S4: Model-structure diagram showing the epidemiology of gonorrhoea and vaccination. The population is divided into compartments representing different states of infection and sexual activity groups (as described in Figure S1), and is further divided to represent states of vaccine protection (Unvaccinated, Vaccinated, and Waned). Individuals entering the sexually-active population are uninfected (U); a proportion (p^{VbE}) receive adolescent vaccination (under VbE) and enter the Vaccine-protected stratum, whilst the remainder ($1 - p^{\mathrm{VbE}}$) enter the Unvaccinated stratum. When vaccine protection wanes (on average after D_V years) individuals are no longer protected and move from the relevant compartment in the Vaccine-protected stratum to the corresponding compartment in the Waned stratum. Vaccine protection reduces susceptibility to infection by $(1 - e)$, and where $e < 100\%$ vaccinated individuals may still become infected, with the course of infection proceeding as described in Figure S1. Individuals in group j who have not been vaccinated, or whose protection has waned, may be vaccinated upon diagnosis with gonorrhoea (p^{VoD}) or upon attending a sexual health clinic for screening (p_j^{VoS}), depending on the vaccination strategy in operation. For clarity only flows in and out of the upper layer (the high-activity group) are shown.

1.3.1 Compartmental model equations

We describe the model depicted in Figure S4 in differential equations. Each compartment is stratified by sexual activity group $j \in \{L, H\}$ and according to vaccination status: unvaccinated (X), vaccine-protected (V) or waned (W). Full definitions of model parameters are set out in Table S1, Table S2 and Table S3.

$$\frac{dU_j^X(t)}{dt} = q_j\alpha(1-p^{\text{VbE}}) - (\lambda_j(t) + p_j^{\text{VoS}}\eta_j(t) + \gamma)U_j^X(t) + \nu A_j^X(t) + (1-p^{\text{VoD}})\rho T_j^X(t) \tag{16}$$

$$\frac{dI_j^X(t)}{dt} = \lambda_j(t)U_j^X(t) - (\sigma+\gamma)I_j^X(t) \tag{17}$$

$$\frac{dA_j^X(t)}{dt} = (1-\psi)\sigma I_j^X(t) - (\nu + \eta_j(t) + \gamma)A_j^X(t) \tag{18}$$

$$\frac{dS_j^X(t)}{dt} = \psi\sigma I_j^X(t) - (\mu+\gamma)S_j^X(t) \tag{19}$$

$$\frac{dT_j^X(t)}{dt} = \eta_j(t)A_j^X(t) + \mu S_j^X(t) - (\rho+\gamma)T_j^X(t) \tag{20}$$

$$\begin{aligned}\frac{dU_j^V(t)}{dt} &= q_j\alpha p^{\text{VbE}} + p_j^{\text{VoS}}\eta_j(t)U_j^X(t) + p^{\text{VoD}}\rho T_j^X(t) - \Big((1-e)\lambda_j(t) + \frac{1}{D_V} + \gamma\Big)U_j^V(t) \\ &\quad + \nu A_j^V(t) + \rho T_j^V(t) + p_j^{\text{VoS}}\eta_j(t)U_j^W(t) + p^{\text{VoD}}\rho T_j^W(t)\end{aligned} \tag{21}$$

$$\frac{dI_j^V(t)}{dt} = (1-e)\lambda_j(t)U_j^V(t) - \Big(\sigma + \frac{1}{D_V} + \gamma\Big)I_j^V(t) \tag{22}$$

$$\frac{dA_j^V(t)}{dt} = (1-\psi)\sigma I_j^V(t) - \Big(\nu + \eta_j(t) + \frac{1}{D_V} + \gamma\Big)A_j^V(t) \tag{23}$$

$$\frac{dS_j^V(t)}{dt} = \psi\sigma I_j^V(t) - \Big(\mu + \frac{1}{D_V} + \gamma\Big)S_j^V(t) \tag{24}$$

$$\frac{dT_j^V(t)}{dt} = \eta_j(t)A_j^V(t) + \mu S_j^V(t) - \Big(\rho + \frac{1}{D_V} + \gamma\Big)T_j^V(t) \tag{25}$$

$$\frac{dU_j^W(t)}{dt} = \frac{U_j^V(t)}{D_V} - (\lambda_j(t) + p_j^{\text{VoS}}\eta_j(t) + \gamma)U_j^W(t) + \nu A_j^W(t) + (1-p^{\text{VoD}})\rho T_j^W(t) \tag{26}$$

$$\frac{dI_j^W(t)}{dt} = \frac{I_j^V(t)}{D_V} + \lambda_j(t)U_j^W(t) - (\sigma+\gamma)I_j^W(t) \tag{27}$$

$$\frac{dA_j^W(t)}{dt} = \frac{A_j^V(t)}{D_V} + (1-\psi)\sigma I_j^W(t) - (\nu + \eta_j(t) + \gamma)A_j^W(t) \tag{28}$$

$$\frac{dS_j^W(t)}{dt} = \frac{S_j^V(t)}{D_V} + \psi\sigma I_j^W(t) - (\mu+\gamma)S_j^W(t) \tag{29}$$

$$\frac{dT_j^W(t)}{dt} = \frac{T_j^V(t)}{D_V} + \eta_j(t)A_j^W(t) + \mu S_j^W(t) - (\rho+\gamma)T_j^W(t) \tag{30}$$

1.3.2 Simulations

Using 1,000 sets of parameters sampled from the joint posterior of the transmission model (described in Equations 4-8), we performed deterministic forward simulations of gonorrhoea transmission in MSM using the vaccination model (described in Equations 16-30). As the future trajectory of the epidemic is uncertain we considered two alternative behavioural baseline scenarios: a lower-bound (assuming that the inferred trends in the time-varying parameters stabilise) and an upper-bound (assuming that trends continue until the end of the period we model).

We compared the number of gonorrhoea cases averted, number of vaccine doses administered, and health-economic value of vaccination for:

- two behavioural baseline scenarios; and
- four vaccine-targeting strategies (VbE, VoD, VaR, VoA) against a background of no vaccination, and three vaccine-targeting strategies in sexual health clinics (VoD, VaR, VoA) against a background of adolescent vaccination in schools (VbE); and
- two time-horizons (10, 20 years); and
- three levels of vaccine uptake in sexual health clinics (low / central / high); for
- vaccines of varying efficacy (1%–100%) and duration of protection (1–20 years).

Proceeding similarly to the derivation of Equations 10-12, for each vaccine stratum $i \in \{X, V, W\}$ and calendar year t, the total number of diagnosed cases, $Y_D^i(t)$; the number of symptomatic and asymptomatic diagnoses, $Y_S^i(t), Y_A^i(t)$; and the number of uninfected patients screened for gonorrhoea, $Y_U^i(t)$, are given by:

$$Y_D^i(t) = \sum_{j \in \{L,H\}} \int_t^{t+1} \rho T_j^i(\tau) d\tau \tag{31}$$

$$Y_S^i(t) = \sum_{j \in \{L,H\}} \int_t^{t+1} \mu S_j^i(\tau) d\tau \tag{32}$$

$$Y_A^i(t) = \sum_{j \in \{L,H\}} \int_t^{t+1} \eta_j(\tau) A_j^i(\tau) d\tau \tag{33}$$

$$Y_U^i(t) = \sum_{j \in \{L,H\}} \int_t^{t+1} \eta_j(\tau) U_j^i(\tau) d\tau \tag{34}$$

1.3.3 Vaccine uptake and calculation of total doses

We assume that primary vaccination requires two doses, and that revaccination after protection has waned requires a single booster dose. The average number of doses per person protected by vaccination in sexual health clinics is therefore,

$$\begin{cases} \frac{r_1(1-r_2)+2r_1r_2}{r_1r_2} = \frac{1+r_2}{r_2} & \text{for primary vaccination} \\ 1 & \text{for revaccination} \end{cases} \tag{35}$$

Where r_1 is the proportion receiving the first dose and r_2 is proportion who receive the second dose, given they have accepted the first. We assume that uptake in sexual health clinics is the same as for HPV vaccination of MSM in sexual health clinics, i.e. 33·0%(95%CI:32·7%–33·3%), with 42·8% (32,562/76,033) of those offered vaccination receiving a first dose and 77·1% (11,267/14,612) of those receiving a second [15]. (We used data on observed uptake rather than reported vaccine acceptability in surveys because 58.3%(95%CI:53.8%–62.7%) of MSM attending sexual health services said they would "definitely" have the HPV vaccine if offered and a further 25.1%(21.3%–29.2%) would "probably" have it [16], which is much greater than the uptake which has occurred now the vaccine is available.) We reflected the uncertainty in the parameter estimates by assigning Beta distributions to r_1 and r_2:

$$r_1 \sim \text{Beta}(32562, 43471) \tag{36}$$

$$r_2 \sim \text{Beta}(11267, 3345) \tag{37}$$

Vaccination in sexual health clinics is offered to unvaccinated MSM and those whose protection has waned, i.e. those in strata $i \in \{X, W\}$. For VbE we assume that all who receive the first dose also receive the second. We denote the total number of vaccine doses administered in each stratum in year t as $V_i(t)$, where:

$$V^X(t) = 2\alpha p^{\text{VbE}} + \frac{1 + r_2}{r_2}\left(p_j^{\text{VoS}} Y_U^X(t) + p^{\text{VoD}} Y_D^X(t)\right) \tag{38}$$

$$V^W(t) = p_j^{\text{VoS}} Y_U^W(t) + p^{\text{VoD}} Y_D^W(t) \tag{39}$$

Therefore, the total number of doses administered in year t across the whole population is:

$$V(t) = V^X(t) + V^W(t) \tag{40}$$

We generated 1,000 samples from the distributions of r_1 and r_2, to calculate an overall central uptake scenario of $u = r_1 r_2 = 33{\cdot}0\%$(95%CI:32·7%–33·3%). To assess the sensitivity of our results to this assumption, we considered two alternative scenarios, "low" and "high", halving and doubling the observed uptake, to 16·5%(16·3%–16·7%) and 66·0%(65·4%–66·6%), respectively, by changing r_1.

Table S3: Vaccination parameters used in scenario analysis.

	Parameter	Values	Source
p^{VbE}	Uptake of adolescent vaccination	86·7%*	[17]
p^{VoD}	Uptake of vaccination on diagnosis	$u, 0{\cdot}5u, 2u$*	[15]
p_j^{VoS}	Uptake of vaccination on screening by uninfected individuals in group j	$u, 0{\cdot}5u, 2u$*	[15]
e	Vaccine efficacy against infection	1–100%	-
D_V	Duration of protection	1–20 years	-

* *The parameter value is 0 in scenarios where the mode of vaccination does not apply*

1.3.4 Comparison baselines

We assessed the impact and health-economic value of vaccination in sexual health clinics using the two behavioural baseline scenarios, either with or without an adolescent vaccination programme in schools.

For each of the scenarios set out in Table S3, we calculate the number of diagnosed cases in year t across all vaccination-status strata $i \in \{X, V, W\}, Y_D(t) = \sum_i Y_D^i(t)$, and compare this to the baseline $(\hat{Y}_D(t))$ to give the total cases averted over M years:

$$\sum_{t=0}^{M-1} \hat{Y}_D(t_0 + t) - Y_D(t_0 + t) \tag{41}$$

Similarly, using the definition set out in Equation 40, the number of vaccine doses administered over M years, relative to the baseline $(\hat{V}(t))$ is:

$$\sum_{t=0}^{M-1} V(t_0 + t) - \hat{V}(t_0 + t) \tag{42}$$

1.3.5 Health-economic analysis

Unit costs for testing of patients with and without symptoms, management of infection, and test-of-cure (ToC) were based on literature. All prices were adjusted to 2018-19 GB £ values using the Hospital and Community Health Services (HCHS) index [18] and NHS Cost Inflation Index (NHSCII) [19]. Uncertainty in unit costs was represented using a Gamma distribution with relative standard deviation of 20%.

Table S4: Health economic parameters.

	Description	Mean (95%CI)	Distribution	Source
w^U	Cost of initial test (£)	88·35 (57·57, 123·16)	Gamma(88·35, 0·2)*	[20]
w^S	Cost of investigating symptoms (£)	21·72 (14·59, 31·90)	Gamma(21·72, 0·2)*	[20]
w^T	Cost of treatment (£)	79·82 (52·55, 117·50)	Gamma(79·82, 0·2)*	[20]
w^Q	Value of a QALY (£)	20,000, 30,000	-	
w^{ToC}	Cost of ToC (£)	44·28 (28·83, 63·99)	Gamma(44·28, 0·2)*	[20]
p^{ToC}	Proportion returning for ToC	0·57 (0·54, 0·60)	Beta(552, 419)	[4]
d^S	QoL disutility of symptoms	0·160 (0·136, 0·182)	Pert(0·128, 0·16, 0·192)	[21, 22]
z^T	Reduction in cost of treatment at initial visit	14%		[20]

**Gamma distributions parameterised in terms of mean and standard deviation*

The proportion of treated patients returning for test-of-cure (p^{ToC}) was sampled from a Beta distribution based on GRASP data [4]: $p^{\text{ToC}} \sim \text{Beta}(552, 419)$, giving $p^{\text{ToC}} = 56{\cdot}8\%$(95%CI:53·7%–60·0%).

For each year, t, we sum across all vaccination-status strata, $i \in \{X, V, W\}$, to calculate the total number of symptomatic patients diagnoses, $Y_S(t) = \sum_i Y_S^i(t)$; the total number of asymptomatic diagnoses, $Y_A(t) = \sum_i Y_A^i(t)$; and the total number of uninfected patients screened for gonorrhoea $Y_U(t) = \sum_i Y_U^i(t)$.

The severity of symptoms is measured by the quality-of-life (QoL) disutility, d^S=0·16, which was obtained from literature [21, 22]. We represented uncertainty using a Pert distribution with range ±20% such that: $d^S \sim \text{Pert}\big(0{\cdot}128, 0{\cdot}16, 0{\cdot}192\big)$, giving a central estimate of $d^S = 0{\cdot}160$(95%CI:0·136–0·182).

QALY loss is the product of the QoL disutility of symptoms and the average duration of symptoms. This average duration was assumed to be the time until obtaining care $(1/\mu)$ plus half the duration of treatment $(1/2\rho)$, with uncertainty in those parameters being represented by the posterior distribution. We assessed health-economic value with a QALY valued at £20,000 or £30,000, as is standard UK practice [23].

The total value of healthcare costs and QALY losses for uninfected $(W_U(t))$, asymptomatic $(W_A(t))$, and

symptomatic individuals ($W_S(t)$) in year t are:

$$W_U(t) = w^U Y_U(t) \tag{43}$$

$$W_A(t) = \left(w^U + w^T + p^{\mathrm{ToC}} w^{\mathrm{ToC}}\right) Y_A(t) \tag{44}$$

$$W_S(t) = \left(w^U + w^S + d^S \left(\frac{1}{\mu} + \frac{1}{2\rho}\right) w^Q + (1 - z^T) w^T + p^{\mathrm{ToC}} w^{\mathrm{ToC}}\right) Y_S(t) \tag{45}$$

The total value for all categories of individual combined in year t is:

$$W(t) = W_U(t) + W_A(t) + W_S(t) \tag{46}$$

We derive the monetary benefit of vaccination in year t by comparing $W(t)$ for a given vaccination scenario (defined by vaccine targeting strategy, uptake, vaccine efficacy and duration of protection), to the appropriate baseline $\hat{W}(t)$. We discount monetary benefit accruing in future years to its present value at rate $d = 3{\cdot}5\%$ [23].

To derive the value per dose (i.e. the maximum cost at which vaccination would be cost-effective) for a given vaccination scenario assessed over M years, we divide the present value of the monetary benefit of vaccination by the present value of the additional number of vaccine doses given relative to the baseline ($\hat{V}(t)$, which may include adolescent vaccination against MenB). The value per dose is therefore derived as follows:

$$\mathrm{VPD} = \frac{\sum_{t=0}^{M-1} \left(\hat{W}(t_0 + t) - W(t_0 + t)\right)\left(1 + d^{-(t+0{\cdot}5)}\right)}{\sum_{t=0}^{M-1} \left(V(t_0 + t) - \hat{V}(t_0 + t)\right)\left(1 + d^{-(t+0{\cdot}5)}\right)} \tag{47}$$

The health-economic analysis is summarised in the box below.

Target population, setting and location: men-who-have-sex-with-men (MSM) in England
Perspective: sexual health clinics in the National Health Service (NHS)
Comparisons: vaccination in sexual health clinics vs (i) no vaccination, using a lower-bound baseline which assumes that behavioural trends stabilise; (ii) no vaccination, using an upper-bound baseline which assumes that behavioural trends continue; (iii) vaccination of adolescents in schools, using the lower-bound epidemic baseline; and (iv) vaccination of adolescents in schools, using the upper-bound epidemic baseline
Time horizons: 10 & 20 years
Discount rate: 3·5%p.a.
Health outcomes: QALY losses due to symptoms (vaccination gains QALYs by averting losses)
Measurement of effectiveness: vaccine efficacy varied in the range 1–100%, and duration of protection varied in the range 1–20 years
Currency: 2018-19 GB £
Willingness-to-pay: £20,000/QALY, with £30,000/QALY used in sensitivity analysis

1.4 Model of differential waning of protection after primary vaccination vs repeat vaccination

The Joint Committee on Vaccination and Immunisation (JCVI) has suggested that 4CMenB provides a longer duration of protection after a booster dose than after primary vaccination [24]. We therefore adapted the model described in Section 1.3, to allow the duration of protection to differ for primary and re-vaccination (Figure S5).

The infectious population in group j now incorporates individuals in four vaccination-status strata (X = unvaccinated, V = vaccine-protected, W = waned, R = re-vaccinated), so that:

$$C_j(t) = \sum_{i \in \{X,V,W,R\}} \left(I_j^i(t) + A_j^i(t) + S_j^i(t) \right) \tag{48}$$

The rate of transmission (before accounting for any vaccine protection) is then defined similarly to Equation 1;

$$\lambda_j(t) = c_j \beta (1 + \phi_\beta (t - t_0)) \left(\epsilon \frac{C_j(t)}{N_j(t)} + (1 - \epsilon) \left(\sum_{i \in \{L,H\}} \pi_i(t) \frac{C_i(t)}{N_i(t)} \right) \right) \tag{49}$$

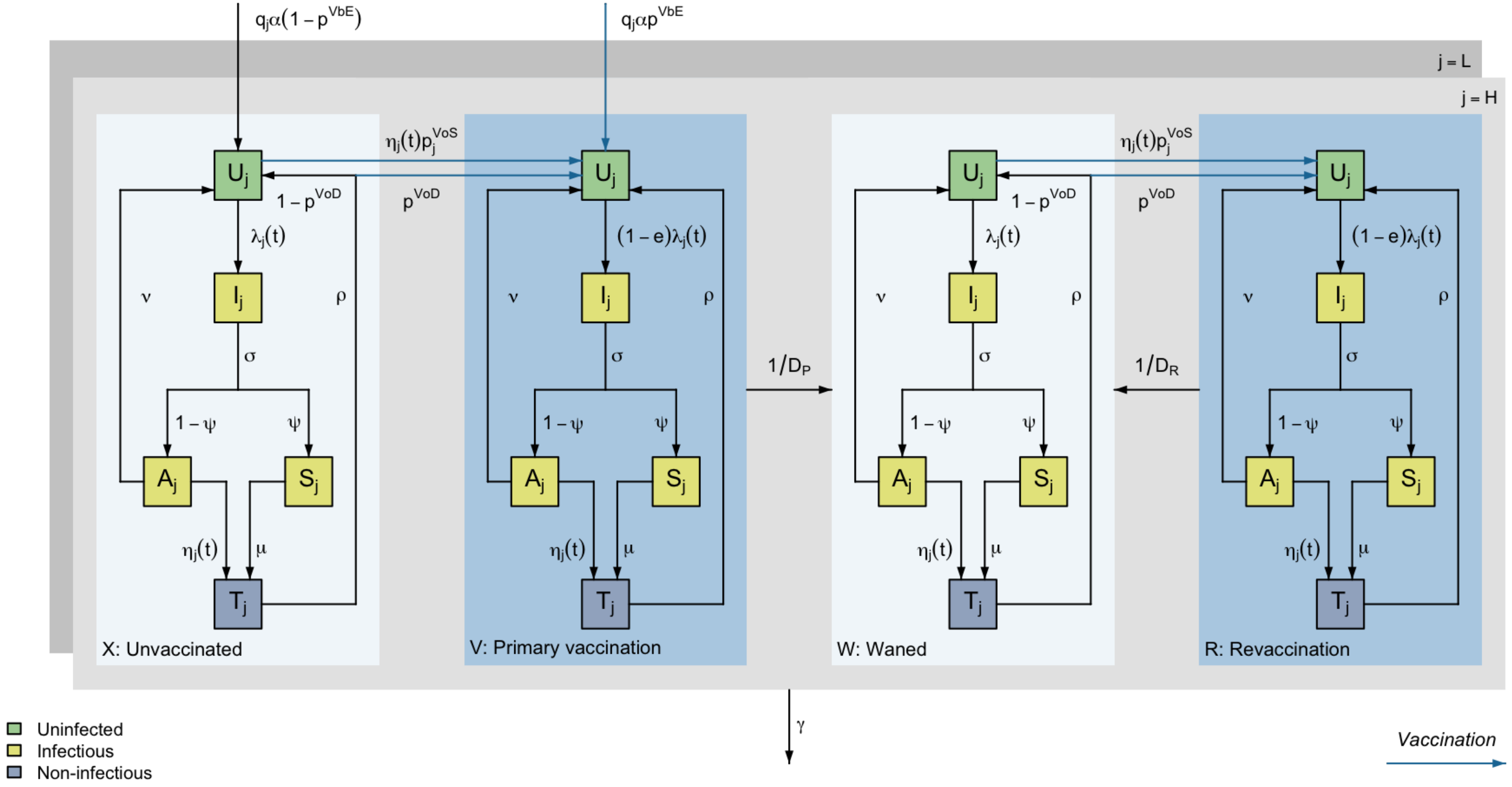

Figure S5: Model-structure diagram showing the epidemiology of gonorrhoea and vaccination, allowing the average duration of protection to differ following primary and re-vaccination (respectively, D_P and D_R years). The population is divided into compartments representing different states of infection, sexual activity groups (for clarity only flows in and out of the upper layer are shown) and states of vaccine protection (as described in Figure S4), with an additional vaccine-protected stratum for revaccinated individuals.

1.4.1 Compartmental model equations

We describe the model depicted in Figure S5 in differential equations. Each compartment is stratified by sexual activity group $j \in \{L, H\}$ and according to vaccination status: unvaccinated (X), protected after primary vaccination (V), waned (W), and protected after revaccination (R). Full definitions of model parameters are set out in Tables S1, S2 and S5.

$$\frac{dU_j^X(t)}{dt} = q_j\alpha(1-p^{\text{VbE}}) - (\lambda_j(t) + p_j^{\text{VoS}}\eta_j(t) + \gamma)U_j^X(t) + \nu A_j^X(t) + (1-p^{\text{VoD}})\rho T_j^X(t) \tag{50}$$

$$\frac{dI_j^X(t)}{dt} = \lambda_j(t)U_j^X(t) - (\sigma+\gamma)I_j^X(t) \tag{51}$$

$$\frac{dA_j^X(t)}{dt} = (1-\psi)\sigma I_j^X(t) - (\nu + \eta_j(t) + \gamma)A_j^X(t) \tag{52}$$

$$\frac{dS_j^X(t)}{dt} = \psi\sigma I_j^X(t) - (\mu+\gamma)S_j^X(t) \tag{53}$$

$$\frac{dT_j^X(t)}{dt} = \eta_j(t)A_j^X(t) + \mu S_j^X(t) - (\rho+\gamma)T_j^X(t) \tag{54}$$

$$\frac{dU_j^V(t)}{dt} = q_j\alpha p^{\text{VbE}} + p_j^{\text{VoS}}\eta_j(t)U_j^X(t) + p^{\text{VoD}}\rho T_j^X(t) - \Big((1-e)\lambda_j(t) + \frac{1}{D_P} + \gamma\Big)U_j^V(t) + \nu A_j^V(t) + \rho T_j^V(t) \tag{55}$$

$$\frac{dI_j^V(t)}{dt} = (1-e)\lambda_j(t)U_j^V(t) - \Big(\sigma + \frac{1}{D_P} + \gamma\Big)I_j^V(t) \tag{56}$$

$$\frac{dA_j^V(t)}{dt} = (1-\psi)\sigma I_j^V(t) - \Big(\nu + \eta_j(t) + \frac{1}{D_P} + \gamma\Big)A_j^V(t) \tag{57}$$

$$\frac{dS_j^V(t)}{dt} = \psi\sigma I_j^V(t) - \Big(\mu + \frac{1}{D_P} + \gamma\Big)S_j^V(t) \tag{58}$$

$$\frac{dT_j^V(t)}{dt} = \eta_j(t)A_j^V(t) + \mu S_j^V(t) - \Big(\rho + \frac{1}{D_P} + \gamma\Big)T_j^V(t) \tag{59}$$

$$\frac{dU_j^W(t)}{dt} = \frac{U_j^V(t)}{D_P} + \frac{U_j^R(t)}{D_R} - (\lambda_j(t) + p_j^{\text{VoS}}\eta_j(t) + \gamma)U_j^W(t) + \nu A_j^W(t) + (1-p^{\text{VoD}})\rho T_j^W(t) \tag{60}$$

$$\frac{dI_j^W(t)}{dt} = \frac{I_j^V(t)}{D_P} + \frac{I_j^R(t)}{D_R} + \lambda_j(t)U_j^W(t) - (\sigma+\gamma)I_j^W(t) \tag{61}$$

$$\frac{dA_j^W(t)}{dt} = \frac{A_j^V(t)}{D_P} + \frac{A_j^R(t)}{D_R} + (1-\psi)\sigma I_j^W(t) - (\nu + \eta_j(t) + \gamma)A_j^W(t) \tag{62}$$

$$\frac{dS_j^W(t)}{dt} = \frac{S_j^V(t)}{D_P} + \frac{S_j^R(t)}{D_R} + \psi\sigma I_j^W(t) - (\mu+\gamma)S_j^W(t) \tag{63}$$

$$\frac{dT_j^W(t)}{dt} = \frac{T_j^V(t)}{D_P} + \frac{T_j^R(t)}{D_R} + \eta_j(t)A_j^W(t) + \mu S_j^W(t) - (\rho+\gamma)T_j^W(t) \tag{64}$$

$$\frac{dU_j^R(t)}{dt} = p_j^{\text{VoS}}\eta_j(t)U_j^W(t) + p^{\text{VoD}}\rho T_j^W(t) - \Big((1-e)\lambda_j(t) + \frac{1}{D_R} + \gamma\Big)U_j^R(t) + \nu A_j^R(t) + \rho T_j^R(t) \tag{65}$$

$$\frac{dI_j^R(t)}{dt} = (1-e)\lambda_j(t)U_j^R(t) - \Big(\sigma + \frac{1}{D_R} + \gamma\Big)I_j^R(t) \tag{66}$$

$$\frac{dA_j^R(t)}{dt} = (1-\psi)\sigma I_j^R(t) - \Big(\nu + \eta_j(t) + \frac{1}{D_R} + \gamma\Big)A_j^R(t) \tag{67}$$

$$\frac{dS_j^R(t)}{dt} = \psi\sigma I_j^R(t) - \Big(\mu + \frac{1}{D_R} + \gamma\Big)S_j^R(t) \tag{68}$$

$$\frac{dT_j^R(t)}{dt} = \eta_j(t)A_j^R(t) + \mu S_j^R(t) - \Big(\rho + \frac{1}{D_R} + \gamma\Big)T_j^R(t) \tag{69}$$

1.4.2 Simulations

Using 1,000 parameter sets sampled from the joint posterior of the transmission model (described in Equations 4-8), we performed forward simulations of gonorrhoea transmission in MSM using the vaccination model (described in Equations 50-69).

We compared the number of gonorrhoea cases averted, number of vaccine doses administered, and health-economic value of vaccination for:

- two behavioural baseline scenarios; and
- three vaccine-targeting strategies (VoD, VaR, VoA); and
- two time-horizons (10, 20 years); and
- vaccines of varying efficacy (1–2·5× the protection estimated for MeNZB: $e_{\text{MeNZB}} = 31\%(95\%\text{CI}{:}21\%\text{–}39\%)$ [25]); and
- vaccines with varying duration of protection after primary and re-vaccination (1–2× the duration estimated by JCVI, which is $D_P = 18, D_R = 36$ months [24]); and
- vaccines with the same duration of protection after primary and re-vaccination (4, 7·5 years [26]).

Table S5: Vaccination parameters used in 4CMenB analysis.

	Parameter	Values	Source
p^{VoD}	Uptake of vaccination on diagnosis	u*	[15]
p_j^{VoS}	Uptake of vaccination on screening by uninfected individuals in group j	u*	[15]
e	Vaccine efficacy against infection	$\{1, 1{\cdot}5, 2, 2{\cdot}5\}\times e_{\text{MeNZB}}$	[25]
D_P	Duration of protection after primary vaccination	18, 27, 36, 48, 90 months	[24, 26]
D_R	Duration of protection after revaccination	36, 54, 72, 48, 90 months	[24, 26]

* *The parameter value is 0 in scenarios where the particular mode of vaccine targeting does not apply*

1.4.3 Health-economic analysis

Proceeding as before, we assessed the impact and health-economic value of vaccination for the whole population by summing across all vaccination-status strata $i \in \{X, V, W, R\}$. For each year t, we calculated the total number of gonorrhoea diagnoses, $Y_D(t) = \sum_i Y_D^i(t)$; the total number symptomatic diagnoses, $Y_S(t) = \sum_i Y_S^i(t)$; the total number of asymptomatic diagnoses, $Y_A(t) = \sum_i Y_A^i(t)$; and the total number of uninfected patients screened for gonorrhoea $Y_U(t) = \sum_i Y_U^i(t)$.

We then calculated the total number of cases averted (Equation 41), the total number of vaccine doses administered (Equation 42), and the value per dose of vaccination (Equation 47) for each of the scenarios set out in Table S5.

Comparison of targeting strategies using a cost-effectiveness efficiency frontier requires specifying a cost for vaccination in order to calculate incremental costs. 4CMenB is currently used by the NHS to protect infants against MenB but the price paid is confidential. Therefore, we compared calculations using two assumed costs. The higher assumed cost is £85 per dose administered, corresponding to the UK list price of £75 per dose [27, 28] plus the £10 administration cost [29]. The lower assumed cost is £18 per dose administered, which is based on the observation that 4CMenB was estimated to be cost-effective for use in infants at £8 per dose (inflation-adjusted) [27], excluding administration cost. Incremental comparisons were visualised using the R package BCEA version 2.3.1.1 [30].

We performed univariate sensitivity analysis for the health economic parameters and the efficacy and duration of protection of 4CMenB against gonorrhoea, whilst accounting for probabilistic uncertainty in the other parameters. Results are presented in tornado plots.

2 Supplementary results

2.1 Effect of vaccine targeting strategy

Figure 2 in the main paper considers vaccination against the lower-bound baseline which assumes that behavioural trends stabilise. This is reproduced here as Figure S6 for comparison with other figures, which present the results of combining vaccination in sexual health clinics with adolescent vaccination (Figures S7, S9), and the results when using the upper-bound baseline which assumes that behavioural trends continue (Figures S8, S9). We find that adolescent vaccination (VbE) has only a marginal effect (Figures S7, S9) because the age-restricted eligibility means that the coverage it achieves is low. The alternative upper-bound baseline results in a greater number of gonorrhoea cases over time and a greater number of vaccine doses administered (except via VbE) but also a greater number of cases averted by vaccination and greater cost-effectiveness of vaccination.

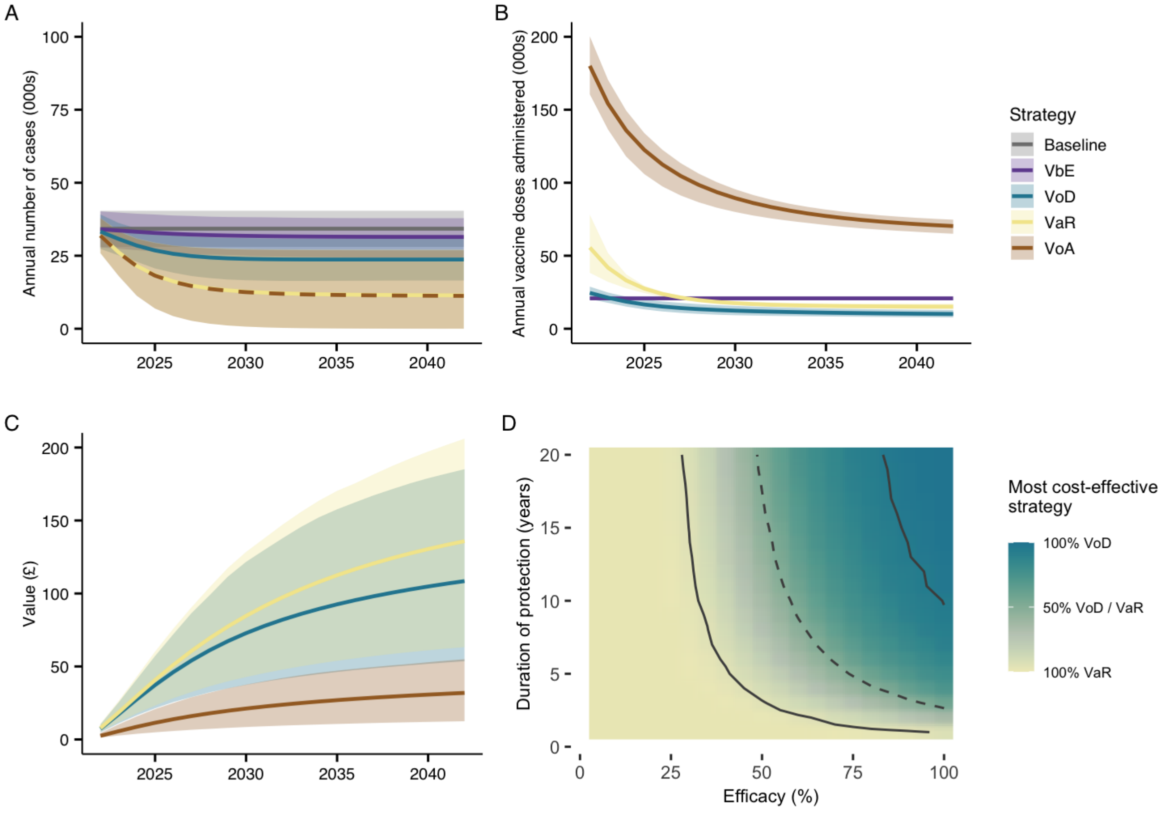

Figure S6: Simulations of gonorrhoea transmission in MSM in England over time under different vaccination strategies. Using a vaccine providing 40% protection for 4 years, panels show (A) Annual gonorrhoea diagnoses (note that the lines for VaR and VoA overlap so VoA is dashed to enable both to be seen), (B) Annual vaccine doses administered, (C) Cumulative value of vaccination per dose administered in sexual health clinics; lines show medians and shading shows 95%CrI. Note that (A) and (B) show undiscounted numbers whilst (C) shows discounted £ values. Panel (D) shows the probability that each strategy is the most cost-effective over 20 years for vaccines ranging in efficacy (1–100%) and duration of protection (1–20 years): in all cases it is either VoD or VaR, and the dashed contour line shows where the two strategies have equal probability of being the most cost-effective whilst the solid contour lines show where VoD (upper right) or VaR (lower left) has 95% probability of being the most cost-effective. Simulations compare each vaccination strategy against no vaccination, using the lower-bound baseline which assumes that behavioural trends stabilise; using 1,000 sets of sampled epidemiological and health-economic parameters; vaccine uptake of 33·0%(95%CI:32·7%–33·3%) for all strategies except VbE, which has 86·7% uptake; and a QALY valued at £20,000.

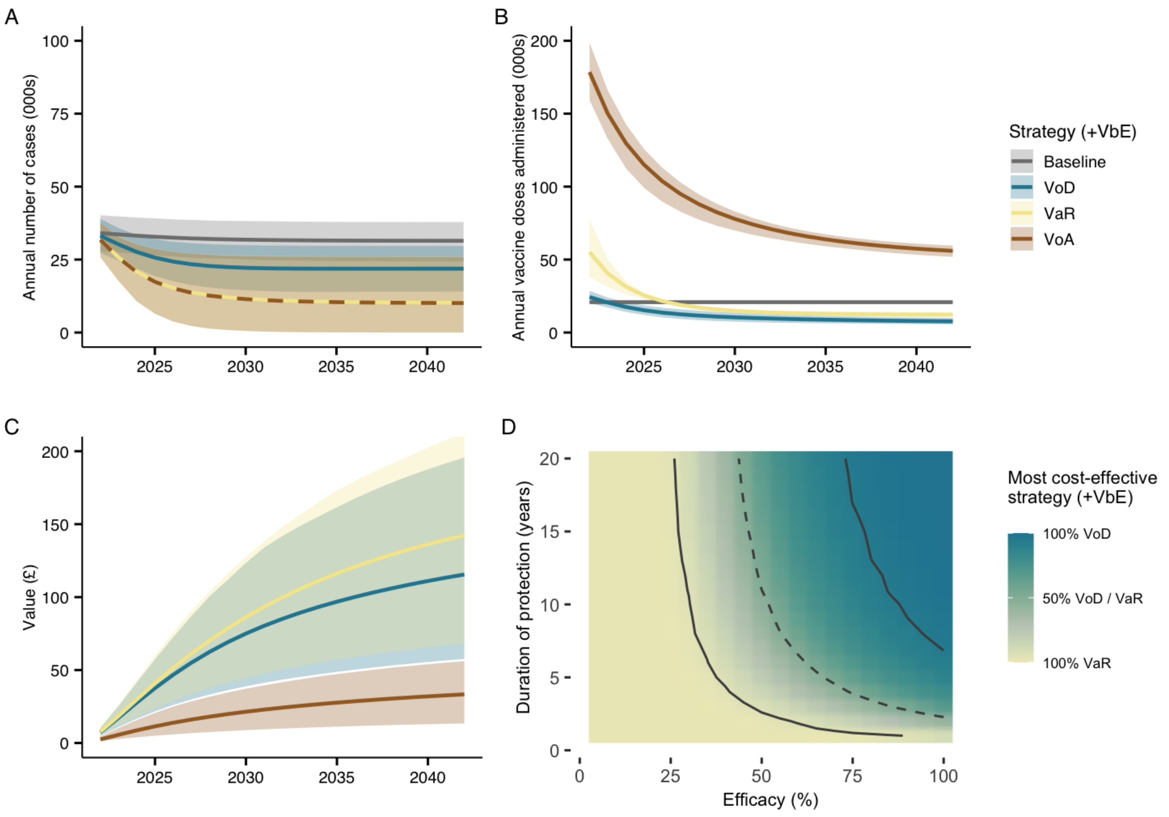

Figure S7: As Figure S6, but comparing vaccination in sexual health clinics (VoD, VaR, or VoA; with 33·0%(95%CI:32·7%–33·3%) uptake) against a background of adolescent vaccination in schools (VbE, with 86·7% uptake).

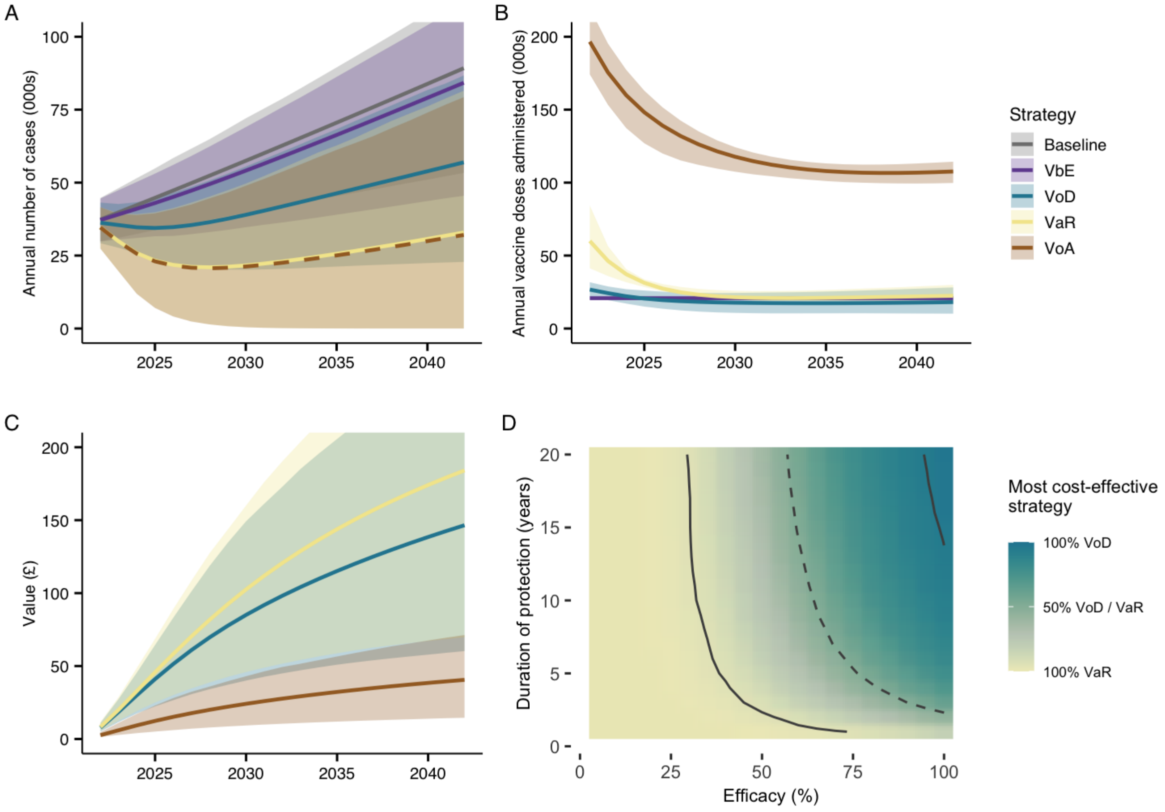

Figure S8: As Figure S6 but using the upper-bound baseline which assumes that behavioural trends continue.

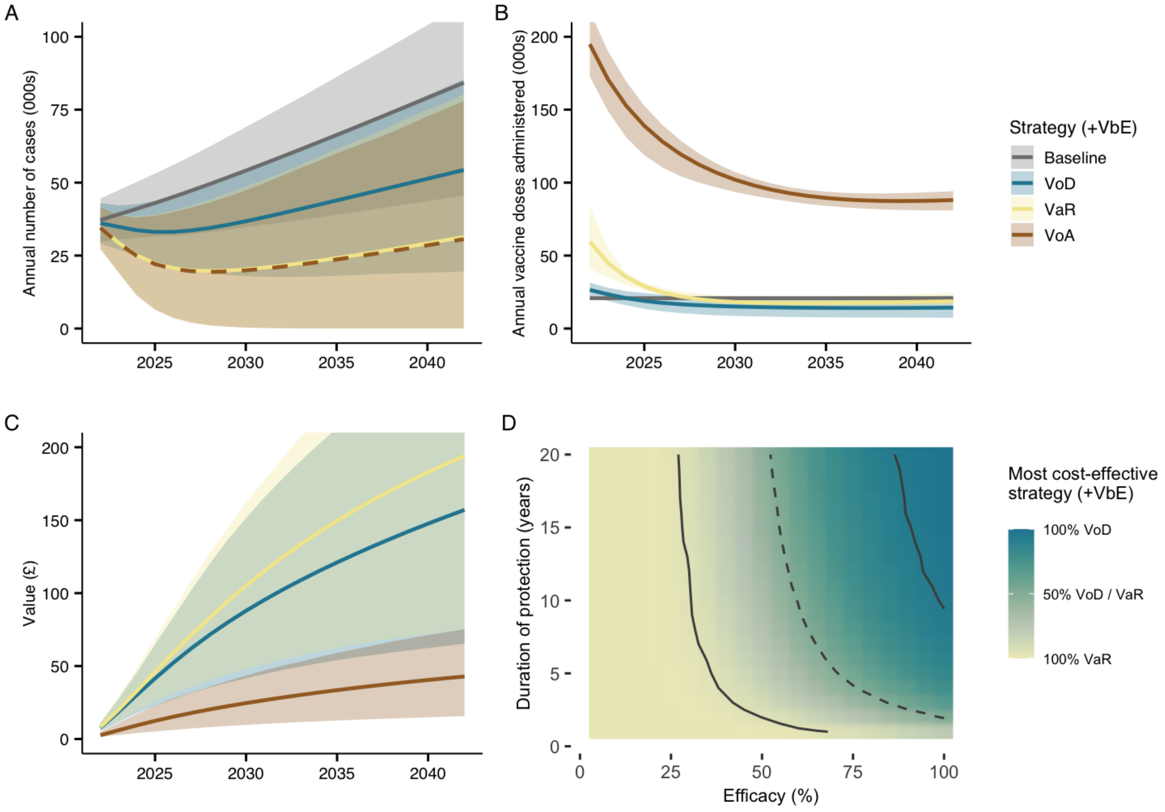

Figure S9: As Figure S7, but using the upper-bound baseline which assumes that behavioural trends continue.

2.2 Effects of vaccine efficacy, duration of protection, targeting strategy, and uptake

Figure 3 in the main paper considers vaccination over a 10-year time-horizon against the lower-bound baseline which assumes that behavioural trends stabilise. This is reproduced here as Figure S10 for comparison with other figures, which present the results of considering a 20-year time-horizon (Figures S11, S13), and the results when using the upper-bound baseline which assumes that behavioural trends continue, considering 10- and 20-year time-horizons (Figures S12, S13). Over a longer time-horizon greater numbers of cases are averted and greater numbers of vaccine doses are administered (Figure S11 vs S10, and S13 vs S12), and as the increase in the former is greater than the increase in the latter the value of vaccination is greater over the longer time-horizon, despite the effects of discounting.

The VoA strategy has the greatest impact, greatest cost, and lowest cost-effectiveness. VoD has the least impact and lowest cost. VaR has impact almost as great as VoA, at a cost intermediate between VoA and VoD. VaR and VoD have similar cost-effectiveness; the former is more cost-effective for vaccines with lower efficacy and duration of protection, and the latter more cost-effective for superior vaccines (Figures S6D, S7D, S8D, S9D & S10C, S11C, S12C, S13C) – although the difference in cost-effectiveness is generally small, and VaR has much greater impact.

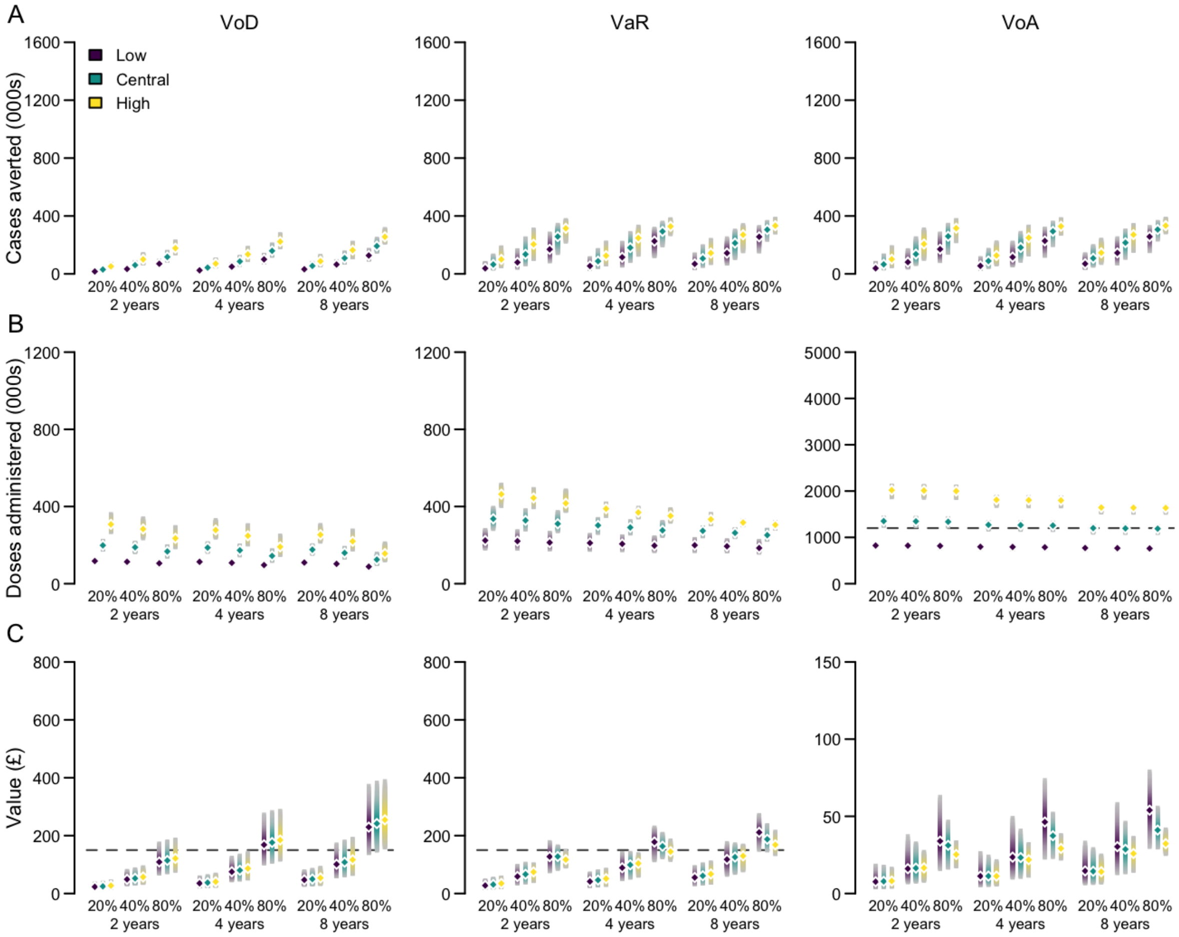

Figure S10: Effects of vaccine uptake, efficacy and duration of protection, on the impact and cost-effectiveness of three vaccination strategies against gonorrhoea in MSM in England over 10 years. Sections show (A) Total cases averted by vaccination, (B) Total number of vaccine doses administered, (C) Value of vaccination per dose administered. Columns of panels compare three targeting strategies: VoD, VaR, VoA. On each plot horizontal axis labels show efficacy (20%, 40%, 80%) and duration of protection (2, 4, 8 years). For each combination of efficacy and duration of protection results are plotted for three levels of uptake: the central uptake scenario (green) assuming uptake is the same as for HPV vaccination of MSM in sexual health clinics (i.e. 33·0%(95%CI:32·7%–33·3%)), which is halved (16·5%, 95%CI:16·3%–16·7%) and doubled (66·0%, 95%CI:65·4%–66·6%) in the low (purple) and high (yellow) uptake scenarios, respectively. Points show medians and shaded bars 95%CrI. Simulations compare each vaccination strategy against no vaccination, using the lower-bound baseline which assumes that behavioural trends stabilise, using 1,000 sets of sampled epidemiological and health-economic parameters, and a QALY valued at £20,000. Note that (A) and (B) show undiscounted numbers whilst (C) shows discounted £ values. Also note that in (B) the dashed line in the VoA panel shows the limit of the scales of the other two panels, and in (C) the dashed lines in the VoD and VaR panels show the limit of the scale of the VoA panel.

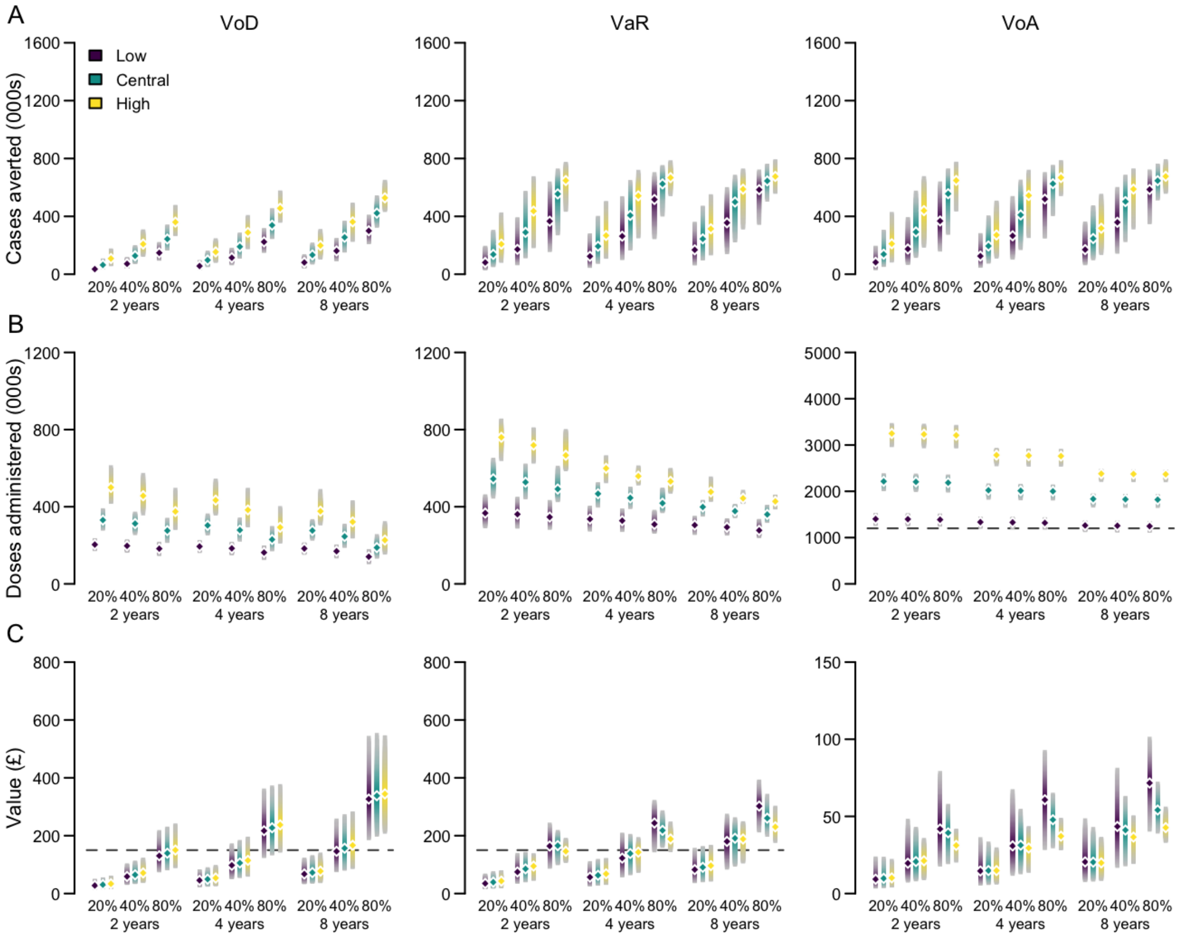

Figure S11: As Figure S10, but considering a 20-year time-horizon.

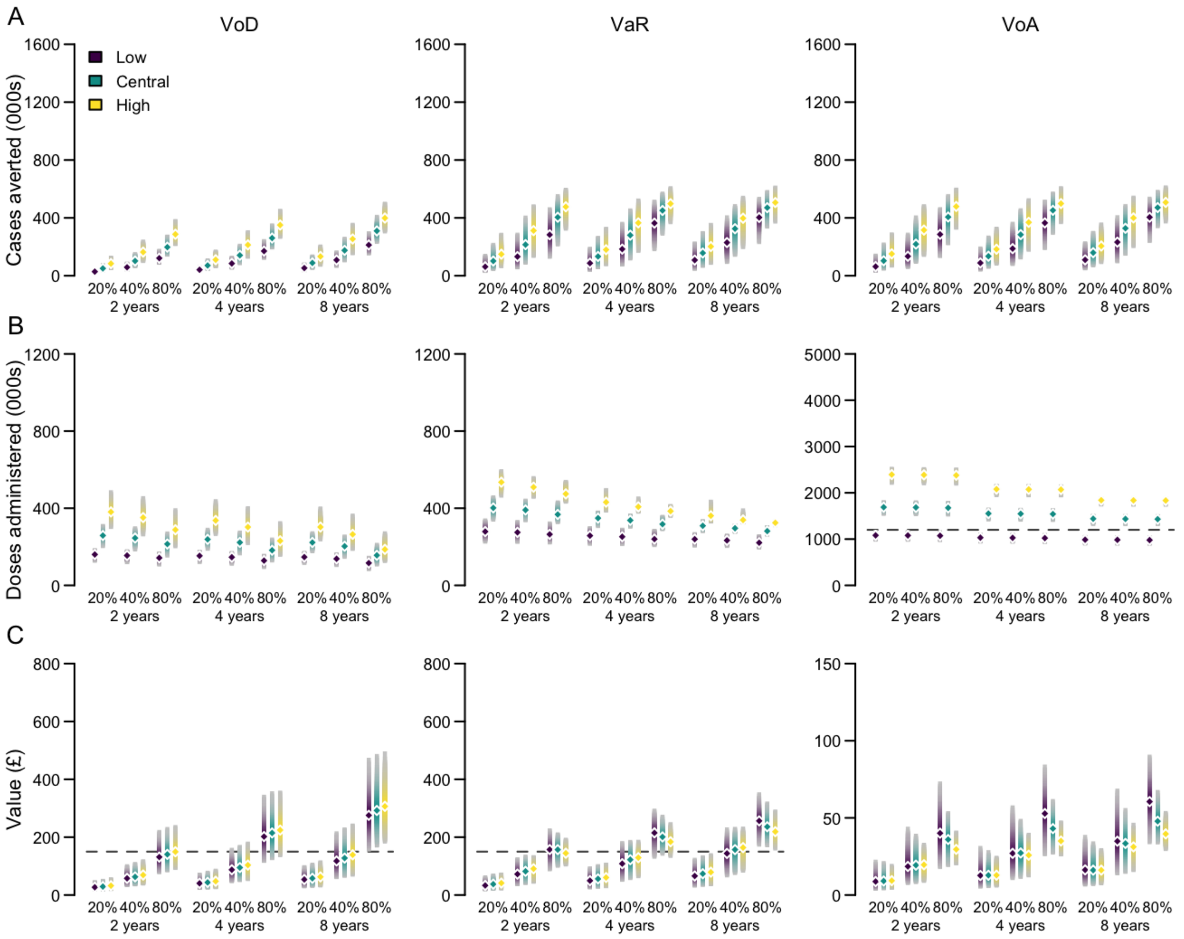

Figure S12: As Figure S10, but using the upper-bound baseline which assumes that behavioural trends continue.

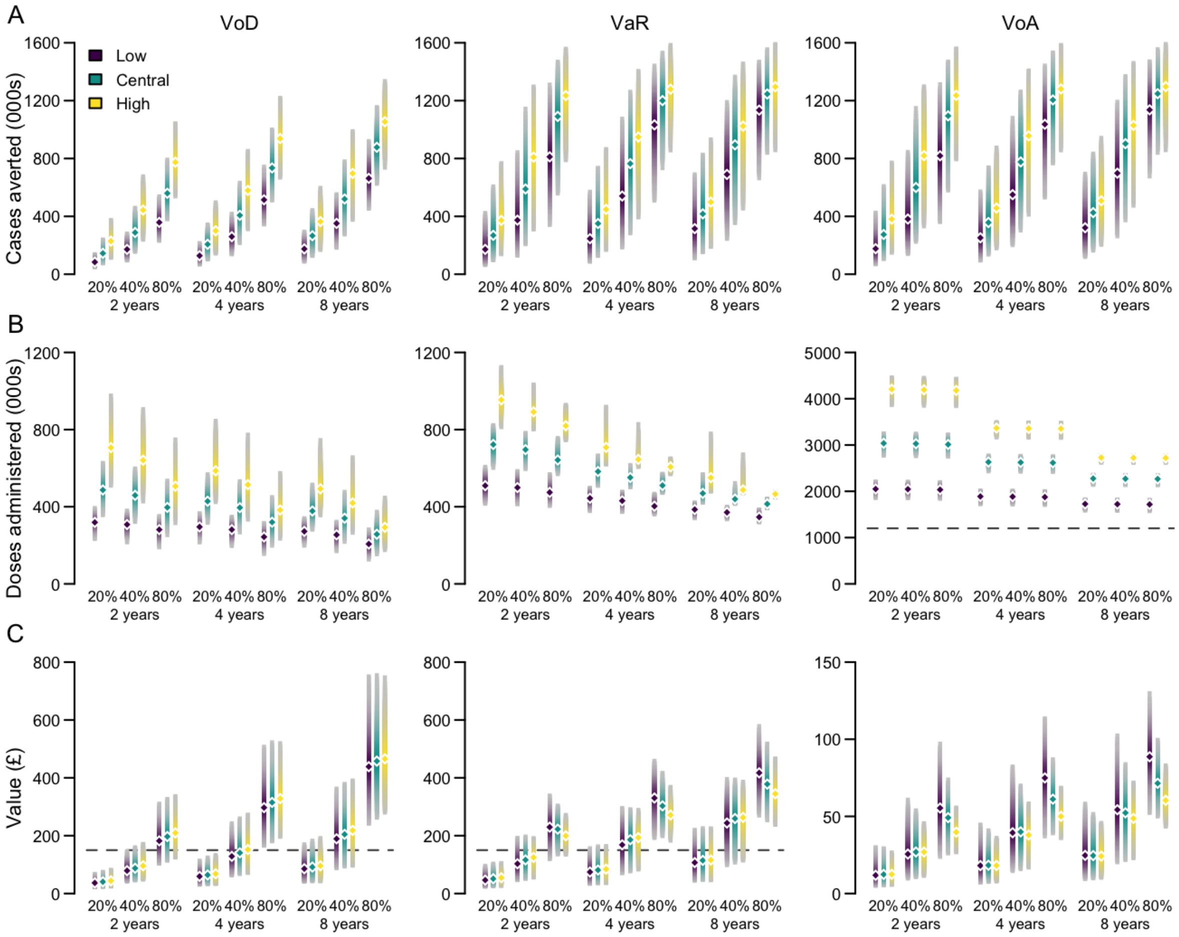

Figure S13: As Figure S12 but considering a 20-year time-horizon.

2.3 Effects of vaccine efficacy and duration of protection

Figure 4 in the main paper considers vaccination over a 10-year time-horizon against the lower-bound baseline which assumes that behavioural trends stabilise. This is reproduced here as Figure S14 for comparison with other figures, which present the results of considering a 20-year time-horizon (Figures S15, S17), and the results when using the upper-bound baseline which assumes that behavioural trends continue, considering 10- and 20-year time-horizons (Figures S16, S17). The value of vaccination is greater when assessed over the longer time-horizon, and when the upper-bound behavioural baseline is used; it can be seen that the lines in panels (B) and (C) are steeper in Figures S16, S17 than in the corresponding Figures S14, S15, with "diminishing returns" of increased efficacy or duration of protection being less pronounced.

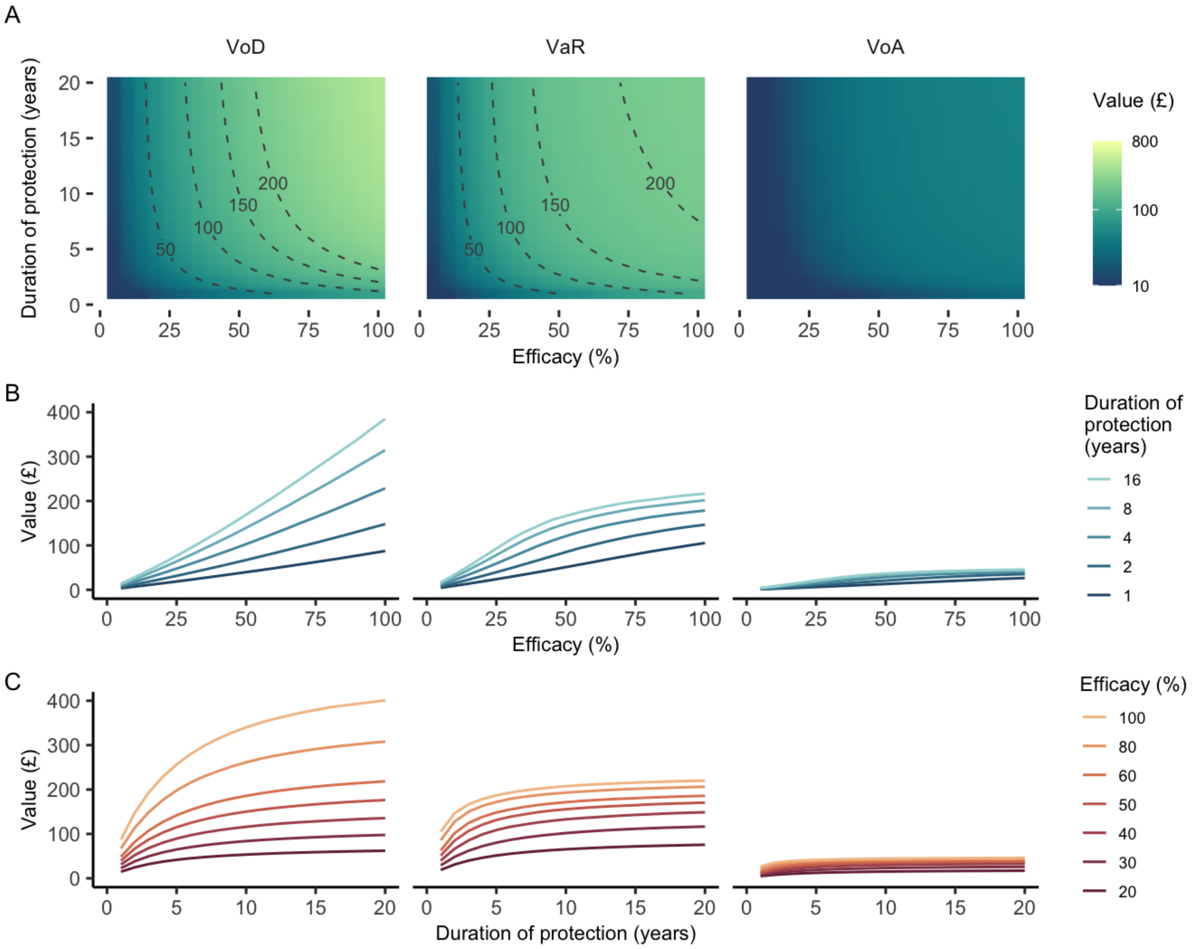

Figure S14: Value per dose of vaccination of MSM in England against gonorrhoea over 10 years under three targeting strategies. Sections show (A) Heatmaps of the median value per dose administered of vaccines providing 1–100% protection against infection for 1–20 years, with contour lines showing values of £50, £100, £150, £200; (B) Impact of duration of protection on the median value per dose administered of vaccines with a range of efficacies; (C) Impact of efficacy on the median value per dose administered of vaccines with a range of durations of protection. The lines plotted in (B) and (C) are transects through the heatmaps in (A), with value represented in the vertical axis rather than by colour. Simulations compare each vaccination strategy against no vaccination, using the lower-bound baseline which assumes that behavioural trends stabilise, using 1,000 sets of sampled epidemiological and health-economic parameters, vaccine uptake of 33·0%(95%CI:32·7%–33·3%), and a QALY valued at £20,000.

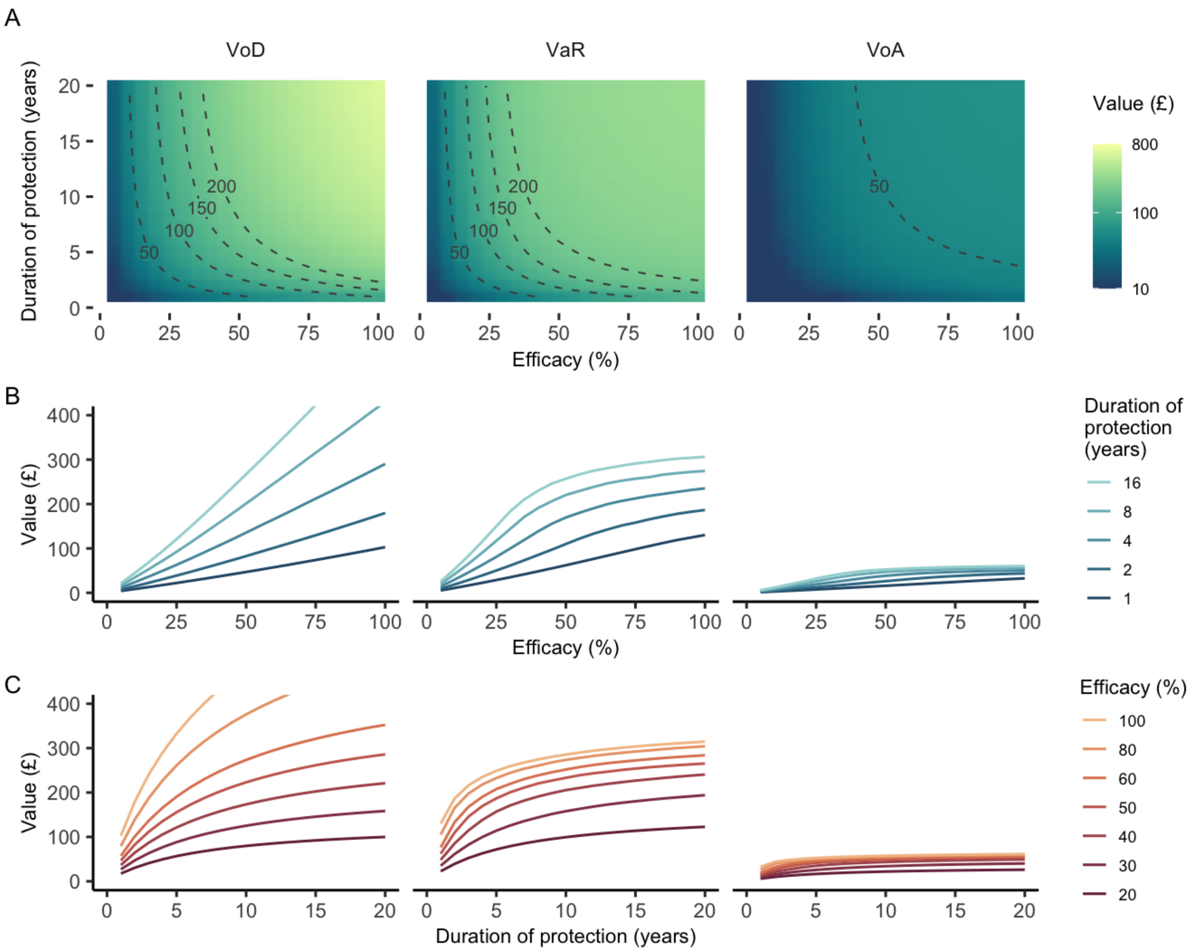

Figure S15: As Figure S14, but considering a 20-year time-horizon.

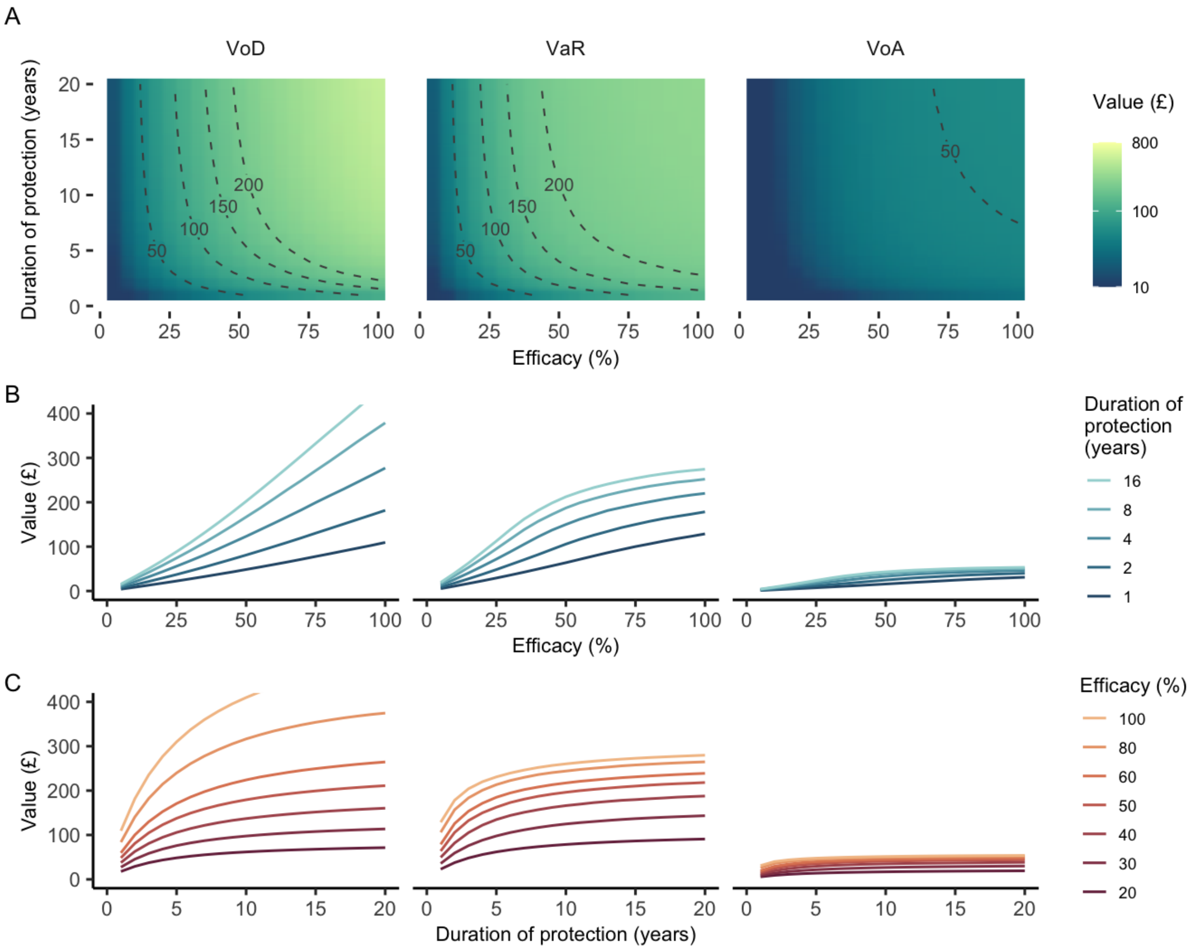

Figure S16: As Figure S14, but using the upper-bound baseline which assumes that behavioural trends continue.

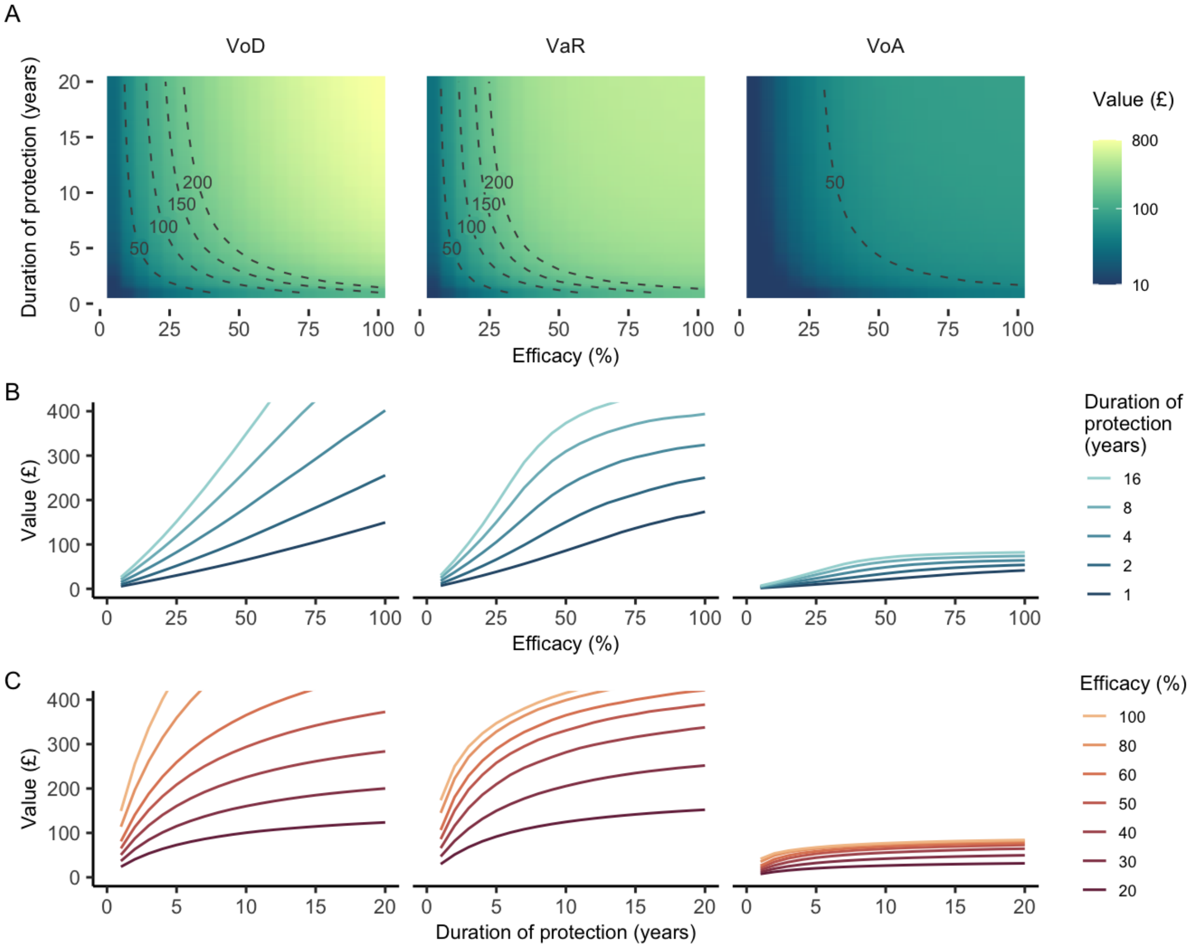

Figure S17: As Figure S16 but considering a 20-year time-horizon.

2.4 Analysis of 4CMenB

2.4.1 Value per dose

Figure 5A&B in the main paper considers the impact and value of vaccination with 4CMenB over a 10-year time-horizon against the lower-bound baseline which assumes that behavioural trends stabilise. Figure 5A&B is reproduced here (with some additional durations of protection considered) as Figure S18, for comparison with other figures, which present the results of considering a 20-year time-horizon (Figures S19, S21), and results when using the upper-bound baseline which assumes that behavioural trends continue, considering 10- and 20-year time-horizons (Figures S20, S21). These also show results with the value of a QALY increased to £30,000; this produces a modest increase in the value of vaccination because the majority of the estimated benefit of vaccination is averting costs rather than gaining QALYs. The value of vaccination is greater when assessed over the longer time-horizon, and when the upper-bound behavioural baseline is used; it can be seen that the probability of being cost-effective at a given price is higher in Figures S20, S21 than in the corresponding Figures S18, S19.

The estimated values of vaccination under the three targeting strategies for vaccines with efficacies and durations of protection considered in Figures S18-S21, considering 10- and 20-year time-horizons, and using lower- and upper-bound behavioural baselines are shown in Tables S6-S9. JCVI's Code of Practice recommends that the probability of the cost per QALY gained exceeding £30,000 should be no more than 10% for an intervention to be recommended [31], so in Tables S10-S13 we show costs per dose corresponding to 90% probability of vaccination being cost-effective with a QALY valued at £20,000 or £30,000.

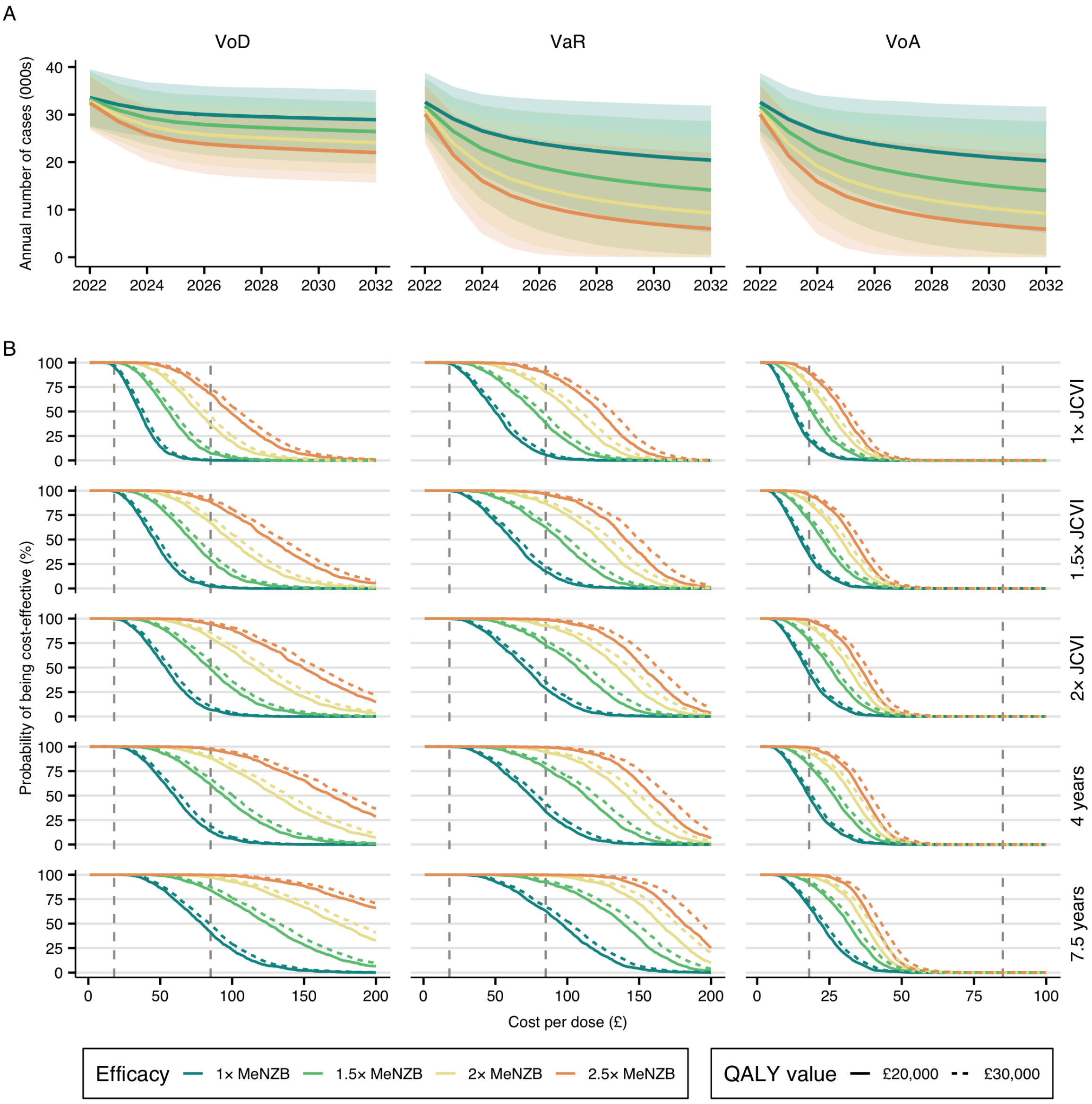

Figure S18: Impact of 4CMenB vaccination over 10 years and how the cost per dose affects the probability of vaccination being cost-effective. Section (A) shows cases of gonorrhoea in MSM in England following introduction of vaccination in 2022 under different targeting strategies using 4CMenB if it is 1×/1·5×/2×/2·5× as protective as MeNZB (31%, 95%CI:21%–39%), with durations of protection as estimated by JCVI for protection of infants against serogroup B meningococcal disease (18 & 36 months after primary vaccination and revaccination, respectively); lines show medians and shading shows 95%CrI. Section (B) shows the probability that vaccination is cost-effective (i.e. its value exceeds its cost) at different costs per dose administered, with a QALY valued at £20,000 (solid lines) or £30,000 (dashed lines); if 4CMenB is 1×/1·5×/2×/2·5× as protective as MeNZB, indicated by colour; with a duration of protection that is as estimated by JCVI, or 4 years after primary vaccination and revaccination, or 7·5 years after primary vaccination and revaccination, as indicated by the labelling of the rows of panels. Columns of panels show the different targeting strategies (note the different horizontal scale for VoA). Vertical dashed lines show two alternative costs per dose of 4CMenB administered: £18, corresponding to the estimated NHS price of £8 per dose plus the £10 administration cost; or £85, corresponding to the current UK list price of £75 plus the £10 administration cost. Simulations compare each vaccination strategy (with uptake of 33·0%(95%CI:32·7%–33·3%)) against no vaccination, using the lower-bound baseline which assumes that behavioural trends stabilise, using 1,000 sets of sampled epidemiological and health-economic parameters.

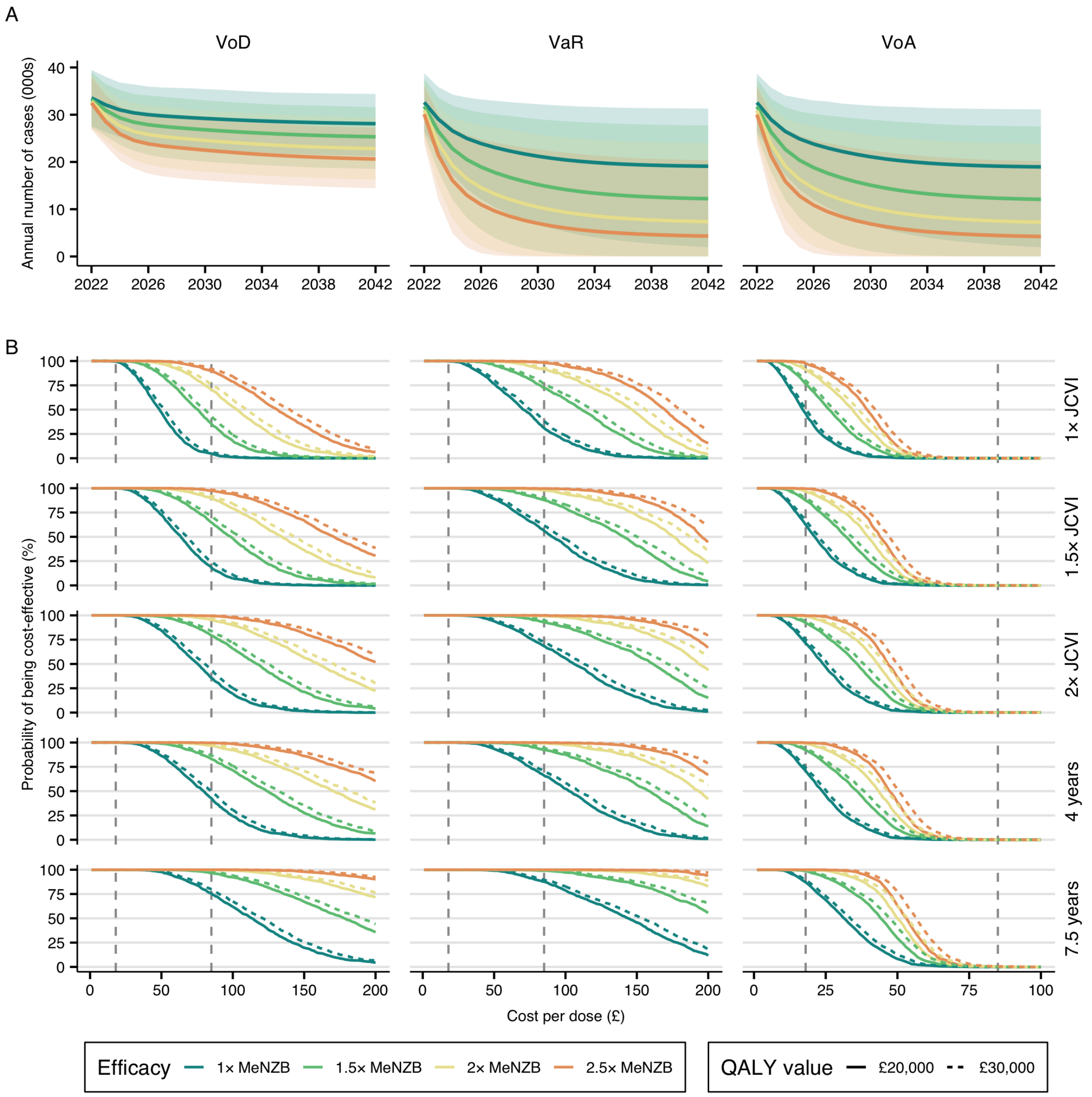

Figure S19: As Figure S18, but considering a 20-year time-horizon.

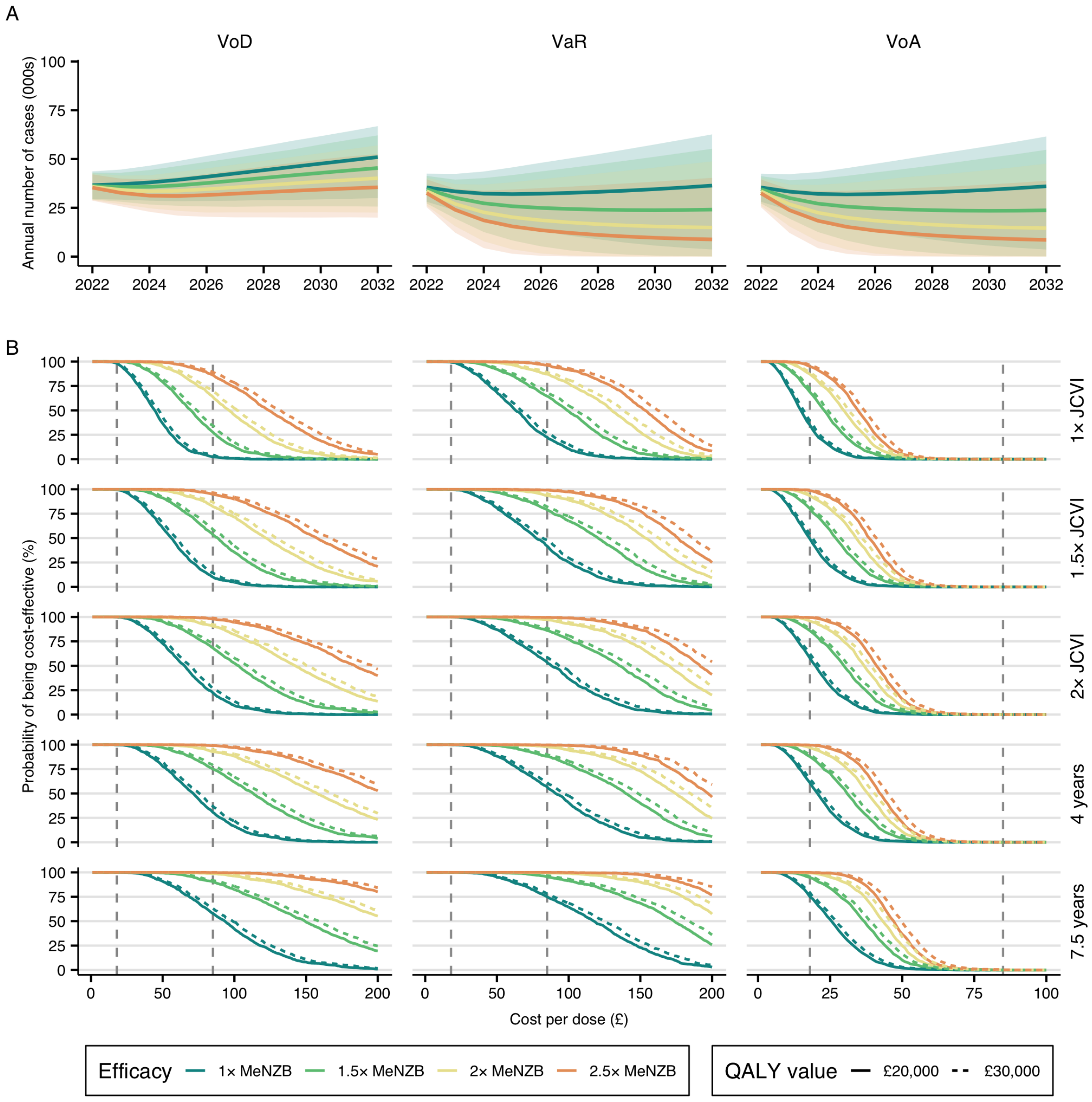

Figure S20: As Figure S18, but using the upper-bound baseline which assumes that behavioural trends continue.

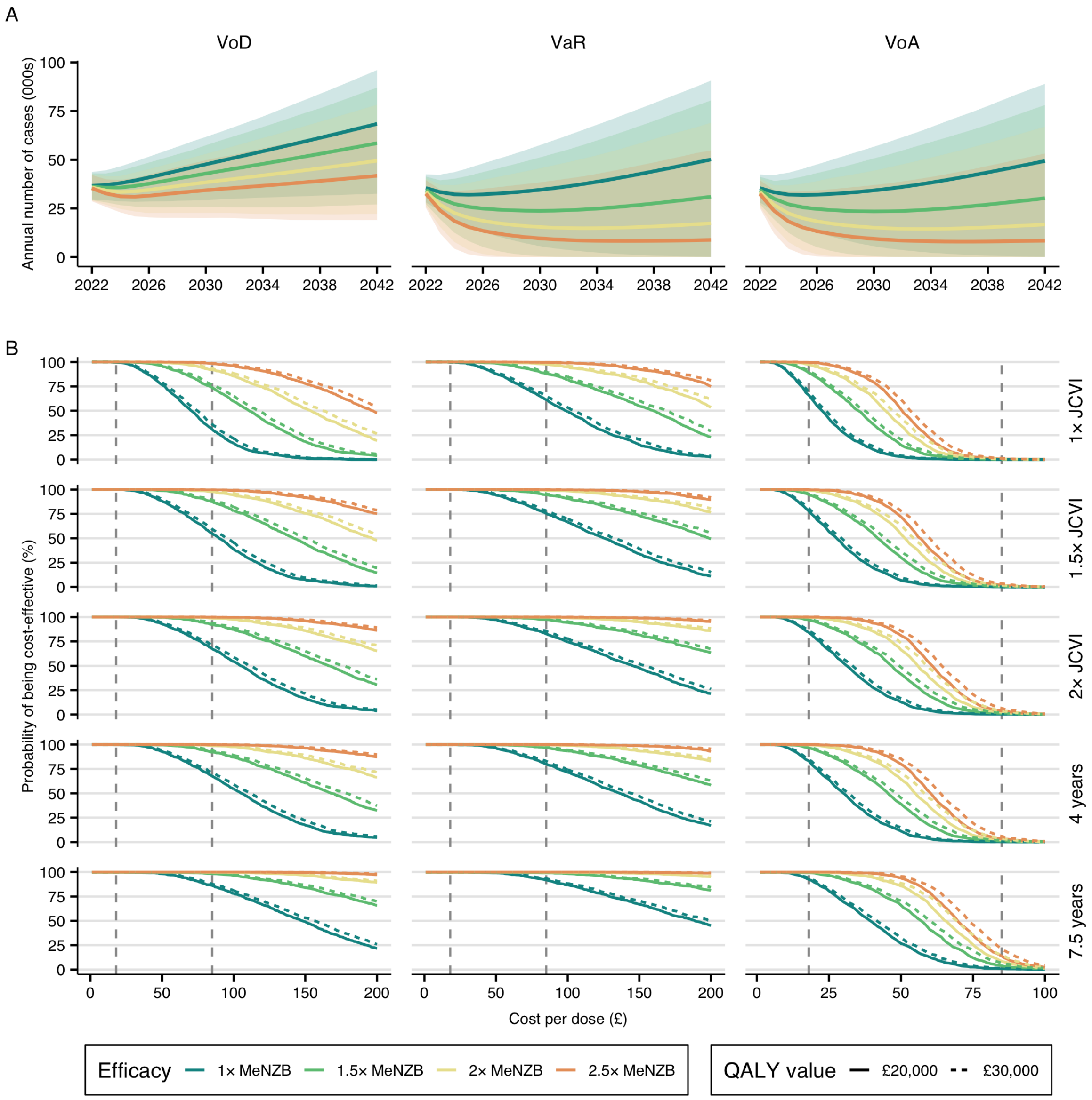

Figure S21: As Figure S20, but considering a 20-year time-horizon.

Table S6: Value per dose administered of vaccination using 4CMenB using different targeting strategies with uptake of 33·0%(95%CI:32·7%–33·3%), under different assumptions of efficacy and duration of protection, using the lower-bound baseline which assumes that behavioural trends stabilise, with a QALY valued at £20,000, and considering a 10-year time-horizon.

**Duration of protection**	**Strategy**	**Value of vaccination** Median (95%CrI)			
		**Vaccine efficacy** Mean (95%CI)			
Primary vaccination and revaccination		1× MeNZB 31%(21%–41%)	1·5× MeNZB 46%(32%–61%)	2× MeNZB 62%(43%–81%)	2·5× MeNZB 77%(54%–100%)
1× JCVI	VoD	£35·86 (£16·62-£65·33)	£55·66 (£26·22-£99·34)	£76·48 (£36·71-£134·76)	£98·20 (£48·07-£168·08)
18 & 36 months	VaR	£49·79 (£20·46-£92·71)	£77·18 (£33·42-£127·67)	£102·33 (£48·50-£150·43)	£122·80 (£64·57-£167·87)
	VoA	£12·34 (£4·38-£28·11)	£18·84 (£7·00-£36·59)	£24·98 (£9·96-£42·89)	£29·54 (£13·36-£46·03)
1·5× JCVI	VoD	£45·95 (£21·25-£83·66)	£71·42 (£33·68-£127·31)	£98·40 (£47·32-£173·65)	£126·47 (£62·11-£216·36)
27 & 54 months	VaR	£61·37 (£25·52-£112·16)	£95·01 (£42·15-£146·35)	£121·92 (£61·53-£170·18)	£140·64 (£81·44-£190·92)
	VoA	£14·95 (£5·42-£32·09)	£22·61 (£8·67-£40·90)	£28·92 (£12·36-£46·38)	£33·36 (£16·61-£49·47)
2× JCVI	VoD	£53·82 (£24·85-£98·10)	£84·02 (£39·57-£149·54)	£116·03 (£55·75-£204·38)	£149·38 (£73·31-£255·53)
36 & 72 months	VaR	£70·58 (£29·46-£125·48)	£106·81 (£48·60-£159·54)	£134·95 (£70·87-£184·89)	£152·72 (£93·62-£205·27)
	VoA	£16·88 (£6·22-£34·70)	£25·24 (£9·96-£43·74)	£31·38 (£14·17-£48·33)	£35·63 (£18·90-£51·23)
4 years	VoD	£59·94 (£27·70-£110·03)	£94·26 (£44·28-£169·43)	£130·39 (£62·69-£231·37)	£169·18 (£82·81-£293·66)
48 & 48 months	VaR	£74·42 (£31·54-£130·67)	£112·44 (£52·55-£165·78)	£141·23 (£76·21-£193·01)	£158·57 (£100·88-£211·30)
	VoA	£17·77 (£6·63-£35·78)	£26·57 (£10·68-£45·01)	£32·81 (£15·22-£49·48)	£36·82 (£20·31-£52·28)
7·5 years	VoD	£78·62 (£36·24-£145·23)	£124·76 (£58·33-£224·88)	£173·93 (£83·19-£309·51)	£226·39 (£110·44-£394·03)
90 & 90 months	VaR	£95·98 (£41·19-£155·55)	£138·62 (£69·05-£193·28)	£164·59 (£100·20-£221·45)	£181·12 (£125·86-£237·70)
	VoA	£22·06 (£8·50-£40·60)	£31·44 (£13·77-£49·43)	£37·16 (£19·61-£53·32)	£40·35 (£25·19-£56·29)

Table S7: As Table S6, but considering a 20-year time-horizon.

**Duration of protection**	**Strategy**	**Value of vaccination** Median (95%CrI)			
		**Vaccine efficacy** Mean (95%CI)			
Primary vaccination and revaccination		1× MeNZB 31%(21%–41%)	1·5× MeNZB 46%(32%–61%)	2× MeNZB 62%(43%–81%)	2·5× MeNZB 77%(54%–100%)
1× JCVI	VoD	£48·73 (£22·11-£90·75)	£75·48 (£34·93-£136·24)	£102·91 (£48·64-£181·60)	£131·17 (£64·19-£224·19)
18 & 36 months	VaR	£70·63 (£27·76-£134·77)	£111·38 (£45·93-£177·99)	£146·61 (£67·01-£206·79)	£169·59 (£90·20-£230·03)
	VoA	£17·02 (£5·92-£39·06)	£26·13 (£9·51-£48·70)	£34·30 (£13·59-£55·08)	£39·35 (£18·14-£58·33)
1·5× JCVI	VoD	£63·52 (£28·78-£118·28)	£98·44 (£45·55-£177·06)	£134·46 (£63·79-£235·43)	£170·82 (£83·60-£290·71)
27 & 54 months	VaR	£90·56 (£35·67-£167·15)	£140·83 (£59·51-£209·99)	£175·76 (£87·24-£239·84)	£195·46 (£118·06-£261·23)
	VoA	£21·12 (£7·54-£44·43)	£32·17 (£12·12-£54·65)	£39·69 (£17·32-£58·73)	£43·97 (£23·01-£62·05)
2× JCVI	VoD	£75·52 (£34·08-£140·33)	£116·90 (£54·22-£210·17)	£159·81 (£76·07-£278·68)	£202·80 (£99·36-£344·96)
36 & 72 months	VaR	£105·91 (£41·91-£188·29)	£161·17 (£70·02-£230·91)	£193·68 (£103·19-£263·33)	£214·06 (£138·63-£281·05)
	VoA	£24·07 (£8·76-£47·40)	£35·97 (£14·16-£58·34)	£43·09 (£20·19-£62·35)	£46·62 (£26·67-£64·57)
4 years	VoD	£79·73 (£35·84-£149·20)	£124·04 (£56·82-£226·91)	£170·43 (£81·05-£304·83)	£218·29 (£106·44-£378·60)
48 & 48 months	VaR	£102·82 (£41·75-£184·30)	£158·96 (£69·27-£226·41)	£193·34 (£102·70-£263·05)	£213·96 (£137·73-£283·21)
	VoA	£23·71 (£8·69-£47·65)	£35·91 (£14·09-£58·77)	£43·50 (£20·17-£62·74)	£47·20 (£26·63-£64·90)
7·5 years	VoD	£113·71 (£50·89-£213·21)	£178·09 (£81·49-£325·05)	£245·56 (£116·87-£437·03)	£313·89 (£153·71-£544·99)
90 & 90 months	VaR	£144·16 (£58·73-£231·35)	£206·23 (£98·75-£282·54)	£235·83 (£147·99-£307·80)	£254·35 (£185·47-£334·68)
	VoA	£31·44 (£11·98-£56·43)	£44·11 (£19·60-£65·18)	£49·97 (£27·92-£68·90)	£53·04 (£35·60-£70·83)

Table S8: As Table S6, but using the upper-bound baseline which assumes that behavioural trends continue.

**Duration of protection**	**Strategy**	**Value of vaccination** Median (95%CrI)			
		**Vaccine efficacy** Mean (95%CI)			
Primary vaccination and revaccination		1× MeNZB 31%(21%–41%)	1·5× MeNZB 46%(32%–61%)	2× MeNZB 62%(43%–81%)	2·5× MeNZB 77%(54%–100%)
1× JCVI	VoD	£44·61 (£18·67-£84·51)	£69·74 (£30·77-£129·52)	£96·44 (£44·62-£174·27)	£124·04 (£59·11-£214·13)
18 & 36 months	VaR	£62·96 (£23·05-£123·40)	£99·00 (£38·66-£164·51)	£129·93 (£56·65-£194·95)	£153·28 (£78·46-£219·11)
	VoA	£14·91 (£4·87-£33·41)	£22·89 (£8·10-£44·28)	£29·77 (£11·96-£50·38)	£34·71 (£16·09-£53·31)
1·5× JCVI	VoD	£56·07 (£23·40-£106·88)	£88·39 (£38·97-£163·50)	£122·16 (£56·48-£220·89)	£157·58 (£74·86-£269·92)
27 & 54 months	VaR	£77·25 (£28·62-£147·29)	£120·61 (£47·73-£191·47)	£153·55 (£70·95-£224·25)	£176·11 (£97·41-£248·47)
	VoA	£17·83 (£5·93-£37·51)	£26·98 (£9·83-£49·67)	£34·09 (£14·71-£54·09)	£38·66 (£19·61-£57·18)
2× JCVI	VoD	£65·11 (£26·99-£124·41)	£103·11 (£45·05-£189·77)	£142·65 (£65·73-£257·69)	£184·03 (£87·19-£316·50)
36 & 72 months	VaR	£88·07 (£32·67-£163·12)	£135·37 (£54·65-£210·77)	£170·03 (£81·92-£242·19)	£191·67 (£112·52-£267·49)
	VoA	£19·89 (£6·73-£40·68)	£29·77 (£11·19-£52·24)	£36·82 (£16·70-£56·61)	£41·10 (£22·16-£59·84)
4 years	VoD	£71·38 (£29·21-£137·13)	£113·18 (£49·16-£211·16)	£157·75 (£71·57-£286·85)	£204·75 (£95·93-£358·21)
48 & 48 months	VaR	£90·87 (£34·05-£166·86)	£139·83 (£57·34-£214·60)	£174·51 (£86·66-£251·44)	£197·35 (£119·50-£274·45)
	VoA	£20·67 (£7·17-£41·59)	£30·83 (£11·89-£53·07)	£38·06 (£17·65-£57·42)	£42·27 (£23·44-£61·05)
7·5 years	VoD	£93·47 (£37·56-£180·35)	£149·18 (£64·22-£280·25)	£209·60 (£94·42-£384·05)	£273·32 (£126·80-£489·55)
90 & 90 months	VaR	£117·87 (£43·79-£202·92)	£173·51 (£75·44-£258·27)	£206·86 (£114·49-£292·90)	£228·03 (£150·84-£313·55)
	VoA	£25·55 (£9·11-£48·40)	£36·59 (£15·25-£58·37)	£43·27 (£22·53-£63·15)	£47·03 (£29·04-£66·51)

Table S9: As Table S8, but considering a 20-year time-horizon.

**Duration of protection**	**Strategy**	**Value of vaccination** Median (95%CrI)			
		**Vaccine efficacy** Mean (95%CI)			
Primary vaccination and revaccination		1× MeNZB 31%(21%–41%)	1·5× MeNZB 46%(32%–61%)	2× MeNZB 62%(43%–81%)	2·5× MeNZB 77%(54%–100%)
1× JCVI 18 & 36 months	VoD	£70·37 (£26·95-£140·06)	£110·65 (£45·61-£212·08)	£153·81 (£67·36-£278·48)	£197·11 (£90·99-£338·73)
	VaR	£98·72 (£32·71-£199·21)	£159·89 (£55·64-£264·78)	£205·76 (£87·31-£303·36)	£235·06 (£120·84-£340·02)
	VoA	£22·67 (£7·17-£51·06)	£35·22 (£11·69-£65·46)	£44·61 (£17·94-£70·89)	£50·39 (£24·54-£75·50)
1·5× JCVI 27 & 54 months	VoD	£89·75 (£33·97-£179·20)	£141·98 (£58·38-£269·56)	£196·64 (£86·68-£352·09)	£250·97 (£116·94-£427·29)
	VaR	£125·59 (£41·11-£246·13)	£198·48 (£71·27-£316·71)	£246·84 (£110·18-£358·06)	£278·64 (£153·12-£390·96)
	VoA	£27·79 (£8·91-£57·78)	£41·98 (£14·60-£72·62)	£51·38 (£22·56-£77·95)	£56·43 (£30·67-£83·43)
2× JCVI 36 & 72 months	VoD	£104·96 (£39·46-£208·89)	£166·43 (£68·04-£314·39)	£230·51 (£101·69-£410·35)	£293·16 (£137·35-£497·07)
	VaR	£145·63 (£47·65-£276·58)	£226·21 (£83·28-£351·32)	£275·51 (£129·78-£399·32)	£306·39 (£177·14-£427·55)
	VoA	£31·35 (£10·20-£62·91)	£46·68 (£16·78-£77·20)	£55·69 (£26·04-£84·09)	£60·94 (£34·96-£88·17)
4 years 48 & 48 months	VoD	£105·43 (£39·24-£211·49)	£168·35 (£67·75-£321·07)	£234·90 (£102·46-£430·09)	£302·61 (£139·80-£529·32)
	VaR	£136·35 (£45·83-£264·06)	£214·64 (£79·82-£338·35)	£266·24 (£123·87-£386·41)	£297·00 (£168·19-£419·79)
	VoA	£30·14 (£9·87-£62·00)	£45·53 (£16·38-£76·37)	£54·94 (£24·97-£83·52)	£60·41 (£33·90-£87·89)
7·5 years 90 & 90 months	VoD	£147·99 (£53·82-£298·50)	£237·65 (£94·30-£449·88)	£332·01 (£144·72-£601·38)	£425·34 (£198·18-£745·84)
	VaR	£188·73 (£63·27-£341·81)	£285·55 (£112·63-£424·61)	£336·72 (£175·20-£476·88)	£364·55 (£228·37-£509·54)
	VoA	£39·26 (£13·28-£75·82)	£57·04 (£22·34-£87·71)	£65·37 (£33·98-£96·58)	£69·74 (£43·75-£99·37)

Table S10: Cost per dose (including £10 administration fee) corresponding to a 90% probability of 4CMenB being cost-effective using different targeting strategies with uptake of 33·0%(95%CI:32·7%–33·3%), under different assumptions of efficacy and duration of protection, using the lower-bound baseline which assumes that behavioural trends stabilise, with a QALY valued at £20,000 or £30,000, and considering a 10-year time-horizon.

**Duration of protection**	**Strategy**	**Cost per dose** with a QALY valued at £20,000; or £30,000			
		**Vaccine efficacy** Mean (95%CI)			
Primary vaccination and revaccination		1× MeNZB 31%(21%–41%)	1·5× MeNZB 46%(32%–61%)	2× MeNZB 62%(43%–81%)	2·5× MeNZB 77%(54%–100%)
1× JCVI	VoD	£21·31; £22·88	£34·04; £36·45	£48·26; £51·38	£63·59; £68·14
18 & 36 months	VaR	£27·32; £29·27	£43·99; £46·94	£63·28; £67·74	£83·81; £89·55
	VoA	£6·11; £6·51	£9·71; £10·31	£13·62; £14·58	£17·66; £18·75
1·5× JCVI	VoD	£27·17; £29·26	£43·71; £46·70	£61·73; £66·18	£81·93; £87·98
27 & 54 months	VaR	£34·07; £36·52	£55·48; £59·17	£79·73; £85·47	£104·09; £111·60
	VoA	£7·55; £8·04	£12·01; £12·84	£16·84; £17·93	£21·48; £23·00
2× JCVI	VoD	£31·83; £34·27	£51·33; £54·77	£72·49; £77·89	£96·62; £103·75
36 & 72 months	VaR	£39·22; £41·98	£64·12; £68·60	£91·65; £98·07	£116·60; £124·36
	VoA	£8·61; £9·21	£13·74; £14·65	£19·18; £20·33	£24·29; £25·77
4 years	VoD	£35·41; £38·18	£57·33; £61·14	£81·50; £86·95	£108·70; £116·07
48 & 48 months	VaR	£42·08; £44·83	£68·67; £73·56	£98·84; £104·98	£123·32; £131·46
	VoA	£9·20; £9·85	£14·71; £15·72	£20·49; £21·73	£25·87; £27·60
7·5 years	VoD	£46·44; £49·94	£75·45; £80·44	£108·11; £115·90	£145·43; £154·22
90 & 90 months	VaR	£55·00; £58·37	£90·72; £96·80	£125·10; £133·14	£147·46; £156·95
	VoA	£11·73; £12·59	£18·77; £20·03	£25·44; £27·07	£30·71; £32·88

Table S11: As Table S10, but considering a 20-year time-horizon.

**Duration of protection**	**Strategy**	**Cost per dose** with a QALY valued at £20,000; or £30,000			
		**Vaccine efficacy** Mean (95%CI)			
Primary vaccination and revaccination		1× MeNZB 31%(21%–41%)	1·5× MeNZB 46%(32%–61%)	2× MeNZB 62%(43%–81%)	2·5× MeNZB 77%(54%–100%)
1× JCVI	VoD	£28·40; £30·34	£45·32; £48·63	£64·17; £68·53	£84·18; £89·65
18 & 36 months	VaR	£37·76; £40·23	£61·12; £65·36	£88·43; £94·99	£117·86; £126·39
	VoA	£8·36; £8·93	£13·19; £14·14	£18·63; £19·96	£24·25; £25·76
1·5× JCVI	VoD	£37·00; £39·58	£59·12; £63·63	£84·24; £89·47	£110·73; £117·77
27 & 54 months	VaR	£48·54; £51·73	£79·20; £84·82	£115·00; £123·42	£150·63; £160·23
	VoA	£10·55; £11·26	£16·77; £17·94	£23·56; £25·16	£30·13; £32·04
2× JCVI	VoD	£43·93; £47·02	£70·34; £75·73	£100·19; £106·43	£131·84; £140·67
36 & 72 months	VaR	£56·84; £60·51	£93·58; £99·89	£135·57; £145·62	£167·75; £179·66
	VoA	£12·22; £13·03	£19·51; £20·82	£27·30; £28·97	£34·06; £36·33
4 years	VoD	£45·98; £49·57	£73·99; £79·82	£105·83; £112·91	£140·91; £149·46
48 & 48 months	VaR	£55·68; £59·54	£92·12; £98·40	£134·38; £143·84	£167·30; £177·68
	VoA	£12·11; £12·89	£19·44; £20·65	£27·10; £28·96	£34·12; £36·32
7·5 years	VoD	£65·69; £70·32	£106·61; £114·27	£152·39; £162·38	£203·40; £215·47
90 & 90 months	VaR	£78·55; £83·81	£132·56; £141·16	£183·82; £196·87	£209·84; £224·00
	VoA	£16·50; £17·57	£26·66; £28·55	£36·10; £38·59	£42·28; £45·31

Table S12: As Table S10, but using the upper-bound baseline which assumes that behavioural trends continue.

**Duration of protection**	**Strategy**	**Cost per dose** with a QALY valued at £20,000; or £30,000			
		**Vaccine efficacy** Mean (95%CI)			
Primary vaccination and revaccination		1× MeNZB 31%(21%–41%)	1·5× MeNZB 46%(32%–61%)	2× MeNZB 62%(43%–81%)	2·5× MeNZB 77%(54%–100%)
1× JCVI	VoD	£24·95; £26·56	£40·89; £43·43	£58·87; £62·49	£78·62; £83·50
18 & 36 months	VaR	£32·16; £33·86	£53·20; £56·33	£78·14; £82·93	£106·12; £112·15
	VoA	£7·09; £7·56	£11·65; £12·27	£16·39; £17·31	£21·47; £22·70
1·5× JCVI	VoD	£31·24; £33·15	£51·53; £54·74	£74·65; £79·25	£99·72; £105·89
27 & 54 months	VaR	£39·56; £41·66	£66·25; £70·06	£98·12; £103·56	£126·72; £135·17
	VoA	£8·64; £9·13	£14·12; £14·88	£19·84; £21·06	£25·82; £27·38
2× JCVI	VoD	£36·08; £38·18	£59·97; £63·53	£86·65; £92·26	£116·35; £123·48
36 & 72 months	VaR	£45·07; £47·68	£75·86; £80·52	£112·74; £119·63	£141·43; £149·64
	VoA	£9·80; £10·33	£15·96; £16·83	£22·39; £23·68	£28·86; £30·78
4 years	VoD	£39·41; £41·34	£65·45; £69·34	£94·98; £101·23	£128·70; £136·04
48 & 48 months	VaR	£47·09; £49·91	£80·00; £84·76	£118·42; £125·17	£147·68; £156·07
	VoA	£10·30; £10·82	£16·62; £17·67	£23·56; £25·03	£30·22; £32·10
7·5 years	VoD	£50·98; £53·72	£85·58; £90·82	£125·44; £133·34	£170·83; £180·65
90 & 90 months	VaR	£61·03; £64·81	£104·63; £111·30	£150·13; £159·14	£177·78; £189·15
	VoA	£13·03; £13·69	£21·13; £22·47	£29·73; £31·70	£35·74; £37·88

Table S13: As Table S12, but considering a 20-year time-horizon.

**Duration of protection**	**Strategy**	**Cost per dose** with a QALY valued at £20,000; or £30,000			
		**Vaccine efficacy** Mean (95%CI)			
Primary vaccination and revaccination		1× MeNZB 31%(21%–41%)	1·5× MeNZB 46%(32%–61%)	2× MeNZB 62%(43%–81%)	2·5× MeNZB 77%(54%–100%)
1× JCVI 18 & 36 months	VoD	£37·01; £38·99	£62·13; £65·73	£90·76; £96·12	£122·86; £130·17
	VaR	£46·79; £49·48	£80·36; £84·97	£119·93; £126·96	£162·68; £172·97
	VoA	£10·70; £11·27	£17·61; £18·57	£25·15; £26·85	£33·23; £34·95
1·5× JCVI 27 & 54 months	VoD	£47·07; £49·45	£79·45; £84·07	£117·11; £123·52	£158·36; £167·36
	VaR	£59·19; £62·66	£102·54; £108·02	£155·28; £163·37	£197·91; £209·94
	VoA	£13·25; £13·98	£21·80; £23·05	£31·54; £33·50	£40·04; £42·39
2× JCVI 36 & 72 months	VoD	£54·98; £57·66	£93·05; £98·42	£137·24; £144·82	£185·72; £196·25
	VaR	£68·39; £72·37	£119·47; £126·14	£180·94; £191·76	£224·37; £236·50
	VoA	£15·15; £16·01	£24·99; £26·52	£35·94; £38·06	£44·72; £47·47
4 years 48 & 48 months	VoD	£55·03; £57·75	£92·81; £98·08	£137·34; £145·75	£187·36; £199·06
	VaR	£65·39; £69·00	£113·19; £119·31	£171·41; £181·80	£214·77; £228·14
	VoA	£14·57; £15·41	£24·11; £25·51	£34·85; £36·89	£43·49; £46·01
7·5 years 90 & 90 months	VoD	£75·83; £79·91	£131·14; £138·52	£195·14; £207·97	£265·83; £281·94
	VaR	£90·07; £95·31	£159·55; £167·87	£234·04; £245·13	£276·70; £293·99
	VoA	£19·61; £20·77	£32·55; £34·76	£45·50; £47·95	£53·41; £56·70

2.4.2 Comparison of vaccine targeting strategies

As can be seen from Figures 2, 3, S6-S13, vaccination averts infections, so all vaccination strategies gain QALYs and reduce costs of care compared with no vaccination. If the vaccine price is sufficiently low then vaccination will be net cost-saving, or if the vaccine price is high enough that the cost of vaccination exceeds the costs of care averted then there will be a net cost – and of course the threshold price is different for different strategies.

Targeting strategies using 4CMenB, under conservative assumptions about efficacy and duration of protection, are compared incrementally, using cost-effectiveness efficiency frontiers (Figures S22-S25) with corresponding tables (Tables S14-S17). (Note that Figure S22 is a reproduction of Figure 5C.) Two alternative costs per dose of 4CMenB administered are assumed: £18, corresponding to the estimated NHS price of £8 per dose plus the £10 administration cost; or £85, corresponding to the current UK list price of £75 plus the £10 administration cost. The lower- and upper-bound baselines are used, and 10- and 20-year time-horizons are considered.

Initially comparing each strategy vs no vaccination, in all comparisons VoD gains the fewest QALYs, VoA gains the most, and VaR gains almost as many as VoA (Figures S22-S25); VoD uses the fewest vaccine doses, VoA uses the most ($> 6\times$ VoD), and VaR uses approximately 45%–75% more than VoD (Tables S14-S17).

If vaccination costs £18 per dose administered then VoD and VaR are cost-saving in all cases (Figures S22-S25), whilst VoA has a net cost (Figures S22-S24) except when using the upper-bound baseline and considering a 20-year time-horizon, when it becomes cost-saving (but less so than VoD and VaR) (Figure S25).

If vaccination costs £85 per dose administered then:

- All strategies have a net cost, except for VaR when using the upper-bound baseline and considering a 20-year time-horizon (Figures S22-S24 vs Figure S25).
- Using the lower-bound baseline, VaR's net cost is higher than VoD's over a 10-year time-horizon (Figure S22) but lower over a 20-year time-horizon (Figure S23).
- Using the upper-bound baseline, VaR's net cost is lower than VoD's over both 10- and 20-year time-horizons (and over the latter VaR is cost-saving, as noted above) (Figures S24-S25).

Comparing strategies incrementally, VaR always dominates VoD, and VoA is never incrementally cost-effective relative to VaR (Figures S22-S25; Tables S14-S17).

Using the lower-bound baseline which assumes that behavioural trends stabilise:

- If vaccination costs £18 per dose administered and VaR is feasible then the most cost-effective strategy is VaR (which is more cost-saving than VoD and gains more QALYs than VoD), with the incremental cost-effectiveness ratio (ICER) of VoA relative to VaR being £12.8M/QALY over a 10-year time-horizon (Figure S22, Table S14) and £10.0M/QALY over a 20-year time-horizon (Figure S23, Table S15) (neither of which would not be considered cost-effective by the usual UK criterion of £20,000/QALY); if VaR is not feasible then the most cost-effective strategy is VoD (which is cost-saving), with the ICER of VoA relative to VoD being £158,400/QALY over a 10-year time-horizon (Figure S22, Table S14) and £77,800/QALY over a 20-year time-horizon (Figure S23, Table S15).
- If vaccination costs £85 per dose administered then no strategy is cost-effective over a 10- or 20-year time-horizon (Figures S22 & S23, Tables S14 & S15).

Using the upper-bound baseline which assumes that behavioural trends continue:

- If vaccination costs £18 per dose administered and VaR is feasible then the most cost-effective strategy is VaR (which is more cost-saving than VoD and gains more QALYs than VoD), with the ICER of VoA relative to VaR being £9.2M/QALY over a 10-year time-horizon (Figure S24, Table S16) and £5.6M/QALY over a 20-year time-horizon (Figure S25, Table S17); if VaR is not feasible then the most cost-effective strategy is VoD (which is cost-saving), with the ICER of VoA

relative to VoD being £130,800/QALY over a 10-year time-horizon (Figure S24, Table S16) and £48,700/QALY over a 20-year time-horizon (Figure S25, Table S17).

- If vaccination costs £85 per dose administered then no strategy is cost-effective over a 10-year time-horizon (Figure S24, Table S16), but over a 20-year time-horizon VaR is cost-saving, with the ICER of VoA relative to VaR being £26.9M/QALY (Figure S25, Table S17); if VaR is not feasible then there is no cost-effective strategy.

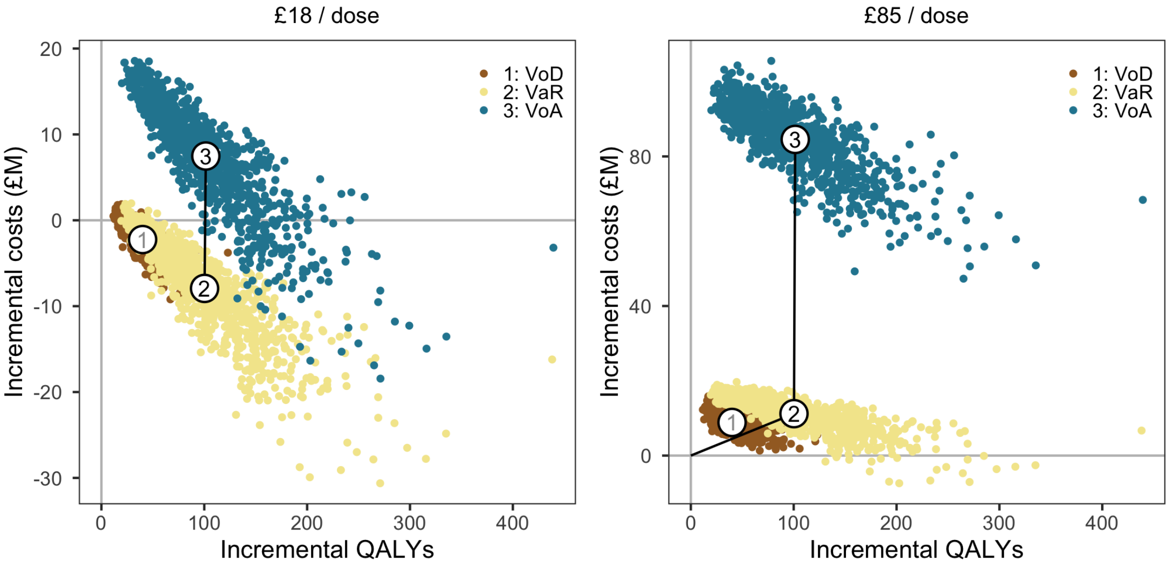

Figure S22: Cost-effectiveness efficiency frontiers for 4CMenB vaccination against gonorrhoea of MSM in England using different targeting strategies, assuming the same effectiveness as MeNZB (31%, 95%CI:21%–39%) and durations of protection as estimated by JCVI for protection of infants against serogroup B meningococcal disease (18 & 36 months after primary vaccination and revaccination, respectively), comparing assumed costs per dose of £18 or £85, and considering a 10-year time-horizon. Labelled points show means for each targeting strategy. Simulations use the lower-bound baseline which assumes that behavioural trends stabilise, using 1000 sets of sampled epidemiological and health-economic parameters, and vaccine uptake of 33·0%(95%CI:32·7%–33·3%).

Table S14: Incremental comparison of targeting strategies using 4CMenB with uptake of 33·0%(95%CI:32·7%–33·3%), assuming the same protection as MeNZB (31%, 95%CI:21%–39%) and durations of protection as estimated by JCVI (18 & 36 months after primary vaccination and revaccination, respectively), using the lower-bound baseline which assumes that behavioural trends stabilise, considering a 10-year time-horizon, and comparing two alternative costs per dose administered (£18 or £85). Each strategy is compared with the previous strategy; VoD is not on the efficiency frontier and is compared with no vaccination. Values reported are means.

**Vaccination strategy**	**Vaccine doses** Undiscounted	Discounted	**Incremental costs (£)** £18/dose	£85/dose	**Incremental QALYs**	**ICER (£/QALY)** £18/dose	£85/dose
None	0	0					
VaR	331,000	285,700	−7,934,700	11,207,100	100·3	cost saving	111,800
VoA	1,019,500	864,500	15,418,100	73,341,400	1·2	12,848,400	61,117,800
Not on the efficiency frontier (incremental to no vaccination):							
VoD	195,400	165,800	−2,245,000	8,863,600	40·1	dominated	dominated

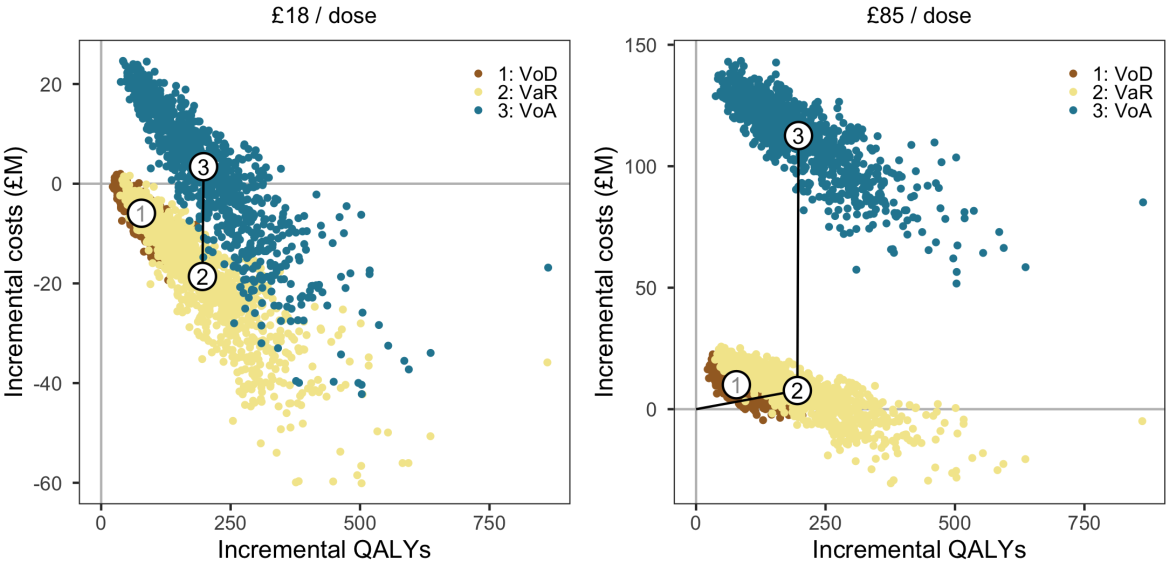

Figure S23: As Figure S22, but considering a 20-year time-horizon.

Table S15: As Table S14, but considering a 20-year time-horizon.

**Vaccination strategy**	**Vaccine doses** Undiscounted	Discounted	**Incremental costs (£)** £18/dose	£85/dose	**Incremental QALYs**	**ICER (£/QALY)** £18/dose	£85/dose
None	0	0					
VaR	514,600	392,300	−18,603,700	7,681,600	195·6	cost saving	39,300
VoA	1,659,900	1,237,600	22,016,400	104,935,200	2·2	10,007,500	47,697,800
Not on the efficiency frontier (incremental to no vaccination):							
VoD	320,300	238,600	−5,926,600	10,056,700	77·8	dominated	dominated

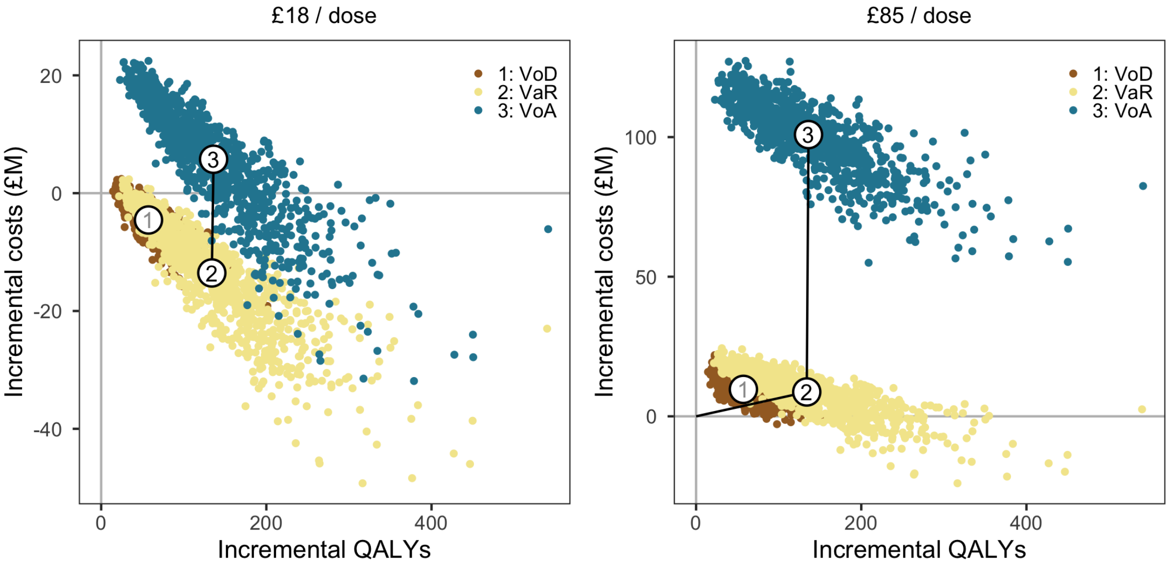

Figure S24: As Figure S22, but using the upper-bound baseline which assumes that behavioural trends continue.

Table S16: As Table S14, but using the upper-bound baseline which assumes that behavioural trends continue.

**Vaccination strategy**	**Vaccine doses** Undiscounted	Discounted	**Incremental costs (£)** £18/dose	£85/dose	**Incremental QALYs**	**ICER (£/QALY)** £18/dose	£85/dose
None	0	0					
VaR	387,500	332,400	−13,545,300	8,723,200	134·0	cost saving	65,100
VoA	1,293,600	1,087,600	19,302,000	92,172,000	2·1	9,191,400	43,891,400
Not on the efficiency frontier (incremental to no vaccination):							
VoD	252,700	212,000	−4,553,200	9,653,400	57·3	dominated	dominated

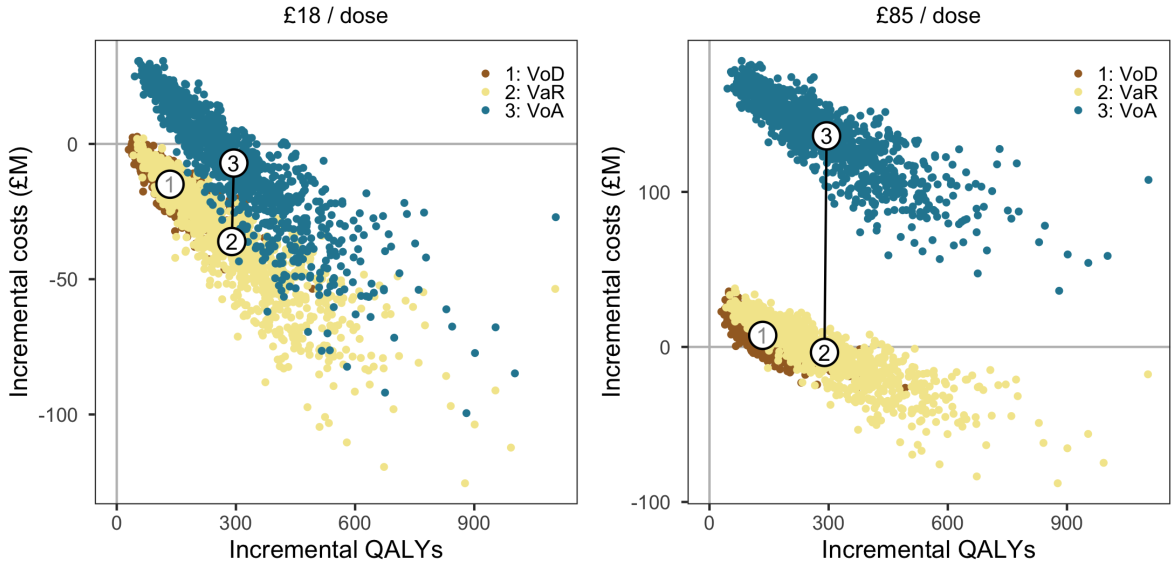

Figure S25: As Figure S24, but considering a 20-year time-horizon.

Table S17: As Table S16, but considering a 20-year time-horizon.

**Vaccination strategy**	**Vaccine doses** Undiscounted	Discounted	**Incremental costs (£)** £18/dose	£85/dose	**Incremental QALYs**	**ICER (£/QALY)** £18/dose	£85/dose
None	0	0					
VaR	652,900	485,800	−36,077,700	−3,530,300	289·6	cost saving	cost saving
VoA	2,265,800	1,651,700	29,002,200	139,669,100	5·2	5,577,300	26,859,400
Not on the efficiency frontier (incremental to no vaccination):							
VoD	462,500	333,500	−14,898,300	7,446,700	134·1	dominated	dominated

2.4.3 Sensitivity to health economic parameters

The estimated value of vaccination is largely robust to uncertainty in the health economic parameters and is most sensitive to the unit cost of treatment.

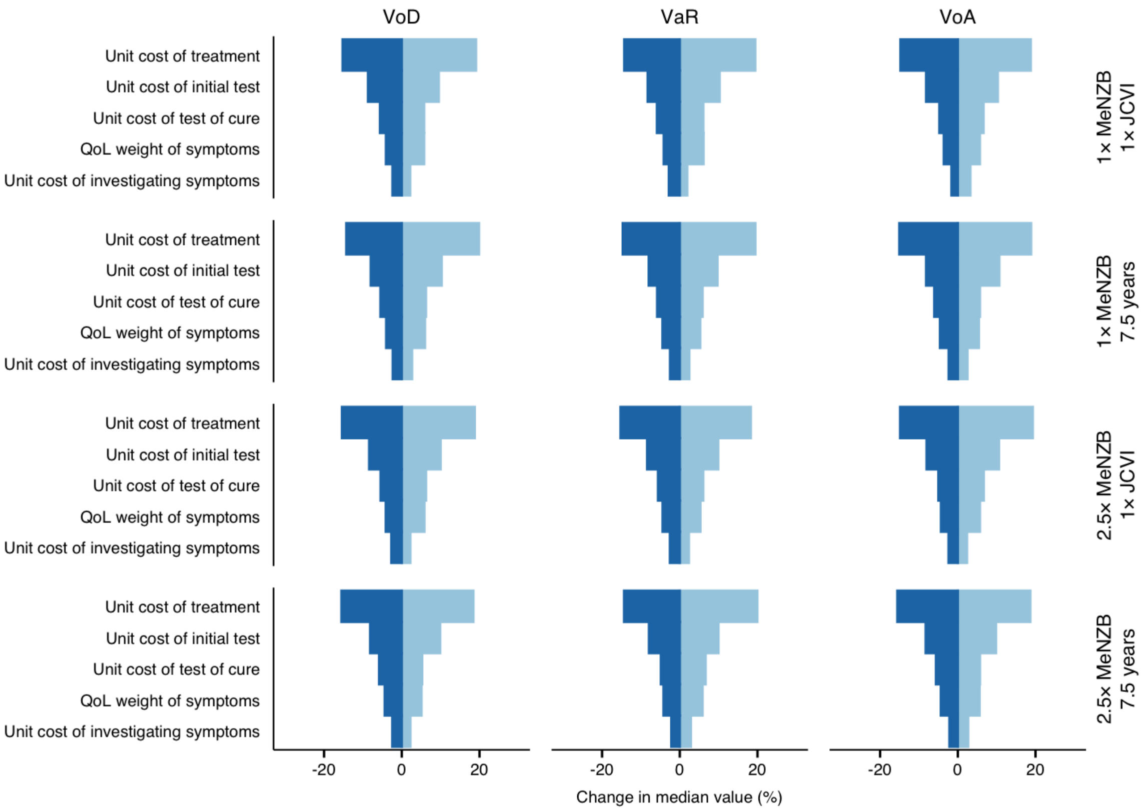

Figure S26: Tornado plots showing univariate sensitivity analysis of the percentage change in the median value of vaccination with 4CMenB when the health-economic parameters are individually set at the 2·5% and 97·5% quantiles of their distributions (as described in Table S4). Rows show combinations of vaccine protection of 1× or 2·5× that of MeNZB (31%, 95%CI:21%–39%); and durations of protection as estimated by JCVI (i.e. 18 & 36 months following primary and re-vaccination, respectively) or 7·5 years following both primary and re-vaccination. Columns show the different targeting strategies. Simulations compare vaccination against the lower-bound baseline which assumes that behavioural trends stabilise, using 1,000 sets of sampled epidemiological and health-economic parameters, with vaccine uptake of 33·0%(95%CI:32·7%–33·3%), and a QALY valued at £20,000; and consider a 10-year time-horizon.

References

[1] Garnett GP, Mertz KJ, Finelli L, Levine WC, St Louis ME. The transmission dynamics of gonorrhoea: modelling the reported behaviour of infected patients from Newark, New Jersey. Philosophical Transactions of the Royal Society of London Series B: Biological Sciences. 1999;354:787–797.

[2] Public Health England. Sexually transmitted infections (STIs): annual data tables;. Available from: https://www.gov.uk/government/statistics/sexually-transmitted-infections-stis-annual-data-tables.

[3] Wetten S, Mohammed H, Yung M, Mercer CH, Cassell JA, Hughes G. Diagnosis and treatment of chlamydia and gonorrhoea in general practice in England 2000-2011: a population-based study using data from the UK Clinical Practice Research Datalink. BMJ Open. 2015;5(e007776).

[4] Public Health England. Gonococcal resistance to antimicrobials surveillance programme report;. Available from: https://www.gov.uk/government/publications/gonococcal-resistance-to-antimicrobials-surveillance-programme-grasp-report.

[5] Whittles LK, White PJ, Didelot X. Estimating the fitness cost and benefit of cefixime resistance in Neisseria gonorrhoeae to inform prescription policy: a modelling study. PLOS Medicine. 2017;14(10):1–20.

[6] Whittles LK, White PJ, Didelot X. Assessment of the potential of vaccination to combat antibiotic resistance in gonorrhea: a modeling analysis to determine Preferred Product Characteristics. Clinical Infectious Diseases. 2020;71(8):1912–1919.

[7] Johnson A, London School of Hygiene and Tropical Medicine Centre for Sexual and Reproductive Health Research, NatCen Social Research, Mercer C. National Survey of Sexual Attitudes and Lifestyles, 2010-2012. [data collection]; 2017.

[8] Office for National Statistics. United Kingdom population mid-year estimate; 2016. Available from: www.ons.gov.uk/peoplepopulationandcommunity/populationandmigration/populationestimates/timeseries/ukpop/pop.

[9] Plummer M, Best N, Cowles K, Vines K. CODA: convergence diagnosis and output analysis for MCMC. R News. 2006;6(1):7–11.

[10] Brooks SPB, Gelman AG. General methods for monitoring convergence of iterative simulations. Journal of Computational and Graphical Statistics. 1998;7(4):434–455.

[11] Fitzjohn RG, Jombart T. odin;. Available from: https://github.com/mrc-ide/odin.

[12] FitzJohn RG, Knock ES, Whittles LK, Perez-Guzman PN, Bhatia S, Guntoro F, et al. Reproducible parallel inference and simulation of stochastic state space models using odin, dust, and mcstate. Wellcome Open Research. 2020;5:288.

[13] MacGregor L, Speare N, Nicholls J, Harryman L, Horwood J, Kesten JM, et al. Evidence of changing sexual behaviours and clinical attendance patterns, alongside increasing diagnoses of STIs in MSM and TPSM. Sexually Transmitted Infections. 2021;97:507–13.

[14] McLean AR, Blower SM. Imperfect vaccines and herd immunity to HIV. Proceedings of the Royal Society B: Biological Sciences. 1993;253:9–13.

[15] Public Health England. Human papillomavirus (HPV) vaccination uptake in gay, bisexual and other men who have sex with men (MSM) National programme: 2018 annual report. London: Public Health England; 2019. Available from: https://assets.publishing.service.gov.uk/government/uploads/system/uploads/attachment_data/file/864727/HPV_MSM_programme_report_2018.pdf.

[16] King EM, Gilson R, Beddows S, Soldan K, Panwar K, Young C, et al. Human papillomavirus DNA in men who have sex with men: type-specific prevalence, risk factors and implications for vaccination strategies. British Journal of Cancer. 2015;112:1585–1593.

[17] Public Health England. Health Protection Report: Vaccine coverage estimates for the meningococcal ACWY (MenACWY) adolescent vaccination programme in schools across England, in 2018/19; and for the GP-based catch-up programme, to end of August 2019. London: Public Health England;

2020. 2. Available from: https://assets.publishing.service.gov.uk/government/uploads/system/uploads/attachment_data/file/953825/hpr0220_menACWY-vc.pdf.

[18] Curtis LA, Burns A. Unit Costs of Health and Social Care 2017. Personal Social Services Research Unit, University of Kent; 2017.

[19] Curtis LA, Burns A. Unit Costs of Health and Social Care 2019. Personal Social Services Research Unit, University of Kent; 2019.

[20] Adams EJ, Ehrlich A, Turner KME, Shah K, Macleod J, Goldenberg S, et al. Mapping patient pathways and estimating resource use for point of care versus standard testing and treatment of chlamydia and gonorrhoea in genitourinary medicine clinics in the UK. BMJ Open. 2014;4(e005322).

[21] Reitsema M, van Hoek AJ, van der Loeff MS, Hoornenborg E, van Sighem A, Wallinga J, et al. Preexposure prophylaxis for men who have sex with men in the Netherlands: Impact on HIV and Neisseria gonorrhoeae transmission and cost-effectiveness. Aids. 2020;34(4):621–630.

[22] Institute of Medicine. Vaccines for the 21st Century: A Tool for Decision Making. Washington, DC: The National Academies Press; 2000. Available from: https://doi.org/10.17226/5501.

[23] National Institute for Health and Care Excellence (NICE). Developing NICE guidelines: the manual; 2014. Available from: https://www.nice.org.uk/Media/Default/About/what-we-do/our-programmes/developing-NICE-guidelines-the-manual.pdf.

[24] Public Health England. Meningococcal B vaccination. Information for healthcare practitioners. London: Public Health England; 2021. Available from: https://assets.publishing.service.gov.uk/government/uploads/system/uploads/attachment_data/file/998409/Meningococcal_B_vaccination_information_for_healthcare_practitioners_July21.pdf.

[25] Petousis-Harris H, Paynter J, Morgan J, Saxton P, McArdle B, Goodyear-Smith F, et al. Effectiveness of a group B outer membrane vesicle meningococcal vaccine against gonorrhoea in New Zealand: a retrospective case-control study. Lancet. 2017;390(10102):1603–1610.

[26] Nolan T, Santolaya ME, de Looze F, Marshall H, Richmond P, Henein S, et al. Antibody persistence and booster response in adolescents and young adults 4 and 7.5 years after immunization with 4CMenB vaccine. Vaccine. 2019;37(9):1209–1218.

[27] Christensen H, Hickman M, Edmunds WJ, Trotter CL. Introducing vaccination against serogroup B meningococcal disease: An economic and mathematical modelling study of potential impact. Vaccine. 2013;31(23):2638–2646.

[28] British National Formulary. Meningococcal group B Vaccine (rDNA, component, absorbed);. Available from: https://bnf.nice.org.uk/medicinal-forms/meningococcal-group-b-vaccine-rdna-component-adsorbed.html.

[29] NHS England. Human Papilloma Virus (HPV) immunisation programme for men that have sex with men (MSM) 2018/19; 2019. Available from: https://www.england.nhs.uk/wp-content/uploads/2019/01/hpv-msm-faqs-for-hcp.pdf.

[30] Baio G, Berardi A, Heath A. Bayesian Cost-Effectiveness Analysis with the R package BCEA. New York, NY: Springer; 2017.

[31] Joint Committee on Vaccination and Immunisation. Code of Practice. Department of Health; 2013. Available from: www.gov.uk/government/uploads/system/uploads/attachment_data/file/224864/JCVI_Code_of_Practice_revision_2013_-_final.pdf.
